# Accurate Calculation of Electron Paramagnetic Resonance Parameters for Molybdenum Compounds

**DOI:** 10.1002/cphc.202500317

**Published:** 2025-10-16

**Authors:** Maria Drosou, Iris Wehrung, Dimitrios A. Pantazis, Maylis Orio

**Affiliations:** ^1^ Max‐Planck‐Institut für Kohlenforschung Kaiser‐Wilhelm‐Platz 1 45470 Mülheim an der Ruhr Germany; ^2^ Department of Chemistry Quantum Chemistry TU Darmstadt Peter‐Grünberg‐Str. 4 64287 Darmstadt Germany; ^3^ Aix Marseille Univ CNRS Centrale Med ISM2 Marseille France

**Keywords:** electron paramagnetic resonance, *g*‐tensors, hyperfine couplings, molybdenum, quantum chemistry

## Abstract

Paramagnetic molybdenum compounds are of great interest in inorganic chemistry and metalloenzyme catalysis. Electron paramagnetic resonance (EPR) spectroscopies that determine hyperfine coupling constants (HFCs) and *g*‐tensor values are essential for investigating the electronic structure of these compounds, but require support from accurate quantum chemical approaches. Here, a database of Mo(V) complexes with well‐defined structures and EPR parameters is presented, and optimal quantum chemical protocols for ^95^Mo HFCs and *g*‐values are investigated. It is shown that unmodified segmented all‐ electron relativistically contracted (SARC) all‐electron basis sets can produce converged results for HFCs and *g*‐values with the exact‐2‐component (X2C) Hamiltonian. The dependence of EPR parameters on the functional is studied in detail. Double‐hybrid functionals and global hybrids with high exact exchange are top performers for ^95^Mo HFCs, with PBE0‐DH achieving the best agreement with experiment. Comparison of density functional theory (DFT)‐derived HFCs with values obtained by coupled cluster theory with the domain‐based local pair natural orbital approach (DLPNO‐CCSD) shows that DFT remains the method of choice for the present set of compounds. Smaller differentiation among functionals is observed for *g*‐tensors, although PBE0‐DH is still a top performer and can be recommended as the most reliable approach overall for describing both valence and core properties of Mo compounds.

## Graphical abstract

Using a curated database of experimentally characterized, biologically relevant Mo(V) compounds, this study evaluates the optimal choice of basis sets, density functionals, and wavefunction methods for accurately computing *g*‐tensor values and ^95^Mo hyperfine coupling constants, with the goal of establishing reliable protocols for predicting electron paramagnetic resonance of molybdenum complexes and enzyme cofactors.

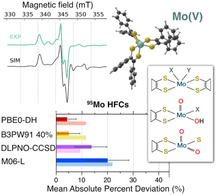

## 1 Introduction

**Figure 1:**
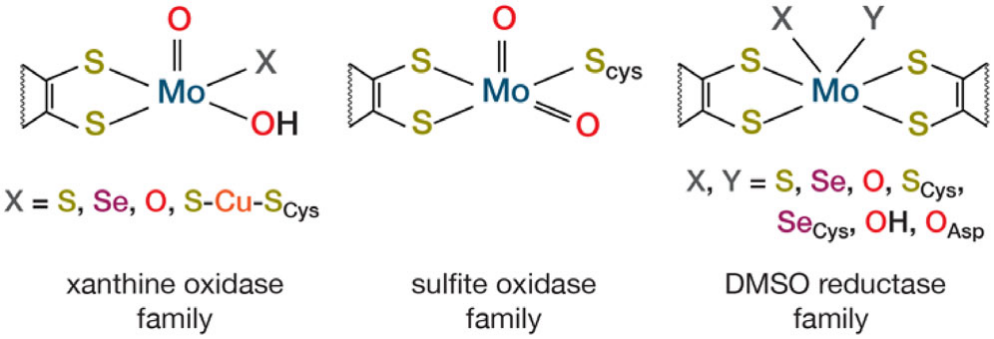
Structures of the Mo coordination sphere for three families of molybdenum enzymes.

Molybdenum is the only known second‐row transition metal essential for all forms of life. Despite a relatively limited set of coordination possibilities encountered in biology, the chemical versatility of the metal means that Mo enzymes are characterized by a wide range of reactivity, catalyzing diverse reactions of central importance in biogeochemical cycles and having important implications in the fields of environment, energy, and human health.^[^ ^1^, ^2^, ^3^, ^4^ ^]^ Specific examples include clusters that take advantage of multimetallic cooperativity, such as the multinuclear Fe_7_Mo cluster of nitrogenase^[^ ^5^ ^]^ and the binuclear MoCu center of CO dehydrogenase,^[^ ^6^ ^]^ but Mo is also found independently in several mononuclear active sites often coordinated by a pyranopterin‐dithiolene ligand. Molybdopterin enzymes are conventionally categorized into three families based on the coordination sphere of the Mo center, namely xanthine oxidase, sulfite oxidase, and dimethyl sulfoxide (DMSO) reductase families (**Figure** **1**), named according to their archetypical member. Formate dehydrogenases,^[^ ^7^, ^8^, ^9^ ^]^ to single out a prominent type of enzyme, belong to the DMSO reductase family and are of particular interest because they catalyze the reduction of CO_2_, a reaction of fundamental significance in the current context of climate crisis mitigation and solar fuels research.^[^ ^10^ ^]^

A main challenge in the field is to understand the structural factors that control the reactivity and substrate specificity of Mo cofactors. Toward this end, it is crucial to reliably map structural features at the atomic level to the experimentally observed spectroscopic properties of these cofactors. This can be facilitated by the combined and complementary approaches of synthetic model chemistry, which provides well‐defined biomimetic molecular compounds,^[^ ^11^, ^12^ ^]^ and by quantum chemistry, which provides the mapping between specific structural features and spectroscopic observables.^[^ ^13^, ^14^ ^]^ The latter can only succeed if reliable and accurate computational methodologies and protocols are available. This represents a continuing quest for applied quantum chemistry in the field of open‐shell transition metal ions. In the case of molybdenum, of particular interest is the oxidation state Mo(V) with a formal 4d^1^ electron configuration (total spin *S* = 1/2) that lends itself to probing by electron paramagnetic resonance (EPR) spectroscopy.^[^ ^12^, ^15^ ^]^

The two quantities of interest in this context are the molybdenum hyperfine coupling constants (HFCs, *A*) and the *g* tensors of the Mo(V) compounds. Both parameters have been targeted by quantum chemical studies in the past,^[^ ^16^, ^17^, ^18^, ^19^, ^20^, ^21^, ^22^, ^23^, ^24^, ^25^ ^]^ with density functional theory (DFT) mostly used to study the sensitivity of computational predictions with respect to the functional, basis set, and the different frameworks to account for relativistic effects. Important observations have been made concerning the capabilities and limitations of representative approaches in capturing the essential physics, leading to practical suggestions such as the requirement for Hartree–Fock exchange admixture (HFX, often referred to also as exact‐exchange EXX) in the range of 30%–40% for global hybrid functionals.^[^ ^26^, ^27^, ^28^ ^]^ Most of these studies have focused on small molecules or only a few representative examples of larger coordination compounds; therefore, an extensive reference set with sufficient diversity and direct relevance to biological systems is still lacking.

In the present work, our first goal is to define such an extensive set of medium‐sized Mo(V) compounds that cover diverse coordination environments with relatively large ligands. After extensive investigation and validation of the primary literature, we compiled a set of 22 Mo(V) compounds that are structurally well‐defined, pose no potential complications due to noninnocent ligands, and have well‐resolved and numerically reliable experimental EPR parameters. Subsequently, we evaluate a large collection of modern quantum chemical approaches for the calculation of ^95^Mo HFCs and *g*‐tensors. Given our aim of establishing a “universal” approach that will be easily applicable to biological systems, typically in the context of multiscale quantum‐mechanics/molecular‐mechanics calculations, we conduct our investigation using the modern exact 2‐component (X2C) Hamiltonian^[^ ^29^, ^30^, ^31^ ^]^ in its scalar (one‐center) form with a finite nucleus model. After determining an optimal choice of basis sets for Mo,^[^ ^32^ ^]^ we evaluate a large selection of functionals, sampling several double‐hybrid variants that have not been investigated before in this context. In addition, we examine the performance of a very recent implementation of the domain‐based local pair natural (DLPNO) approximation^[^ ^33^ ^]^ to coupled cluster with singles and doubles (CCSD) for ^95^Mo HFCs, a wave function approach that is fast enough to be applied to realistic‐size molecules. Our results update the state of the art in terms of practical methodologies for accurate calculations of EPR parameters in Mo systems, highlighting the PBE0‐DH double‐hybrid functional^[^ ^34^ ^]^ as a reliable higher‐rung alternative to modified global hybrid functionals for both core and valence properties.

## 2 Results and Discussion

### 2.1 Definition of a Reference Set of Compounds

**Figure 2:**
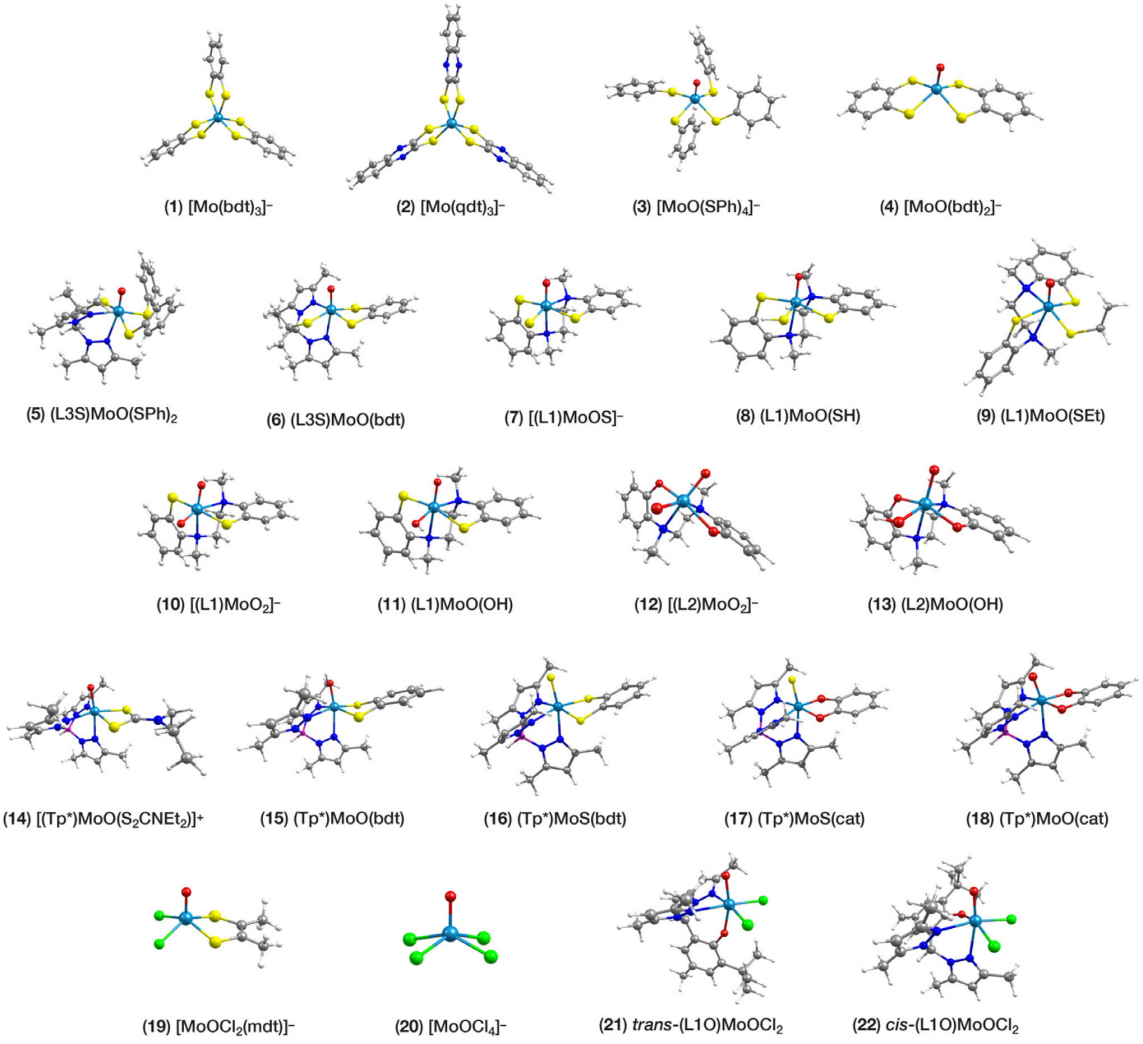
Structures of the Mo(V) complexes considered in the present study. Mo centers are colored light blue; S atoms yellow; O red; N dark blue; B purple; Cl green; C grey; H white. Definition of ligand abbreviations: bdt = benzenedithiolene; qdt = quinoxaline‐2,3‐dithiolene; L3S = (2‐dimethylethanethiol)bis(3,5‐dimethylpyrazolyl)methane; L1 = N,N’‐dimethyl‐N,N’‐bis(2‐mercaptophenyl)‐1,2‐ethylenediamine; L2= N,N’‐dimethyl‐N,N’‐bis(2‐hydroxyphenyl)‐1,2‐ethylenediamine; Tp* = tris‐(3,5‐dimethylpyrazolyl)hydroborate; mdt = 1,2‐dimethyl‐1,2‐dithiolene; L1O = bis(3,5‐dimethylpyrazolyl)‐3‐tert‐butyl‐2‐hydroxy‐5‐methylphenyl)methane.

Although the subject has been approached in past literature,^[^ ^16^, ^17^, ^18^, ^19^, ^20^, ^21^, ^22^, ^23^, ^24^, ^25^ ^]^ there seems to be no standard collection of Mo(V) complexes to serve as a comprehensive reference set. Therefore, our first goal was to define a sufficiently diverse and representative set of Mo‐based mononuclear complexes with reliable experimentally resolved *g*‐tensors and HFCs. After careful examination of the primary sources, we selected 22 compounds to serve as our reference set. The optimized structures of all compounds are shown in **Figure** **2**.

The complexes encompass various Mo coordination environments with S, O, N, and Cl ligand atom types that render our conclusions broadly applicable to a wide range of systems. All complexes have *S* = ½ ground states, with molybdenum being in its Mo(V) oxidation state (4d^1^). Tris‐dithiolene monoanions **1** and **2** feature a distorted trigonal prismatic coordination sphere. Compounds **3**, **4**, **19,** and **20** are square pyramidal Mo‐oxo species in which Mo is five‐coordinate, slightly elevated from the equatorial plane. The remaining compounds are oxo‐molybdenum complexes where Mo is six‐coordinate; among these, complexes **7**, **16**, and **17** feature sulfido‐ligands on the Mo center. Complexes **10** and **12**, along with their protonated forms **11** and **13**, respectively, resemble the sulfite oxidase family of enzymes and their proposed catalytic intermediates. Structures **21** and **22**, on the other hand, represent the *cis* and *trans* geometric isomers of the same compound with an heteroscorpionate ligand. The compounds of the benchmark set that have been crystallographically characterized are: **1**,^[^ ^35^ ^]^ **3**,^[^ ^36^ ^]^ **4**,^[^ ^37^ ^]^ **5**, and **6**,^[^ ^38^ ^]^ **9**,^[^ ^39^ ^]^ **15**–**17**,^[^ ^40^ ^]^ **19**,^[^ ^41^ ^]^ **20**,^[^ ^42^ ^]^ and **21**–**22**.^[^ ^25^ ^]^ For these complexes, the root mean square deviations of the geometrically optimized (TPSSh) structures from the crystallographic structures are smaller than 0.08 Å, which confirms that the optimization protocol should be sufficiently reliable for all optimized structures of our reference set.

The *g*‐tensors and HFCs for all complexes in the benchmark set, as determined by EPR, are presented in **Table** **1**. The *g* tensor components are expressed as shifts (Δ*g*) from the free electron *g*‐value (*g*_e_ = 2.0023), in parts per thousand (ppt). It is important to acknowledge the inherent limitations^[^ ^19^ ^]^ in the reliability of molybdenum HFCs due to the low natural abundance of molybdenum isotopes with magnetic nuclei (^95^Mo: 15.9% and ^97^Mo: 9.6%, both with *I* = 5/2), arising from the use of natural‐abundance samples in the EPR experiments. Nevertheless, the selected compounds have been carefully chosen to ensure the highest possible data quality. Complexes **1**–**2**, **7**–**11**, and **14**–**18** were measured using multifrequency EPR, and for complexes **14** and **19** refined values^[^ ^19^ ^]^ have been considered. The robustness of the dataset is further evidenced by the absence of significant and persistent outliers in the comparison of different methods, even in light of the structural diversity of the complexes, underscoring the reliability of the data. The *g*‐tensors of the selected compounds cover a broad range of values and symmetries, with *g‐*shifts spanning from −57 to 22 ppt and from −248 to −8 ppt for Δ*g*_11_ and Δ*g*_33_, respectively, while *g‐*tensor anisotropy, Δ*g*_11_–Δ*g*_33_, ranges from 2 to 225 ppt. In all complexes, the Mo HFC tensor is predominantly axial, except for complex **1**, where it exhibits rhombic symmetry. The largest HFC component (*A*_11_) varies within the range of 117–227 MHz.

**Table 1:** List of the Mo(V) complexes considered in the present study and their experimentally determined *g*‐shifts (Δ*g*_*ii*_ = *g*_e_ −*g*_*ii*_ in ppt) and ^95^Mo HFCs (*A*_*ii*_ in MHz).

	Complex	Δ*g*_11_	Δ*g*_22_	Δ*g*_33_	*A*_11_	*A*_22_	*A*_33_	Ref.
**1**	[Mo(bdt)_3_]^−^	18	5	−8	117	87	39	[98]
**2**	[Mo(qdt)_3_]^−^	10	4	−9	120	117	21	[98]
**3**	[MoO(SPh)_4_]^−^	18	−20	−20	157	66	66	[99]
**4**	[MoO(bdt)_2_]^−^	20	−18	−26	–	–	–	[100]
**5**	(L3S)MoO(SPh)_2_	12	−36	−56	165	87	81	[38]
**6**	(L3S)MoO(bdt)	14	−30	−53	156	78	72	[38]
**7**	[(L1)MoOS]^‐^	14	−69	−114	160	70	68	[101]
**8**	(L1)MoO(SH)	13	−43	−50	153	69	70	[101]
**9**	(L1)MoO(SEt)	22	−38	−47	175	71	67	[39]
**10**	[(L1)MoO_2_]^−^	−16	−87	−192	206	94	81	[101]
**11**	(L1)MoO(OH)	−22	−55	−59	192	87	73	[101]
**12**	[(L2)MoO_2_]^−^	−23	−105	−248	–	–	–	[102]
**13**	(L2)MoO(OH)	−22	−55	−59	–	–	–	[102]
**14**	[(Tp*)MoO(S_2_CNEt_2_)]^+^	−22	−32	−48	193	90	85a)	[103]
**15**	(Tp*)MoO(bdt)	2	−30	−68	180	78	72	[40]
**16**	(Tp*)MoS(bdt)	−5	−34	−86	177	79	78	[40]
**17**	(Tp*)MoS(cat)	−38	−43	−105	202	90	87	[40]
**18**	(Tp*)MoO(cat)	−34	−36	−83	192	81	78	[40]
**19**	[MoOCl_2_(mdt)]^−^	2	−35	−59	186a)	75a)	75a)	[41]
**20**	[MoOCl_4_]^−^	−37	−37	−56	227	103	103	[104]
**21**	*trans*‐(L1O)MoOCl_2_	−37	−45	−56	226	104	90	[25]
**22**	*cis*‐(L1O)MoOCl_2_	−57	−61	−73	226	105	96	[25]

a) Simulated values were obtained from Fritscher et al.^[^ ^19^ ^]^

### 2.2 Investigation of Mo Basis Sets

To make sure that the subsequent comparison of methods, particularly for ^95^Mo HFCs, is not confounded by errors arising from the incompleteness of the Mo basis set, our first goal is to identify a sufficiently flexible basis set that can be used with all quantum chemical methods (standard and double‐hybrid DFT as well as wave function‐based approaches) and scalar relativistic Hamiltonians. Additionally, it would ideally be a basis set that can be straightforwardly applied for both ^95^Mo hyperfine and *g*‐tensor calculations without having to conduct separate calculations and thus potentially encounter distinct convergence behavior. For this purpose, we first examined the impact of basis set on *g*‐values and HFCs using the B3PW91 functional with 40% HF exchange, which was previously recommended for EPR parameters of Mo(V) complexes.^[^ ^17^, ^22^ ^]^ We examine convergence of core basis functions and addition of polarization functions on the segmented all‐ electron relativistically contracted (SARC) and the x2c families of basis sets at the scalar relativistic level. These initial tests were conducted on compound **1**, [Mo(bdt)_3_]^−^. Results are presented in **Table** **2** and Table S2–S4, Supporting Information.

**Table 2:** Calculated HFCs (total values and individual components, in MHz) of compound 1 obtained using the X2C Hamiltonian with the B3PW91 functional with 40% HFX and finite nucleus using different basis sets for Mo.

Mo basis set	Total			Isotropic		Anisotropic					
	*A*_11_	*A*_22_	*A*_33_	*A* ^FC^	*A* ^PC^	$A_{11}^{\text{SD}}$	$A_{22}^{\text{SD}}$	$A_{33}^{\text{SD}}$	$A_{11}^{\text{SO,an}}$	$A_{22}^{\text{SO},\text{an}}$	$A_{33}^{\text{SO,an}}$
x2c‐TZVPall	113.1	94.0	17.8	63.5	11.5	34.4	16.6	−51.0	3.8	2.5	−6.3
x2c‐TZVPall *s*‐decontracted	134.9	115.6	39.0	85.1	11.5	34.6	16.7	−51.3	3.9	2.5	−6.3
SARC‐DKH‐TZVP	124.4	107.1	38.2	83.2	6.7	31.7	15.4	−47.1	2.9	1.9	−4.8
SARC‐ZORA‐TZVP	116.4	99.1	30.2	75.2	6.8	31.7	15.4	−47.1	2.9	1.9	−4.8
SARC‐DKH‐TZVP *s*‐decontracted + 3s	123.9	106.7	37.7	82.7	6.7	31.7	15.4	−47.1	2.9	1.9	−4.8
SARC‐X2C‐TZVP *s*‐decontracted + 3s	124.0	106.7	37.7	82.7	6.7	31.7	15.4	−47.1	2.9	1.9	−4.8

Focusing on the X2C Hamiltonian, we first compare the results obtained from the x2c‐TZVPall basis set for Mo with the same basis set in which the *s* functions are completely decontracted. The default form of the x2c‐TZVPall basis set contains 8 *s* Gaussian‐type functions (GTFs) in total, with three contracted Gaussian‐type functions (CGTFs) are composed of 9, 4, and 2 primitive Gaussians, i.e., [94211111]*s* functions, while the higher angular momenta are present as [953111]*p*, [621111]*d*, and 1*f*. Given that the Fermi contact term of the HFC is determined by the spin polarization of the *s* orbitals, we compare the default contracted basis set with a version in which we manually fully decontracted the *s*‐space resulting in 20 independent *s* GTFs. Table 2 shows that the Fermi contact term (*A* ^FC^) is very sensitive to the flexibility of the s‐type basis set subspace, with the more flexible decontracted basis set leading to a huge shift of more than 20 MHz. Given that the primitive GTFs themselves in the x2c‐TZVPall basis set for Mo are not obtained in a conventional way but are highly optimized in the default contracted version (i.e., CGTFs contain overlapping primitives), the solution of manually decontracting the basis set for HFC calculations is not optimal.^[^ ^43^ ^]^ The SARC basis sets use instead by default a well‐tempered sequence of primitives that are subsequently loosely contracted into a single CGTF per orbital angular momentum.^[^ ^32^, ^44^, ^45^, ^46^, ^47^, ^48^ ^]^ SARC basis sets for Mo exist in optimized contractions for the DKH2 and ZORA Hamiltonians. These share the same primitive exponents but have different optimized contraction coefficients for each Hamiltonian so that they are identical in their decontracted versions. For the present purposes, we created an adapted version of the SARC basis set for Mo, in which we retained the common primitive GTFs but contracted the CGTFs in each angular momentum subspace in a way that is fully adapted to the X2C Hamiltonian. Subsequently, we discuss all variants, but note that the optimal contraction coefficients for the X2C Hamiltonian are extremely similar to those of the DKH2 Hamiltonian, to the point where the two basis sets are almost indistinguishable. This is not the case with the ZORA Hamiltonian, where CGTFs have obviously distinct contraction coefficients.

The impact of this difference is evident when comparing the SARC‐DKH‐TZVP basis set for Mo with the SARC‐ZORA‐TZVP, both used with the X2C Hamiltonian: The ZORA version produces a significantly different *A* ^FC^ term, although the anisotropic components are very similar. Decontracting the *s*‐CGTF of the SARC‐DKH‐TZVP and even supplanting it by three additional tight (high‐angular momentum) *s* functions produced by multiplying the tighter exponent by 2.5, 6.25, and 15.625, in the spirit of the “core property” adaptation of older basis sets for lighter elements,^[^ ^49^ ^]^ does not provide perceptible changes in the computed properties. Substitution of the DKH‐adapted with the X2C‐adapted higher‐angular momentum CGTFs has no effect either. We note that the *s*‐decontracted x2c‐TZVPall basis sets are closer to the default (and modified) SARC results, confirming that the x2c‐TZVPall basis sets in their default contraction cannot be used for calculations of the target properties.

The choice of a point nucleus versus a finite nucleus, the latter typically modeled by a Gaussian charge distribution, is another methodological choice that can have an impact on the computed HFC values, specifically on the *A* ^FC^ term. Here, we compared the two options for the X2C Hamiltonian and observed that the differences are less than 0.5 MHz for the Fermi contact term, an effect that is rather insignificant in view of other sources of uncertainty in these calculations. Nevertheless, we decided to use the finite nucleus model in all subsequent calculations because it does not incur additional computational cost and is physically more correct. Under normal circumstances, the use of a finite nucleus model may require specific adaptation of the basis set in the form of tighter *s* functions, i.e., primitive GTFs with higher exponents, but the fact that here we opt for the *s*‐decontracted version of an already highly flexible basis set ensures that the basis set is also converged when a finite nucleus model is employed. We also confirmed that the point versus finite nucleus properties are converged with respect to the extension of the *s*‐decontracted space through extra‐tight *s* functions. The results presented in Table 2 and Table S2 and S3, Supporting Information, indicate that the default SARC‐DKH‐TZVP basis set for Mo with its default contraction pattern is fully sufficient for all practical calculations of Mo EPR properties with the X2C Hamiltonian and a finite nucleus model.

The above discussion focused on the HFC, a property that is very sensitive to the flexibility of the basis set, particularly in the core region. The *g*‐tensor depends more sensitively on the description of the valence electronic structure and thus can be sensitive to higher angular momenta. The basis sets we use are already sufficiently polarized; nevertheless, we tested the effect of further extending the high‐angular momentum subspace of the basis set. These calculations confirmed that addition of more and higher angular momentum functions compared to the default polarization functions of the SARC‐DKH‐TZVP basis set has negligible effect on the computed *g*‐tensors (Table S4, Supporting Information). Therefore, the default version of the SARC‐DKH‐TZVP basis set as used for ^95^Mo HFCs is also converged in terms of size for *g*‐tensor calculations of Mo compounds. This allows us to use a common basis set for all EPR properties and this is what will be applied in all calculations reported in the following sections.

### 2.3 ^95^Mo Hyperfine Coupling Constants from DFT

After having established convergence of DFT‐calculated HFCs and *g*‐tensors with respect to the Mo basis set, we now evaluate the performance of various density functional approaches. To this end, we calculated the ^95^Mo HFCs of 19 complexes in our benchmark set, excluding complexes **4**, **12**, and **13**, as their HFCs have not been experimentally determined (Table S5–S31, Supporting Information). We employed a diverse selection of DFT approaches, encompassing various functional families: generalized gradient approximation (GGA) functionals (BP86, BLYP, M06‐L), meta‐GGA (TPSS, r^2^SCAN), hybrid (TPSSh, B3LYP, B3PW91 with a range of HFX percentages from 20 to 50%), range‐separated hybrid functionals (*ω*B97, CAM‐B3LYP, LC‐BLYP, LC‐PBE), and double‐hybrid (B2PLYP, PBE0‐DH, B2GP‐PLYP, *ω*B2PLYP, *ω*B88PP86, Pr^2^SCAN50). For these calculations, we employed the X2C Hamiltonian with the SARC‐DKH‐TZVP basis set on molybdenum, except for the double‐hybrid functionals, where the SARC‐DKH‐TZVPP basis set was used to maximize electron correlation recovery during the MP2 calculation.

The performance of each method for the molybdenum HFCs is evaluated using the absolute percent deviation (APD) relative to the experimental values. This assessment focuses on three key parameters: the isotropic HFC (*A*_iso_), which is the average of the three HF tensor components; the anisotropic difference (*A*_11_–*A*_33_), which is the difference between the largest and smallest HF tensor components; and the largest component of the HF tensor (*A*_11_). The APDs for each of these parameters are calculated as follows.

$$\text{APD}=\;\left|\frac{A^{\text{calc}}-A^{\text{exp}}}{A^{\text{exp}}}\right|\times 100$$

**Figure 3:**
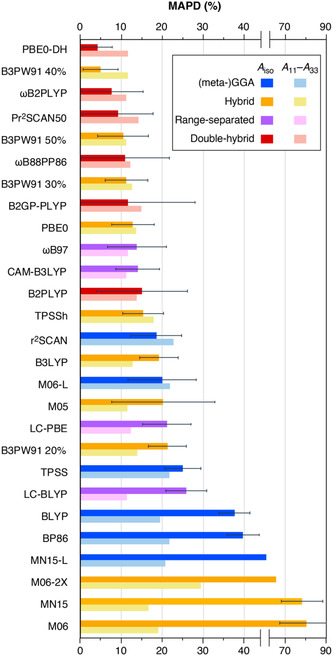
Graphical representation of the MAPDs of *A*_iso_ and *A*_11_–*A*_33_ for the calculated values of the set of 19 Mo(V) complexes obtained with different functionals with respect to experimental results.

The mean APDs (MAPDs) averaged across the 19 examined complexes for each functional, along with their standard deviations, are given in **Table** **3**, and the MAPDs of *A*_iso_ and *A*_11_–*A*_33_ are graphically represented in **Figure** **3**. The functionals are arranged in ascending order of MAPD(*A*_iso_), and in Table 3 they are further categorized according to their respective rungs on the DFT ladder, to enable a more straightforward comparison of methods within the same rung. Among the three criteria, MAPD(*A*_iso_) emerges as the most significant metric due to its significant differentiation among the examined methods. In contrast, as illustrated in Figure 3, MAPD(*A*_11_–*A*_33_) exhibits less variability, as a consequence of the largely systematic nature of the deviations from the experimental values.

**Table 3:** MAPDs of *A*_iso_, *A*_11_–*A*_33_, and *A*_11_ and their standard deviations (SD in MHz) for calculated HFCs using different DFT functionals with respect to experimental HFC values for 19 Mo(V) complexes of the benchmark set.

Functional	*A*_iso_		*A*_11_–*A*_33_		*A*_11_	
	MAPD	SD	MAPD	SD	MAPD	SD
GGA						
BLYP	37.6	3.8	19.5	7.9	31.3	3.0
BP86	39.7	3.9	21.7	7.6	33.4	2.9
MN15‐L	61.2	8.4	20.7	8.9	46.4	4.3
meta‐GGA						
r^2^SCAN	18.5	6.2	22.8	7.6	20.2	4.4
M06‐L	20.0	8.3	21.9	7.9	19.4	8.1
TPSS	25.0	4.4	21.7	7.6	24.1	3.5
Hybrid						
B3PW91 40%	4.9	4.3	11.7	5.7	5.2	4.1
B3PW91 50%	10.4	6.2	11.1	5.4	5.9	4.5
B3PW91 30%	11.2	5.2	12.6	6.6	11.1	4.7
PBE0	12.8	5.2	13.7	7.9	13.1	4.4
TPSSh	15.4	5.0	17.8	8.2	16.5	4.1
B3LYP	19.2	4.7	12.7	7.5	16.7	4.2
M05	20.2	12.6	11.4	7.5	15.8	8.5
B3PW91 20%	21.3	4.6	13.9	8.3	18.7	3.9
M06‐2X	66.4	19.8	29.5	58.7	50.5	37.3
MN15	78.4	10.0	16.6	18.4	49.9	9.5
M06	80.4	12.7	19.0	21.4	57.5	17.8
Range separated						
*ω*B97	13.8	7.2	11.7	6.3	8.5	5.7
CAM‐B3LYP	14.0	5.3	11.2	5.9	11.5	5.3
LC‐PBE	21.1	6.0	12.4	6.1	15.9	5.4
LC‐BLYP	25.8	5.0	11.4	6.5	18.9	5.2
Double hybrid						
PBE0‐DH	4.2	3.5	11.6	6.6	5.5	3.7
*ω*B2PLYP	7.6	7.7	11.3	7.2	6.2	5.1
Pr2SCAN50	9.2	8.5	14.2	7.3	9.5	5.8
*ω*B88PP86	10.9	10.8	12.2	7.2	9.0	7.2
B2GP‐PLYP	11.6	16.4	14.8	12.6	9.3	8.5
B2PLYP	15.0	11.1	13.8	7.7	12.9	5.8

Looking at Figure 3, we observe that double‐hybrid functionals demonstrate the best performance. Among these, PBE0‐DH stands out with the lowest MAPD(*A*_iso_) of 4.2%, while *ω*B2PLYP and Pr^2^SCAN50 also perform well, maintaining MAPD(*A*_iso_) values below 10%. Among all other methods, only the hybrid functional B3PW91 with 40% HFX achieves a comparable MAPD(*A*_iso_) of 4.9%. In terms of standard deviations, PBE0‐DH again excels, achieving the lowest SD(*A*_iso_) of 3.5%, outperforming B3PW91 40%, which has a SD(*A*_iso_) of 4.3%. As shown in Table 3, both PBE0‐DH and B3PW91 40% demonstrate similarly strong performance in the MAPD(*A*_11_–*A*_33_) and MAPD(*A*_11_) criteria, making them the top‐performing methods among those examined.

Regarding hybrid functionals, their MAPDs depend heavily on the HFX percentage. The results confirm previous suggestions that the optimal HFX percentage for HFCs of ^95^Mo complexes is between 30% and 50%, and particularly 40% is strongly favorable in the case of Mo(V) systems. All functionals with HFX percentage smaller than 30% show MAPD(*A*_iso_) values that exceed 12%. These methods tend to underestimate the HF tensor components. In addition, range‐separated functionals perform similarly to hybrid functionals with lower HFX percentages.

GGA and meta‐GGA functionals occupy the bottom half of the plot in Figure 3. Meta‐GGA functionals, however, show a notable improvement over GGA functionals, achieving MAPDs close to 20%, whereas GGA functionals exhibit MAPDs exceeding 30% for both the MAPD(*A*_iso_) and MAPD(*A*_11_) criteria. GGA functionals systematically underestimate the HF tensor components, a consequence of their well‐documented tendency to overestimate the covalency of the M—L bond, leading to an underestimation of the spin density on the Mo nucleus. The systematic nature of the deviations is evident in the relatively small standard deviations of BLYP and BP86, which are comparable to those of PBE0‐DH. This consistency suggests that these very low‐cost methods could be effectively paired with a scaling factor, making them suitable for handling very large systems or for efficiently screening extensive datasets of compounds.

The hybrid Minnesota functionals M06‐2X, MN15, and M06 show by far the worst performances for the HFC calculations, with MAPDs higher than 50% for *A*_iso_ and *A*_11_. Interestingly, the anisotropic parameters *A*_11_–*A*_33_ for MN15 and M06 are not notably larger than those of other methods. However, they exhibit very large standard deviations, indicating that the differences from the experimental values are not systematic. This result mirrors previous reports regarding the poor performance of these functionals for transition metal HFCs.^[^ ^26^, ^41^, ^50^ ^]^ In view of the good performance of Minnesota functionals in thermochemistry, thermochemical kinetics, and noncovalent interactions, the present limitation likely arises from the large number of empirical parameters used in their construction, which constrains the spectrum of properties that they can accurately target.^[^ ^52^ ^]^ Notably, the degree of spin contamination in these functionals is similar to that of B3PW91 40%, with the deviation from the ideal value of 0.75 ranging between 0.003 and 0.070. Moreover, the computed total Mo spin populations are not substantially different from those of other hybrid functionals (see Table S33–S60, Supporting Information), which suggests that the problem likely lies in the poor description of spin polarization in the (semi)core region.^[^ ^24^, ^26^ ^]^

The PBE0‐DH results for all complexes are shown in **Table** **4**. We observe that the largest contributor of the ^95^Mo HF components is the Fermi contact term, *A* ^FC^. Spin‐orbit coupling (SOC) contributions to the isotropic term (***A*** ^**PC**^) are up to 15 MHz and account on average for 7% of the *A*_iso_ term. The rhombic character for compound **1** and the axial character of all other complexes are correctly predicted by the calculations. The predicted anisotropic spin‐orbit contributions also participate in the axial character, especially for **10** in which the $A_{11}^{\text{SO}}$ term is 19.7 MHz.

**Table 4:** Calculated HFCs (total values and individual components, in MHz) obtained using the PBE0‐DH functional for the 22 Mo(V) complexes of the benchmark set.

	Total			Isotropic		Anisotropic					
	*A*_11_	*A*_22_	*A*_33_	*A* ^FC^	*A* ^PC^	$A_{11}^{\text{SD}}$	$A_{22}^{\text{SD}}$	$A_{33}^{\text{SD}}$	$A_{11}^{\text{SO,an}}$	$A_{22}^{\text{SO},\text{an}}$	$A_{33}^{\text{SO,an}}$
**1**	119.3	107.8	37.0	83.2	4.8	28.3	17.5	−45.8	3.0	2.3	−5.3
**2**	115.5	113.6	36.5	83.6	4.9	24.5	22.6	−47.1	2.5	2.5	−5.0
**3**	150.0	64.9	64.9	88.2	5.1	54.7	−27.4	−27.3	2.2	−1.1	−1.1
**5**	159.0	75.8	72.1	96.2	6.1	54.2	−25.6	−28.5	2.7	−1.0	−1.8
**6**	160.1	72.4	69.9	95.3	5.5	56.2	−27.1	−29.1	3.3	−1.4	−1.9
**7**	151.2	74.3	71.5	88.2	10.8	44.6	−22.7	−21.9	7.8	−2.1	−5.6
**8**	162.3	76.0	72.7	97.3	6.4	56.3	−26.7	−29.6	2.5	−1.0	−1.5
**9**	155.7	73.8	68.9	93.2	6.2	54.3	−25.1	−29.2	2.1	−0.6	−1.5
**10**	205.7	99.1	90.4	116.3	15.4	54.4	−26.8	−27.6	19.7	−5.9	−13.8
**11**	185.2	91.1	85.8	111.9	8.8	59.8	−27.7	−32.1	4.9	−2.0	−2.9
**14**	183.7	86.1	83.7	110.8	7.0	61.7	−30.4	−31.3	4.4	−1.5	−2.9
**15**	173.7	81.2	77.5	104.2	6.6	58.2	−27.5	−30.7	4.9	−2.2	−2.7
**16**	166.3	77.2	67.0	96.4	7.0	57.4	−23.9	−33.5	5.6	−2.5	−3.1
**17**	186.9	85.0	82.7	107.8	10.4	62.1	−27.6	−34.5	6.8	−5.7	−1.1
**18**	187.0	81.0	80.7	107.0	9.2	63.7	−33.1	−30.6	7.2	−2.3	−4.9
**19**	179.5	84.3	81.7	108.1	7.0	60.6	−29.0	−31.6	4.0	−2.0	−2.0
**20**	206.4	100.5	100.5	126.1	9.7	65.9	−33.0	−33.0	4.8	−2.4	−2.4
**21**	207.2	105.6	103.8	129.2	9.6	62.3	−30.1	−32.2	6.2	−3.2	−3.0
**22**	209.9	107.5	105.0	129.5	11.3	63.8	−31.4	−32.4	5.5	−2.1	−3.5

**Figure 4:**
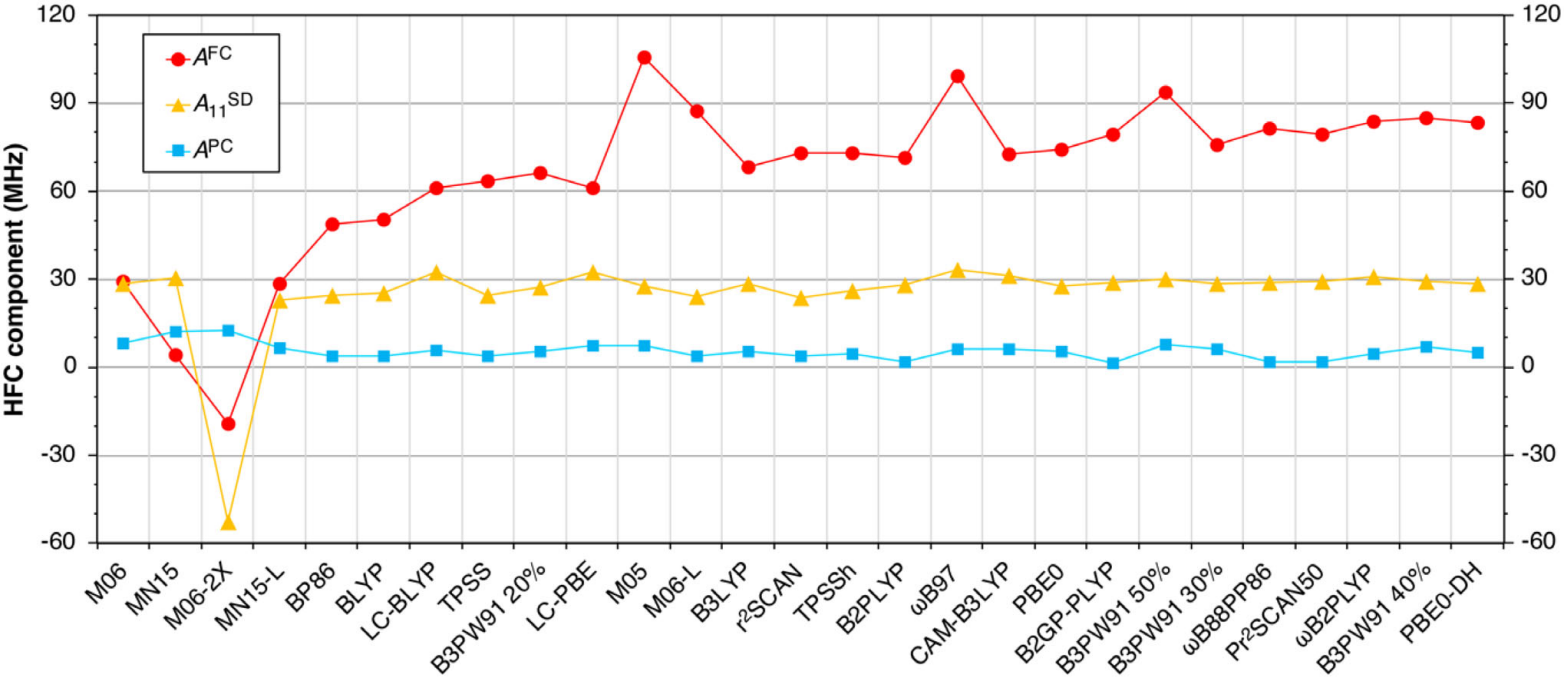
Decomposition of the calculated HFCs for compound **1** obtained using different functionals.

The *A* ^FC^, $A_{33}^{\text{SD}}$, and *A* ^PC^ components of complex **1** calculated using all examined DFT functionals are plotted in **Figure** **4**. The plot shows that the differences between the results obtained using different functionals can be traced back to the calculated Fermi contact term. All functionals predict $A_{11}^{\text{SD}}$ and *A* ^PC^ components, in the range 22.9–33.1 MHz and 1.4 and 7.9 MHz, respectively, except for some Minnesota functionals that are outliers. Therefore, achieving accurate *A* ^FC^ is the biggest methodological challenge for Mo(V) compounds.

**Figure 5:**
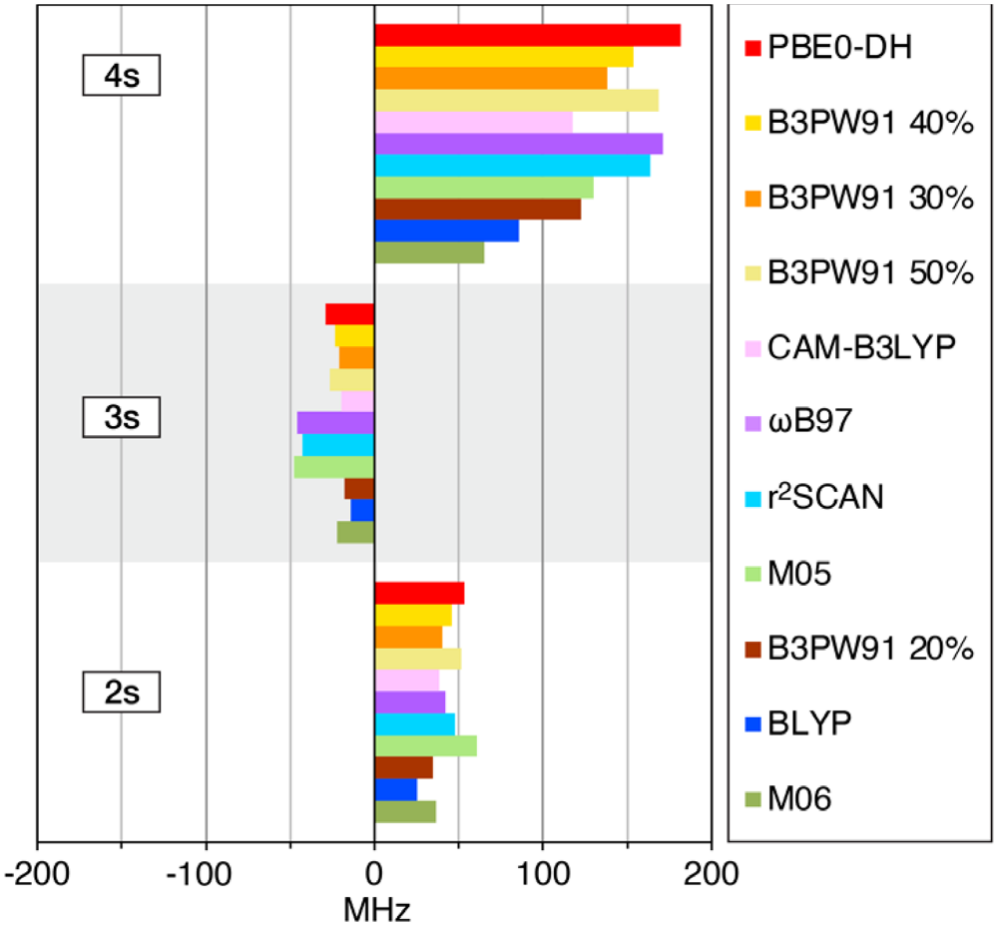
Core orbital contributions for compound **1** using different functionals.

To investigate the origins of the different behaviors of various DFT functionals on the ^95^Mo HFCs, we analyze the molecular orbital contributions to the Fermi contact term, focusing on the core spin polarization. **Figure** **5** shows the molecular orbital analysis of the Mo *A* ^FC^ term of complex **1** obtained using a selection of different DFT approaches. As ^95^Mo has a negative nuclear *g*‐factor, this induces negative spin density at the nucleus from the 2s and 4s orbitals. The poor performance of functionals M06 and BLYP can be correlated with significantly smaller 4s contributions to the Fermi contact term relative to the best‐performing functionals PBE0‐DH and B3PW91 with 40% HFX. Moreover, M06 and M05 predict relatively larger 2s and 3s contributions. This results in lower ratios between the 4s contributions and those of the 2s and 3s orbitals compared to better‐performing methods, resembling previous observations in 3d systems.^[^ ^24^, ^26^ ^]^

The performance of double‐hybrid functionals is likely correlated to the balance between HFX and MP2 contributions. B2PLYP (53% HFX and 27% MP2) has previously demonstrated superior performance for predicting HFCs of transition metal complexes compared to global hybrid functionals,^[^ ^53^ ^]^ suggesting that the MP2 contribution may compensate for exaggerated spin polarization caused by the high HFX percentage. Of course, the balance between HFX and MP2 components is likely system‐dependent, while both components may eventually limit the performance of any given double‐hybrid functional in cases of transition metal systems with significant static correlation (relevant examples have been noted in the case of NMR chemical shifts for 3d transition metal systems).^[^ ^26^ ^]^ For the present Mo(V) systems it appears that PBE0‐DH with 50% HFX and 12.5% MP2 (notably, PBE0‐DH was constructed on a purely theoretical basis without empirical fitting^[^ ^54^ ^]^) achieves the best compromise, while the other double‐hybrid functionals with HFX percentages of 50% or higher and MP2 contributions exceeding 25% lag behind PBE0‐DH.

### 2.4 Coupled Cluster Calculations of ^95^Mo HFCs

The coupled‐cluster method with singles, doubles, and perturbative triples, CCSD(T), commonly regarded as the “gold standard” for quantum chemistry, provides highly accurate HFCs for transition metal complexes.^[^ ^27^ ^]^ However, its steep computational scaling significantly restricts its application to small, simplified systems. In contrast, most of the systems analyzed in this study involve more than 1000 basis functions, making CCSD(T) impractical for such cases. The DLPNO‐CCSD(T) method leverages the locality of electron correlation to reduce the information content of the coupled‐cluster wavefunction. This approach enables the calculation of properties of transition metal complexes that have been notoriously challenging for DFT methods, extending coupled‐cluster accuracy to larger systems, including enzyme models. A recent development is the implementation of the DLPNO‐based Lagrangian scheme, which enables the computation of spin densities that approximate the canonical CCSD densities, allowing the calculation of HFCs.^[^ ^55^ ^]^ While these methods are computationally more demanding than DFT, they have the significant advantage of avoiding system‐dependent biases, thereby offering robust and transferable computational protocols. Aiming to establish a universal approach for enzymes and biomimetic complexes, we assessed the DLPNO‐CCSD method for the prediction of ^95^Mo HFCs. We note that the DLPNO‐CCSD method in its current implementation can only provide the Fermi contact (*A* ^FC^) and spin‐dipolar (*A* ^SD^) terms. In the absence of the spin‐orbit contribution, the total HFCs cannot be obtained directly at this level. The DLPNO‐CCSD results (*A* ^FC^ + *A* ^SD^) are given in **Table** **5**, excluding complexes **16** and **21**, where convergence issues were encountered.

**Table 5:** Calculated HFCs (including the *A* ^FC^ and *A* ^SD^ terms, in MHz) obtained with DLPNO‐CCSD for 17 Mo(V) complexes of the benchmark set.

	*A*_11_	*A*_22_	*A*_33_	Isotropic	Anisotropic		
				*A* ^FC^	$A_{11}^{\text{SD}}$	$A_{22}^{\text{SD}}$	$A_{33}^{\text{SD}}$
**1**	127.6	115.2	42.7	95.2	32.5	20.0	−52.5
**2**	140.6	71.0	50.1	87.3	53.4	−16.2	−37.2
**3**	168.2	77.5	77.2	107.7	60.6	−30.1	−30.4
**5**	171.8	83.1	79.7	111.5	60.3	−28.4	−31.9
**6**	175.6	84.2	82.2	114.0	61.6	−29.8	−31.8
**7**	141.4	72.1	71.7	95.0	46.3	−23.0	−23.3
**8**	176.3	85.3	82.1	114.5	61.7	−29.3	−32.5
**9**	170.6	82.4	77.9	110.3	60.3	−27.9	−32.4
**10**	140.3	112.1	41.8	98.1	42.2	14.1	−56.3
**11**	192.0	96.3	91.3	126.5	65.5	−30.3	−35.2
**14**	192.6	93.4	93.1	126.3	66.2	−33.0	−33.3
**15**	186.9	93.3	90.2	123.4	63.4	−30.2	−33.3
**17**	192.3	96.4	94.8	127.9	64.4	−31.4	−33.0
**18**	191.1	89.2	86.3	122.2	68.9	−33.0	−35.9
**19**	194.7	97.3	94.1	128.7	66.0	−31.4	−34.6
**20**	218.5	113.4	112.9	148.3	70.2	−34.9	−35.3
**22**	216.9	113.2	112.3	147.5	69.4	−34.3	−35.1

**Figure 6:**
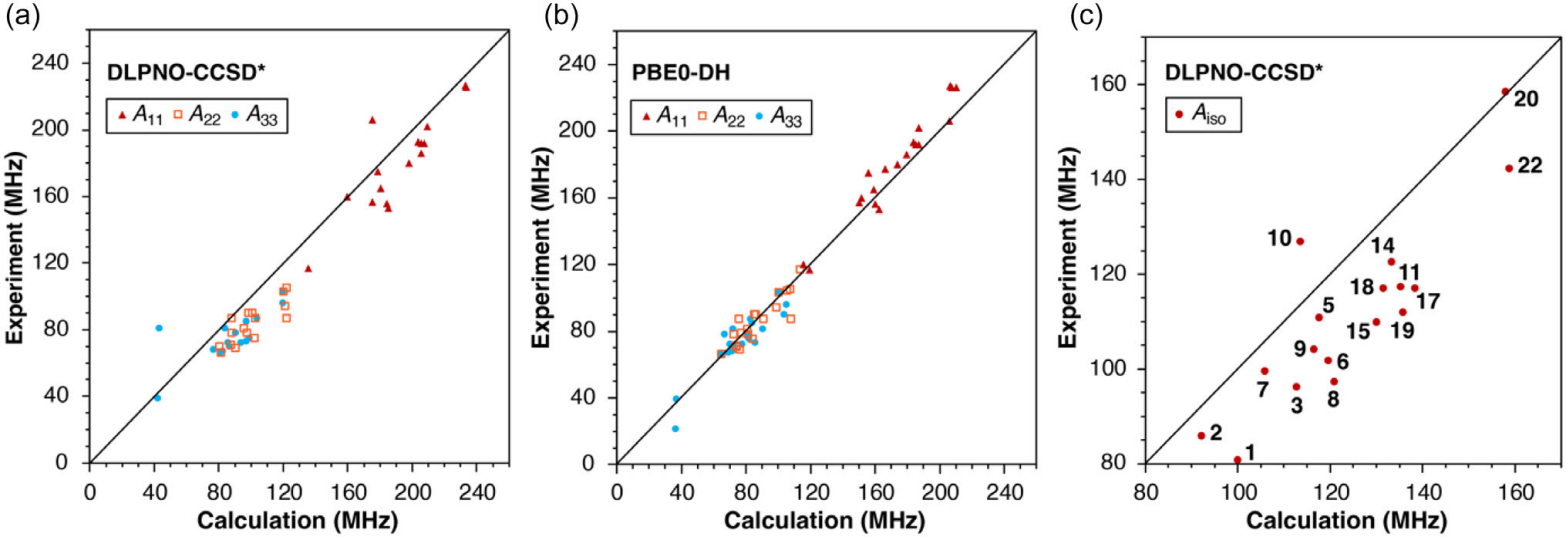
Correlation of experimental and calculated ^95^Mo total HFCs from a) a composite DLPNO‐CCSD* approach and b) from PBE0‐DH, and c) correlation of experimental and calculated isotropic HFCs from the composite DLPNO‐CCSD* method.

To assess the performance of the DLPNO‐CCSD against experiment, we can combine the *A* ^FC^ and *A* ^SD^ terms from DLPNO‐CCSD with the SOC term (*A* ^SO^) derived from another method. Notably, the spin‐orbit contributions to the total HFCs vary less than the Fermi contact and spin‐dipolar terms among the different functionals for all complexes of the benchmark set (see Figure 4 and Table S6–S31, Supporting Information). We define a composite approach denoted DLPNO‐CCSD*, where the total HFCs are computed as a sum of the *A* ^FC^ and the *A* ^SD^ terms calculated with the DLPNO‐CCSD method, and the *A* ^SO^ term provided by the best‐performing DFT functional, PBE0‐DH, so that $A_{ii}(\text{DLPNO‐CCSD}*)=A^{\text{FC}}(\text{DLPNO‐CCSD})+A_{ii}^{\text{SD}}(\text{DLPNO‐CCSD})+A_{ii}^{\text{SO}}(\text{PBE0‐DH})$. Comparisons of experimental and calculated HFC values using DLPNO‐CCSD* and PBE0‐DH are shown in **Figure** **6**a,b, respectively, and numerical values for DLPNO‐CCSD* are given in Table S32, Supporting Information.

Figure 6a shows that the DLPNO‐CCSD* values overestimate the HFC components in most cases. The MAPD(*A*_iso_) for the 17 complexes is 13.5%, with SD of 6.4%, and the MAPD(*A*_11_) is 7.7%, with SD of 4.8%. This places the approach in the mid‐range of DFT methods plotted in Figure 3, specifically between PBE0 and *ω*B97. However, the DLPNO‐CCSD* values have the lowest MAPD(*A*_11_–*A*_33_) of 9.3% with SD of 6.2%. Therefore, despite systematically overestimating the HFC values, DLPNO‐CCSD* most accurately reproduces the anisotropy of the HF tensors.

In Figure 6c, the calculated isotropic HFC contributions (*A*_iso_), derived as a sum of *A* ^FC^ and *A* ^PC^ terms obtained from DLPNO‐CCSD and from PBE0‐DH, respectively, are plotted against the experimental isotropic HFC values. The calculated values are larger than the experimental values for all compounds except **10** and **20**. The *A* ^FC^ contributions obtained from DLPNO‐CCSD exceed those from PBE0‐DH by ≈3–22 MHz for all compounds except **10**, for which DLPNO‐CCSD predicts a lower *A* ^FC^ contribution by 18.2 MHz. Hence, the overestimation of the *A*_iso_ contribution by the composite DLPNO‐CCSD* method results either from the overestimation of the Fermi contact term by DLPNO‐CCSD or from the overestimation of the DFT‐derived pseudocontact term. The second case would imply that the success of PBE0‐DH may be due to compensation between an overestimated pseudocontact term and an underestimated Fermi contact term, but this cannot be determined at present. Overall, at this stage we can conclude that ^95^Mo HFCs obtained with PBE0‐DH are more reliable than those utilizing DLPNO‐CCSD components.

These results indicate that DLPNO‐CCSD holds promise as a method for ^95^Mo HFCs only if future developments address missing spin‐orbit components and current sources of error. Apart from the absence of second‐order contributions, other factors that influence the accuracy of DLPNO‐CCSD include the use of relaxed versus unrelaxed densities and the incorporation of triple excitation corrections (*T*), which has been shown to significantly impact the isotropic HFCs of some small transition metal complexes.^[^ ^27^ ^]^ Addressing these aspects could further enhance the method's accuracy and reliability. Regarding the thresholds used for the DLPNO calculations to select the electron pairs and pair natural orbitals (PNOs) that will be treated at the canonical CCSD level, it is worth noting that the HFC1 settings employed here (see Computational Details) have been optimized specifically for HFC calculations and employ tight thresholds, especially in the core region.^[^ ^55^ ^]^ At this point, and considering that other wave function‐based approaches yield poor estimates for transition metal HFCs,^[^ ^51^ ^]^ DFT remains the method of choice for accurately predicting molybdenum HFCs. Finally, it is important to note that the use of Kohn–Sham orbitals is imperative as initial input in these calculations; Hartree–Fock produces severe artifacts for the present systems, and hence is inappropriate as reference, thus also precluding a meaningful application of pure MP2 methods for the present quantities.

### 2.5 Comparison of DFT Methods for *g*‐Tensors

**Figure 7:**
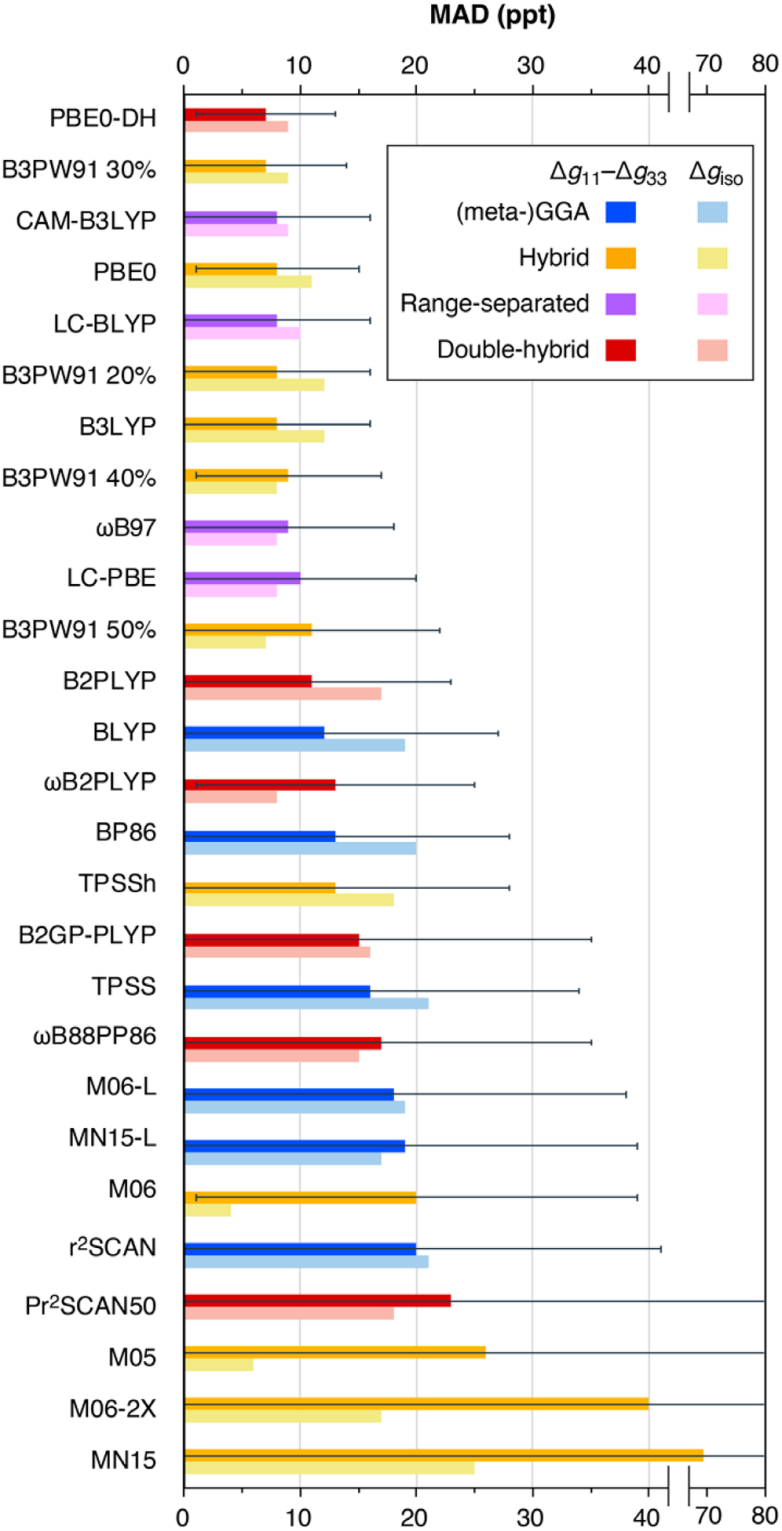
Graphical representation of the MADs of Δ*g*_11_–Δ*g*_33_ and Δ*g*_iso_ for the calculated values of the set of 22 Mo(V) complexes obtained with different functionals with respect to experimental values.

In this section, we compare the performance of various DFT methods for *g*‐tensors of Mo‐based compounds using the 22 complexes of our benchmark set (Table S33–S60, Supporting Information). The experimentally measured *g*‐shifts of our benchmark set span a wide range of 2–248 ppt in absolute values. Hence, we employ the mean absolute deviations (MADs) as the evaluation criterion for comparing different methods, rather than MAPDs. The parameters analyzed include the *g* tensor asymmetry (Δ*g*_11_–Δ*g*_33_), the isotropic *g* value (Δ*g*_iso_), and the *g*‐shift of the largest *g*‐tensor component (Δ*g*_11_). The results are shown in **Table** **6** and graphically represented in **Figure** **7**. Here, the tensor asymmetry is considered as the primary metric, since it is more important for practical applications and is affected to a lesser extent by higher‐order SOC effects, which tend to be systematic.

**Table 6:** MAD and standard deviations (SD in ppt) of Δ*g*_11_–Δ*g*_33_, Δ*g*_iso_, and Δ*g*_11_ for calculated *g*‐tensors using different DFT functionals with respect to experimental values for the 22 Mo(V) complexes of the benchmark set.

Functional	Δ*g*_11_–Δ*g*_33_		Δ*g*_iso_		Δ*g*_11_	
	MAD	SD	MAD	SD	MAD	SD
GGA						
BLYP	12	15	19	11	17	10
BP86	13	15	20	11	18	10
TPSS	16	18	21	13	17	11
meta‐GGA						
M06‐L	18	20	19	15	12	11
MN15‐L	19	20	17	15	11	10
r^2^SCAN	20	21	21	15	13	12
Hybrid						
B3PW91 30%	7	7	9	8	11	9
PBE0	8	7	11	9	12	9
B3PW91 20%	8	8	12	9	13	10
B3LYP	8	8	12	9	13	10
B3PW91 40%	9	8	8	6	10	9
B3PW91 50%	11	11	7	5	11	9
TPSSh	13	15	18	12	14	11
M06	20	19	4	3	10	8
M05	26	29	6	5	8	7
M06‐2X	40	61	17	14	11	11
MN15	69	102	25	31	11	10
Range separated						
CAM‐B3LYP	8	8	9	7	12	9
LC‐BLYP	8	8	10	7	14	9
*ω*B97	9	9	8	7	11	10
LC‐PBE	10	10	8	6	11	11
Double hybrid						
PBE0‐DH	7	6	9	7	10	8
B2PLYP	11	12	17	8	17	12
*ω*B2PLYP	13	12	8	5	11	10
B2GP‐PLYP	15	20	16	8	19	16
*ω*B88PP86	16	17	15	6	18	13
Pr^2^SCAN50	23	51	18	9	14	10

The deviations from the experimental values are in general very small, unlike systems such as Cu‐based complexes for example where deviations tend to be more significant.^[^ ^50^, ^56^, ^57^ ^]^ However, here accuracy is more important to distinguish between different electronic structures. Overall, the results of the nine best‐performing functionals lie in a narrow range with MAD(Δ*g*_11_–Δ*g*_33_) of 7–9 ppt with SDs between 6 and 9 ppt. These functionals are PBE0‐DH, B3PW91 with 30% HFX, PBE0, CAM‐B3LYP, B3PW91 with 20% HFX, B2LYP, LC‐BLYP, B3PW91 with 20% HFX, and *ω*B97. Among them, PBE0‐DH is the only double‐hybrid, whereas all the rest are hybrid functionals. Therefore, the best‐performing functional for the prediction of ^95^Mo HFCs (Figure 3) is also the best‐performing one for *g* tensor prediction (Figure 7). PBE0‐DH has the smallest MAD(Δ*g*_11_–Δ*g*_33_) of 7 ppt with the smallest SD of 6 ppt. Most of the remaining functionals also show relatively small MADs with values less than 20 ppt, but their reliability is further limited due to larger standard deviations. Only the worst‐performing functionals Pr^2^SCAN50, M05, M06‐2X, and MN15 show MAD values that exceed 20 ppt and standard deviations above 50 ppt.

Significant differences are observed in the ranking of methods based on their performance for *g*‐tensors and for molybdenum HFC calculations. Among the double‐hybrid functionals, the standout performer is PBE0‐DH, which excels in both properties. In contrast, B2PLYP ranks in the middle of the plot, while all other double‐hybrid functionals fall in the bottom half. Additionally, all range‐separated hybrid functionals perform very well for *g*‐tensors, whereas for HFC calculations, LC‐BLYP and LC‐PBE exhibit larger deviations compared to most other methods, which highlights the different demands of these properties. Here, the optimal HFX percentage for the hybrid functional B3PW91 is 30%, consistent with the fact that *g*‐tensors require lower HFX percentages than metal HFCs, due to the errors from spin contamination.^[^ ^26^ ^]^

The GGA and the Minnesota functionals collectively exhibit the poorest performances for both *g*‐tensors and HFCs. The GGA functionals tend to generally and systematically overestimate *g*‐tensor components. In contrast, the nature of the deviations observed with the hybrid Minnesota functionals (M05, M06, M06‐2X, and MN15) does not appear to be systematic, which is also reflected on the large standard deviations, which exceed 29 ppt particularly for M05, M06‐2X, and MN15. Notably, M05 and M06 exemplify a remarkable error compensation, achieving the smallest MAD(Δ*g*_iso_) of only 6 and 4 ppt, respectively. This metric does not align with their overall performance, as indicated by their significantly larger MAD(Δ*g*_11_–Δ*g*_33_) of 26 and 20 ppt, respectively, and SDs, which place them among the worst‐performing functionals.

Overall, PBE0‐DH stands out as the only double‐hybrid functional that performs optimally for both HFCs and *g*‐tensor calculations of Mo complexes. Notably, this functional is also among the top‐performers for the particularly challenging problem of copper EPR parameter calculations.^[^ ^57^ ^]^ It remains difficult to identify the physical origin of its success right now, or to directly relate the performance to any specific construction feature of these functionals. A possible reason might be the relatively smaller HFX of PBE0‐DH, 50%, and at the same time the small MP2 (12.5%) contribution, as discussed earlier. This directs toward an optimal construction of double‐hybrid functionals for spin‐dependent properties.

## 3 Conclusions

In this work, we compiled an extensive and varied reference set of Mo compounds with reliable EPR data, and we investigated theoretical methodologies for the calculation of metal (^95^Mo) HFCs and *g*‐values. Our investigation began by determining a suitable basis set for Mo, and we found that the SARC‐DKH‐TZVP basis set can provide converged HFCs and *g*‐values without modifications such as decontraction of the core orbitals or augmentation with additional tight *s* functions. Using this basis set, we conducted a balanced evaluation of quantum chemical methodologies, including DFT and wavefunction‐based approaches. The assessment encompassed a diverse and representative group of DFT families of functionals, including GGA, meta‐GGA, hybrid, range‐separated, as well as double‐hybrid functionals. Among the approaches examined herein, double‐hybrid and range‐separated functionals were tested for ^95^Mo HFCs and *g*‐tensors for the first time.

Our results indicated that the double‐hybrid functional PBE0‐DH is the best‐performing DFT method for both HFCs and *g*‐tensor calculations, as it provides the lowest MADs from the experimental data for both properties. It was also shown that other double‐hybrid functionals such as *ω*B2PLYP, Pr^2^SCAN, *ω*B88PP86, and B2GP‐PLYP also provide small deviations from the experimental HFC values. However, these functionals provide poor estimations of *g*‐tensors. Therefore, only PBE0‐DH succeeds in both properties simultaneously. Regarding hybrid functionals, consistently with previous observations, the HFX of 40% is optimal for HFCs calculations, whereas for *g*‐tensors, a lower HFX of 30% is preferable. Nevertheless, the B3PW91 with HFX 30%–50% provides highly accurate and reliable *g*‐tensors. Thus, the B3PW91 functional with 40% HFX can be proposed as the second‐best method for both properties. The range‐separated functionals achieved accuracy comparable to hybrid functionals with low HFX percentage. They yield poor estimations for HFCs, but perform remarkably well for *g*‐tensors. In line with the well‐known deficiencies of GGA and meta‐GGA functionals that lead them to overestimate the covalency of M—L bonds, they were shown to systematically overestimate *g*‐tensor components and underestimate HFCs with respect to experimental values. The functionals of the Minnesota group cannot deliver reliable predictions for any of the EPR properties examined, and the deviations from the experimental values are not systematic, especially for the hybrid functionals M05, M06, M06‐2X, and MN15. These results for the present d^1^ systems are largely consistent with previously reported benchmark studies on EPR parameters of d^9^ Cu(II) complexes.^[^ ^51^, ^57^ ^]^ However, they should not be considered generalizable to all transition metal cases and other electron configurations, which may be affected by issues such as high spin contamination.^[^ ^26^ ^]^ Ultimately, local hybrid approaches that incorporate position‐dependent HFX admixture may emerge as the most general practical solution to the problem of balancing the description of core and valence spin polarization,^[^ ^26^, ^58^ ^]^ hile other promising approaches based on the postKohn−Sham random phase approximation (RPA) methods^[^ ^59^ ^]^ also require further investigation in large transition metal complexes.

The performance of a wavefunction‐based method, the local variant of coupled‐cluster, DLPNO‐CCSD, was also assessed. Currently, this approach provides only the Fermi contact and spin‐dipolar terms of the HFCs. To compare the HFCs obtained with DLPNO‐CCSD with the experimental values, we used the SOC contribution obtained from the PBE0‐DH functional that achieves optimal performance. The accuracy of this composite method is mediocre and, hence, it does not compete with the performance of double‐hybrid and hybrid functionals with HFX percentages higher than 30%.

Overall, the PBE0‐DH functional is suggested as the best approach for robust and reliable calculations of EPR parameters of Mo complexes that can be used in bioinorganic chemistry applications on enzymes. Further investigation of the construction parameters of PBE0‐DH that makes it so accurate would be interesting for future developments.

## 4 Experimental Section

All calculations were performed with the Orca 6.0 program.^[^ ^60^ ^]^ Geometry optimizations were carried out with the TPSSh^[^ ^61^ ^]^ functional and the x2c‐TZVPall basis sets.^[^ ^43^ ^]^ To reduce computational time the resolution of identity approximation was used^[^ ^62^ ^]^ along with the chain of spheres in combination with automatically generated auxiliary basis sets with the “AutoAux” procedure in Orca.^[^ ^63^ ^]^ Tight convergence criteria were imposed using the TightSCF keyword along with the default (DefGrid2) integration grids. Relativistic effects were included using the exact two‐component (X2C) Hamiltonian.^[^ ^29^, ^30^, ^31^ ^]^ For the complexes of the benchmark set that are crystallographically characterized, crystallographic coordinates were obtained from the Cambridge Structural Database^[^ ^64^ ^]^ and were used as starting coordinates for the geometry optimizations, after being individually edited for chemical correctness and to remove solvent molecules and noncoordinating counter‐ions. At first, only the positions of the hydrogen atoms were optimized, and subsequently, all atoms were relaxed.

The DFT methods evaluated in the present work for the computation of *g*‐tensors and ^95^Mo hyperfine coupling tensors included the GGA functionals BP86^[^ ^65^, ^66^ ^]^ and BLYP,^[^ ^66^, ^67^ ^]^ MN15‐L,^[^ ^68^ ^]^ the meta‐GGA TPSS,^[^ ^69^ ^]^ M06‐L,^[^ ^70^ ^]^ and r^2^SCAN,^[^ ^71^ ^]^ the hybrid meta‐GGA TPSSh,^[^ ^61^ ^]^ the hybrid B3LYP,^[^ ^66^, ^72^ ^]^ PBE0,^[^ ^73^ ^]^ and B3PW91^[^ ^72^, ^74^, ^75^, ^76^ ^]^ with a range of HFX percentages from 20 to 50%, M05,^[^ ^77^ ^]^ M06,^[^ ^78^ ^]^ M06‐2X,^[^ ^78^ ^]^ and MN15,^[^ ^79^ ^]^ the range‐separated hybrid *ω*B97,^[^ ^80^ ^]^ CAM‐B3LYP,^[^ ^81^ ^]^ LC‐BLYP, and LC‐PBE,^[^ ^82^ ^]^ and the double‐hybrid functionals B2PLYP,^[^ ^83^ ^]^ PBE0‐DH,^[^ ^34^ ^]^ B2GP‐PLYP,^[^ ^84^ ^]^ *ω*B2PLYP,^[^ ^85^ ^]^ *ω*B88PP86,^[^ ^86^ ^]^ and Pr^2^SCAN50.^[^ ^87^ ^]^ For calculations with double‐hybrid functionals, all electrons were included in the MP2 correlation treatment (NoFrozenCore keyword in Orca), and “relaxed” densities were used for the EPR parameter calculations. All EPR parameter calculations were carried out using increased integration grids (DefGrid3 settings in Orca). Functionals M05, MN15, and MN15‐L were provided by LibXC.^[^ ^88^ ^]^

For the calculation of EPR parameters, we examined different methods for scalar relativity, including the ZORA^[^ ^89^, ^90^, ^91^ ^]^ and X2C^[^ ^29^, ^30^, ^31^ ^]^ Hamiltonians, along with suitable basis sets. Note that we used a scalar relativistic version of the X2C Hamiltonian, which is effectively a one‐component method. The X2C Hamiltonian is different from ZORA mainly in that the decoupling of the one‐electron Dirac Hamiltonian is exact, whereas in ZORA it is approximate. Nevertheless, ZORA and X2C Hamiltonians predict very similar Mo HFCs, with differences smaller than 2 MHz (Table S2–S4, Supporting Information).

The Mo basis set used for the EPR parameter calculations was carefully selected based on extensive testing, as discussed in the next section. Calculations with ZORA were performed using the def2‐ZORA‐TZVP basis sets^[^ ^92^ ^]^ on the ligands and the SARC‐ZORA‐TZVP basis set on Mo.^[^ ^32^ ^]^ Calculations with X2C were performed using the x2c‐TZVPall basis sets^[^ ^43^ ^]^ on the ligands and we examined the x2c‐TZVPall and SARC‐DKH‐TZVP basis set on Mo. We note that neither of them was designed in their original form for calculations of EPR properties. A SARC basis set for Mo was also explicitly created for the X2C Hamiltonian. To derive such a basis set, starting from the fully decontracted (universal) SARC‐TZVP basis set, we calculated contraction coefficients using the X2C Hamiltonian, which resulted in a new basis set that we note SARC‐X2C‐TZVP in the main text. In all cases, the *s*‐decontracted SARC‐DKH‐TZVP basis set with three additional tight functions^[^ ^49^ ^]^ is used as a reference. The type of nuclear model used can be important for the calculation of HFCs.^[^ ^93^ ^]^ Therefore, we compared the point nucleus and finite nucleus^[^ ^94^ ^]^ approximations for the X2C Hamiltonian, opting for the latter in all method comparisons. In calculations with double‐hybrid functionals and in coupled cluster calculations, the SARC‐DKH‐TZVPP basis set was used for Mo.

The SOC operator was treated by a mean‐field approximation to the Breit–Pauli operator (SOCType 3 in ORCA).^[^ ^95^ ^]^ Construction of the effective potential included one‐electron terms, the Coulomb term was computed exactly, exchange was incorporated via one‐center exact integrals including spin‐other orbit interactions, and local DFT correlation was included with VWN5 (all these choices are set with the keyword “SOCFlags 1,4,3,1” in ORCA).

The ^95^Mo hyperfine coupling tensors were also computed with the DLPNO‐CCSD method using the “unrelaxed” densities.^[^ ^55^, ^96^ ^]^ For these calculations, the Kohn–Sham orbitals were used to construct the reference determinant. The default DLPNO thresholds^[^ ^55^ ^]^ for hyperfine coupling calculations were used (HFC1 settings in Orca). These settings involved a tighter PNO threshold for the core electrons, i.e., *T*_CutPNOCore_ = 1.0 × 10^−3^ instead of 1.0 × 10^−2^ in the NormalPNO and TightPNO settings. Other notable threshold values were *T*_CutMKN_ = 1.0 × 10^−4^, *T*_CutPairs_ = 1.0 × 10^−4^, *T*_CutPNOSingles_ = 0.00, and *T*_CutPNO_ = 1.0 × 10^−7^. In these calculations, all electrons were treated as active (NoFrozenCore).

The HFC arises from three distinct contributions with different physical origins, which are the Fermi contact term (*A* ^FC^), the spin‐dipolar term (*A* ^SD^), and the SOC term (*A* ^SO^). The *A* ^FC^ and *A* ^SD^ terms are first‐order properties. The *A* ^FC^ is an isotropic term arising from spin polarization at the *s* orbitals of Mo and is proportional to the spin density at the nucleus. The *A* ^SD^ is purely anisotropic and arises from dipole‐dipole interactions between the nuclear spin and the spin density in other orbitals (Mo *p*, *d*, *f* and orbitals of other atoms). The *A* ^SO^ term arises predominantly from second‐order contributions, while higher‐order SOC effects contribute less than 2% of HFC components and are therefore less important for Mo than for heavier elements,^[^ ^97^ ^]^ such as W.^[^ ^20^ ^]^ Here, the *A* ^SO^ term has been computed up to second‐order. The *A* ^SO^ term contains both an isotropic and an anisotropic contribution. For better comparison with experimental values, the *A* ^SO^ term obtained from Orca ($A_{ii}^{\text{SO}}$, *i* = 1, 2, 3) has been separated into an isotropic term, known as pseudocontact term *A* ^PC^, and an anisotropic term, 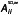, such that: $A_{ii}^{\text{SO}}=A^{\text{PC}}+A_{ii}^{\text{SO,an}}$. Using these definitions, the components *A*_*ii*_ of the complete HFC tensor—up to second‐order perturbation theory—can be written as: *A*_*ii*_ = *A*_iso_ + *A*_an_, where the isotropic HFC contribution *A*_iso_ corresponds to: *A*_iso_ = *A* ^FC^ + *A* ^PC^, and the anisotropic contribution *A*_an_ is: $A_{\text{an}}=A_{ii}^{\text{SD}}+A_{ii}^{\text{SO},\text{an}}$.

## Conflict of Interest

The authors declare no conflict of interest.

## Supporting information

Supplementary Material

## Data Availability

The data that support the findings of this study are openly available in Edmond at https://doi.org/10.17617/3.CVNSRT.

## References

[1] Y. Zhang , S. Rump , V. N. Gladyshev , Coord. Chem. Rev. 2011, 255, 1206. 22451726 10.1016/j.ccr.2011.02.016 PMC3311541

[2] Y. Zhang , V. N. Gladyshev , J. Mol. Biol. 2008, 379, 881. 18485362 10.1016/j.jmb.2008.03.051 PMC2670968

[3] R. Hille , J. Hall , P. Basu , Chem. Rev. 2014, 114, 3963. 24467397 10.1021/cr400443z PMC4080432

[4] M. C. Metzger , P. Basu , In Microbial Metabolism of Metals and Metalloids (Ed: C. J. Hurst ), Springer International Publishing, Cham 2022, pp. 359–415.

[5] O. Einsle , D. C. Rees , Chem. Rev. 2020, 120, 4969. 32538623 10.1021/acs.chemrev.0c00067 PMC8606229

[6] H. Dobbek , L. Gremer , R. Kiefersauer , R. Huber , O. Meyer , Proc. Natl. Acad. Sci. U. S. A. 2002, 99, 15971. 12475995 10.1073/pnas.212640899 PMC138549

[7] S. T. Stripp , B. R. Duffus , V. Fourmond , C. Léger , S. Leimkühler , S. Hirota , Y. Hu , A. Jasniewski , H. Ogata , M. W. Ribbe , Chem. Rev. 2022, 122, 11900. 35849738 10.1021/acs.chemrev.1c00914 PMC9549741

[8] T. Hartmann , N. Schwanhold , S. Leimkühler , BBA ‐ Proteins Proteomics 2015, 1854, 1090. 25514355 10.1016/j.bbapap.2014.12.006

[9] L. B. Maia , I. Moura , Enzymes For Solving Humankind's Problems: Natural And Artificial Systems In Health, Agriculture, Environment And Energy (Eds: J. J. G. Moura , I. Moura , L. B. Maia ), Springer International Publishing, Cham 2021, pp. 29–81.

[10] A. M. Appel , J. E. Bercaw , A. B. Bocarsly , H. Dobbek , D. L. DuBois , M. Dupuis , J. G. Ferry , E. Fujita , R. Hille , P. J. A. Kenis , C. A. Kerfeld , R. H. Morris , C. H. F. Peden , A. R. Portis , S. W. Ragsdale , T. B. Rauchfuss , J. N. H. Reek , L. C. Seefeldt , R. K. Thauer , G. L. Waldrop , Chem. Rev. 2013, 113, 6621. 23767781 10.1021/cr300463y PMC3895110

[11] S. J. N. Burgmayer , M. L. Kirk , Molecules 2023, 28, 7456. 38005178 10.3390/molecules28227456 PMC10673323

[12] C. G. Young , J. Inorg. Biochem. 2016, 162, 238. 27432259 10.1016/j.jinorgbio.2016.06.010

[13] M. Orio , D. A. Pantazis , Chem. Commun. 2021, 57, 3952. 10.1039/d1cc00705j 33885698

[14] V. Barone , S. Alessandrini , M. Biczysko , J. R. Cheeseman , D. C. Clary , A. B. McCoy , R. J. DiRisio , F. Neese , M. Melosso , C. Puzzarini , Nat. Rev. Methods Primers 2021, 1, 38.

[15] L. B. Maia , I. Moura , J. J. G. Moura , in Future Directions In Metalloprotein And Metalloenzyme Research (Eds.: G. Hanson , L. Berliner ), Springer International Publishing, Cham 2017, pp. 55–101.

[16] M. M. Cosper , F. Neese , A. V. Astashkin , M. D. Carducci , A. M. Raitsimring , J. H. Enemark , Inorg. Chem. 2005, 44, 1290. 15732969 10.1021/ic0483850

[17] J. Fritscher , P. Hrobárik , M. Kaupp , J. Phys. Chem. B 2007, 111, 4616. 17408258 10.1021/jp070638y

[18] S. C. Drew , C. G. Young , G. R. Hanson , Inorg. Chem. 2007, 46, 2388. 17305330 10.1021/ic060586b

[19] J. Fritscher , P. Hrobárik , M. Kaupp , Inorg. Chem. 2007, 46, 8146. 17725345 10.1021/ic070341e

[20] P. Hrobárik , O. L. Malkina , V. G. Malkin , M. Kaupp , Chem. Phys. 2009, 356, 229.

[21] R. G. Hadt , V. N. Nemykin , J. G. Olsen , P. Basu , Phys. Chem. Chem. Phys. 2009, 11, 10377. 19890522 10.1039/b905554a PMC2879133

[22] S. Gohr , P. Hrobárik , M. Repiský , S. Komorovský , K. Ruud , M. Kaupp , J. Phys. Chem. A 2015, 119, 12892. 26636191 10.1021/acs.jpca.5b10996

[23] A. Wodyński , M. Kaupp , J. Phys. Chem. A 2019, 123, 5660. 31184482 10.1021/acs.jpca.9b03979

[24] A. Wodyński , M. Kaupp , J. Chem. Theory Comput. 2020, 16, 314. 31834796 10.1021/acs.jctc.9b00911

[25] V. N. Nemykin , J. R. Sabin , B. W. Kail , A. Upadhyay , M. P. Hendrich , P. Basu , J. Inorg. Biochem. 2023, 245, 112228. 37149488 10.1016/j.jinorgbio.2023.112228 PMC10330323

[26] C. J. Schattenberg , T. M. Maier , M. Kaupp , J. Chem. Theory Comput. 2018, 14, 5653. 30299950 10.1021/acs.jctc.8b00597

[27] M. Munzarová , M. Kaupp , J. Phys. Chem. A 1999, 103, 9966.

[28] M. L. Munzarová , P. Kubáček , M. Kaupp , J. Am. Chem. Soc. 2000, 122, 11900.

[29] D. Peng , N. Middendorf , F. Weigend , M. Reiher , J. Chem. Phys. 2013, 138, 184105. 23676027 10.1063/1.4803693

[30] Y. J. Franzke , N. Middendorf , F. Weigend , J. Chem. Phys. 2018, 148, 104110. 29544265 10.1063/1.5022153

[31] Y. J. Franzke , J. M. Yu , J. Chem. Theory Comput. 2022, 18, 323. 34928142 10.1021/acs.jctc.1c01027

[32] J. D. Rolfes , F. Neese , D. A. Pantazis , J. Comput. Chem. 2020, 41, 1842. 32484577 10.1002/jcc.26355

[33] C. Riplinger , F. Neese , J. Chem. Phys. 2013, 138, 034106. 23343267 10.1063/1.4773581

[34] E. Brémond , C. Adamo , J. Chem. Phys. 2011, 135, 024106. 21766924 10.1063/1.3604569

[35] A. Cervilla , E. Llopis , D. Marco , F. Pérez , Inorg. Chem. 2001, 40, 6525. 11720515 10.1021/ic010327g

[36] J. R. Bradbury , M. F. Mackay , A. G. Wedd , Aust. J. Chem. 1978, 31, 2423.

[37] S. Boyde , S. R. Ellis , C. D. Garner , W. Clegg , J. Chem. Soc., Chem. Commun. 1986, 20, 1541.

[38] K. Peariso , B. S. Chohan , C. J. Carrano , M. L. Kirk , Inorg. Chem. 2003, 42, 6194. 14514295 10.1021/ic034478q

[39] M. L. Mader , M. D. Carducci , J. H. Enemark , Inorg. Chem. 2000, 39, 525. 11229572 10.1021/ic990768o

[40] S. C. Drew , J. P. Hill , I. Lane , G. R. Hanson , R. W. Gable , C. G. Young , Inorg. Chem. 2007, 46, 2373. 17343374 10.1021/ic060585j

[41] B. S. Lim , M. W. Willer , M. Miao , R. H. Holm , J. Am. Chem. Soc. 2001, 123, 8343. 11516283 10.1021/ja010786g

[42] C. D. Garner , L. H. Hill , F. E. Mabbs , D. L. McFadden , A. T. McPhail , J. Chem. Soc., Dalton Trans. 1977, 9, 853.

[43] P. Pollak , F. Weigend , J. Chem. Theory Comput. 2017, 13, 3696. 28679044 10.1021/acs.jctc.7b00593

[44] M. Bühl , C. Reimann , D. A. Pantazis , T. Bredow , F. Neese , J. Chem. Theory Comput. 2008, 4, 1449. 26621431 10.1021/ct800172j

[45] D. A. Pantazis , X.‐Y. Chen , C. R. Landis , F. Neese , J. Chem. Theory Comput. 2008, 4, 908. 26621232 10.1021/ct800047t

[46] D. A. Pantazis , F. Neese , J. Chem. Theory Comput. 2009, 5, 2229. 26616609 10.1021/ct900090f

[47] D. A. Pantazis , F. Neese , J. Chem. Theory Comput. 2011, 7, 677.

[48] D. A. Pantazis , F. Neese , Theor. Chem. Acc. 2012, 131, 1292.

[49] F. Neese , Inorg. Chim. Acta 2002, 337, 181.

[50] G. Sciortino , G. Lubinu , J.‐D. Maréchal , E. Garribba , Magnetochemistry 2018, 4, 55.

[51] R. J. Gómez‐Piñeiro , D. A. Pantazis , M. Orio , ChemPhysChem 2020, 21, 2667. 33201578 10.1002/cphc.202000649 PMC7756273

[52] N. Mardirossian , M. Head‐Gordon , J. Chem. Theory Comput. 2016, 12, 4303. 27537680 10.1021/acs.jctc.6b00637

[53] S. Kossmann , B. Kirchner , F. Neese , Mol. Phys. 2007, 105, 2049.

[54] M. G. Medvedev , I. S. Bushmarinov , J. Sun , J. P. Perdew , K. A. Lyssenko , Science 2017, 355, 49. 28059761 10.1126/science.aah5975

[55] M. Saitow , F. Neese , J. Chem. Phys. 2018, 149, 034104. 30037259 10.1063/1.5027114

[56] R. J. Gómez‐Piñeiro , M. Drosou , S. Bertaina , C. Decroos , A. J. Simaan , D. A. Pantazis , M. Orio , Inorg. Chem. 2022, 61, 8022. 35549254 10.1021/acs.inorgchem.2c00766 PMC9131454

[57] M. Drosou , C. A. Mitsopoulou , M. Orio , D. A. Pantazis , Magnetochemistry 2022, 8, 36.

[58] A. Wodyński , B. Lauw , M. Reimann , M. Kaupp , J. Chem. Theory Comput. 2024, 20, 2033. 38411554 10.1021/acs.jctc.3c01422 PMC10938646

[59] F. Bruder , F. Weigend , Y. J. Franzke , J. Phys. Chem. A 2024, 128, 7298. 39163640 10.1021/acs.jpca.4c03794 PMC11372758

[60] F. Neese , Mol. Sci. 2022, 12, e1606.

[61] V. N. Staroverov , G. E. Scuseria , J. Tao , J. P. Perdew , J. Chem. Phys. 2003, 119, 12129.

[62] F. Neese , J. Comput. Chem. 2003, 24, 1740. 12964192 10.1002/jcc.10318

[63] G. L. Stoychev , A. A. Auer , F. Neese , J. Chem. Theory Comput. 2017, 13, 554. 28005364 10.1021/acs.jctc.6b01041

[64] C. R. Groom , I. J. Bruno , M. P. Lightfoot , S. C. Ward , Acta. Crystallogr. B 2016, 72, 171. 10.1107/S2052520616003954 PMC4822653 27048719

[65] J. P. Perdew , Phys. Rev. B Condens. Matter. 1986, 33, 8822. 9938299 10.1103/physrevb.33.8822

[66] A. D. Becke , Phys. Rev. A 1988, 38, 3098. 10.1103/physreva.38.3098 9900728

[67] C. Lee , W. Yang , R. G. Parr , Phys. Rev. B 1988, 37, 785. 10.1103/physrevb.37.785 9944570

[68] H. S. Yu , X. He , D. G. Truhlar , J. Chem. Theory Comput. 2016, 12, 1280. 26722866 10.1021/acs.jctc.5b01082

[69] J. Tao , J. P. Perdew , V. N. Staroverov , G. E. Scuseria , Phys. Rev. Lett. 2003, 91, 146401. 14611541 10.1103/PhysRevLett.91.146401

[70] Y. Zhao , D. G. Truhlar , J. Chem. Phys. 2006, 125, 194101. 17129083 10.1063/1.2370993

[71] J. W. Furness , A. D. Kaplan , J. Ning , J. P. Perdew , J. Sun , J. Phys. Chem. Lett. 2020, 11, 8208. 32876454 10.1021/acs.jpclett.0c02405

[72] A. D. Becke , J. Chem. Phys. 1993, 98, 5648.

[73] C. Adamo , V. Barone , J. Chem. Phys. 1999, 110, 6158.

[74] A. D. Becke , J. Chem. Phys. 1993, 98, 1372.

[75] J. P. Perdew , Y. Wang , Phys. Rev. B 1992, 45, 13244. 10.1103/physrevb.45.13244 10001404

[76] J. P. Perdew , Phys. B 1991, 172, 1, https://doi.10.1016/0921‐4526(91)90409‐8.

[77] Y. Zhao , N. E. Schultz , D. G. Truhlar , J. Chem. Phys. 2005, 123, 161103. 16268672 10.1063/1.2126975

[78] Y. Zhao , D. G. Truhlar , Theor. Chem. Acc. 2008, 120, 215.

[79] H. S. Yu , X. He , S. L. Li , D. G. Truhlar , Chem. Sci. 2016, 7, 5032. 30155154 10.1039/c6sc00705h PMC6018516

[80] J.‐D. Chai , M. Head‐Gordon , Phys. Chem. Chem. Phys. 2008, 10, 6615. 18989472 10.1039/b810189b

[81] T. Yanai , D. P. Tew , N. C. Handy , Chem. Phys. Lett. 2004, 393, 51.

[82] H. Iikura , T. Tsuneda , T. Yanai , K. Hirao , J. Chem. Phys. 2001, 115, 3540.

[83] S. Grimme , J. Chem. Phys. 2006, 124, 034108. 16438568 10.1063/1.2148954

[84] A. Karton , A. Tarnopolsky , J.‐F. Lamère , G. C. Schatz , J. M. L. Martin , J. Phys. Chem. A 2008, 112, 12868. 18714947 10.1021/jp801805p

[85] M. Casanova‐Páez , M. B. Dardis , L. Goerigk , J. Chem. Theory Comput. 2019, 15, 4735. 31298850 10.1021/acs.jctc.9b00013

[86] M. Casanova‐Páez , L. Goerigk , J. Chem. Theory Comput. 2021, 17, 5165. 34291643 10.1021/acs.jctc.1c00535

[87] L. Wittmann , H. Neugebauer , S. Grimme , M. Bursch , J. Chem. Phys. 2023, 159, 224103. 38063220 10.1063/5.0174988

[88] S. Lehtola , C. Steigemann , M. J. T. Oliveira , M. A. L. Marques , SoftwareX 2018, 7, 1, https://doi.10.1016/j.softx.2017.11.002.

[89] E. van Lenthe , E. J. Baerends , J. G. Snijders , J. Chem. Phys. 1993, 99, 4597.

[90] E. van Lenthe , E. J. Baerends , J. G. Snijders , J. Chem. Phys. 1994, 101, 9783.

[91] E. van Lenthe , J. G. Snijders , E. J. Baerends , J. Chem. Phys. 1996, 105, 6505.

[92] F. Weigend , R. Ahlrichs , Phys. Chem. Chem. Phys. 2005, 7, 3297. 16240044 10.1039/b508541a

[93] E. Malkin , I. Malkin , O. L. Malkina , V. G. Malkin , M. Kaupp , Phys. Chem. Chem. Phys. 2006, 8, 4079. 17028696 10.1039/b607044b

[94] L. Visscher , K. G. Dyall , Atom. Data Nucl. Data Tabl. 1997, 67, 207.

[95] F. Neese , J. Chem. Phys. 2005, 122, 034107. 10.1063/1.1829047 15740192

[96] D. Datta , S. Kossmann , F. Neese , J. Chem. Phys. 2016, 145, 114101.

[97] M. Witwicki , ChemPhysChem 2025, 26, e202400978. 40178176 10.1002/cphc.202400978

[98] S. Sproules , P. Banerjee , T. Weyhermüller , Y. Yan , J. P. Donahue , K. Wieghardt , Inorg. Chem. 2011, 50, 7106. 21699192 10.1021/ic2006265

[99] A. V. Astashkin , F. Neese , A. M. Raitsimring , J. J. A. Cooney , E. Bultman , J. H. Enemark , J. Am. Chem. Soc. 2005, 127, 16713. 16305262 10.1021/ja055472y

[100] N. Ueyama , H. Oku , M. Kondo , T.‐A. Okamura , N. Yoshinaga , A. Nakamura , Inorg. Chem. 1996, 35, 643.

[101] G. L. Wilson , R. J. Greenwood , J. R. Pilbrow , J. T. Spence , A. G. Wedd , J. Am. Chem. Soc. 1991, 113, 6803.

[102] G. Peng , J. Nichols , E. A. McCullough Jr. , J. T. Spence , Inorg. Chem. 1994, 33, 2857.

[103] I. K. Dhawan , J. H. Enemark , Inorg. Chem. 1996, 35, 4873. 11666687 10.1021/ic9605276

[104] K. K. Sunil , M. T. Rogers , Inorg. Chem. 1981, 20, 3283.

