# Supplementary material for: Accurate Calculation of Electron Paramagnetic Resonance Parameters for Molybdenum Compounds
Source: Chemphyschem. 2025 Oct 16;26(22):e202500317. doi: 10.1002/cphc.202500317 (PMC12640672; doi:10.1002/cphc.202500317)
Supplement: Supplementary file 1 — Supplementary Material [file CPHC-26-e202500317-s001.pdf]

Supplementary Information

for

Accurate calculation of electron paramagnetic resonance parameters for molybdenum compounds

Maria Drosou,[1,2]* Iris Wehrung,[3] Dimitrios A. Pantazis,[1]* Maylis Orio[3]*

[1] Max-Planck-Institut für Kohlenforschung, Kaiser-Wilhelm-Platz 1, 45470 Mülheim an der Ruhr, Germany.
[2] Department of Chemistry, Quantum Chemistry, TU Darmstadt, Peter-Grünberg-Str. 4, 64287, Darmstadt, Germany.
[3] Aix-Marseille Univ, CNRS, Centrale Med, iSm2, Marseille, France.

E-mail: drosou@kofo.mpg.de; dimitrios.pantazis@kofo.mpg.de; maylis.orio@univ-amu.fr

Table S1. Root mean square deviations (RMSDs) of structures obtained from geometry optimizations using the TPSSh functional from experimental structures for the crystallographically characterized complexes of the benchmark set. The RMSDs are obtained considering all heavy atoms (*i.e.* all atoms except hydrogen) and considering only those bound directly to Mo (1st coordination sphere).

	Complex	All heavy atoms	1st coord. sphere	CCDC ID	Ref.
**1**	$[Mo(bdt)_3]^-$	0.30	0.08	ACODAX	[1]
**3**	$[MoO(SPh)_4]^-$	0.44	0.05	PASMOT	[2]
**4**	$[MoO(bdt)_2]^-$	0.23	0.04	FACYEN	[3]
**5**	$(L3S)MoO(SPh)_2$	0.58	0.05	ALEGAZ	[4]
**6**	(L3S)MoO(bdt)	0.49	0.06	ALEFUS	[4]
**9**	(L1)MoO(SEt)	0.17	0.04	QEKJIZ	[5]
**15**	(LBH)MoO(bdt)	0.12	0.02	HERYIM10	[6]
**16**	(LBH)MoS(bdt)	0.10	0.05	QEKJIZ	[6]
**17**	(LBH)MoS(cat)	0.08	0.03	QIDZEJ	[6]
**19**	$[MoOCl_2(mdt)]^-$	0.03	0.03	XOCPUA	[7]
**20**	$[MoOCl_4]^-$	0.03	0.03	PASCMO	[8]

Table S2. Mo hyperfine tensor components and detailed contributions (MHz) of complex **1** obtained using the ZORA Hamiltonian and ZORA-def2-TZVP basis sets on the ligands.

Mo Basis Set	Total			Isotropic		Anisotropic					
	A_{11}	A_{22}	A_{33}	A^{FC}	A^{PC}	A_{11}^{SD}	A_{22}^{SD}	A_{33}^{SD}	$A_{11}^{SO,an}$	$A_{22}^{SO,an}$	$A_{33}^{SO,an}$
SARC-ZORA-TZVP	123.1	105.7	36.5	81.8	6.6	31.8	15.4	-47.2	-2.8	-1.9	4.7
SARC-ZORA-TZVP s-decontracted	121.9	104.6	35.4	80.7	6.6	31.8	15.4	-47.2	-2.8	-1.9	4.7
SARC-ZORA-TZVP s-decontracted +3s	124.6	107.4	38.0	83.4	6.6	31.8	15.5	-47.3	-2.8	-1.9	4.7

Table S3. Mo hyperfine tensor components and detailed contributions (MHz) of complex **1** obtained using the X2C Hamiltonian using the point and finite nucleus.

Mo Basis Set	Total			Isotropic		Anisotropic					
	A_{11}	A_{22}	A_{33}	A^{FC}	A^{PC}	A_{11}^{SD}	A_{22}^{SD}	A_{33}^{SD}	$A_{11}^{SO,an}$	$A_{22}^{SO,an}$	$A_{33}^{SO,an}$
SARC-DKH-TZVP											
point nucleus	124.5	107.2	38.3	83.3	6.7	31.7	15.4	-47.1	-2.9	-1.9	4.8
finite nucleus	124.4	107.1	38.2	83.2	6.7	31.7	15.4	-47.1	-2.9	-1.9	4.8
SARC-DKH-TZVP s-decontracted +3s											
point nucleus	124.4	107.2	38.2	83.2	6.7	31.7	15.4	-47.1	-2.9	-1.9	4.8
finite nucleus	123.9	106.7	37.7	82.7	6.7	31.7	15.4	-47.1	-2.9	-1.9	4.8

Table S4. g-tensor of complex **1** obtained using the X2C Hamiltonian.

Mo Basis Set	g_{11}	g_{22}	g_{33}	Δg_{11}	Δg_{22}	Δg_{33}
SARC-DKH-TZVP	2.002882	2.001692	1.985504	0.563	-0.627	-16.815
SARC-DKH-TZVPP	2.002327	2.001109	1.985613	0.008	-1.210	-16.706

Table S5. Core and valence-shell spin contributions to the isotropic HFCs (in MHz) for complex **1** obtained with different functionals.

**Functional**	**1s**	**2s**	**3s**	**4s**	**Core**	**Valence**	A^{FC}
PBE0-DH	1.5	53.4	-29.1	181.4	207.1	-123.9	83.2
B3PW91 40%	1.6	45.6	-23.7	153.4	176.9	-91.9	84.9
B3PW91 30%	1.3	40.1	-20.7	137.9	158.7	-82.9	75.8
B3PW91 50%	1.8	51.2	-26.8	168.6	194.9	-101.2	93.7
CAM-B3LYP	1.2	38.0	-19.7	117.6	137.2	-64.7	72.5
ωB97	0.1	41.9	-45.8	171.2	167.4	-68.2	99.2
r^2SCAN	-2.2	47.7	-42.8	163.6	166.3	-93.3	73.0
M05	-3.4	60.8	-47.4	129.9	139.9	-34.2	105.7
B3PW91 20%	1.1	34.8	-17.8	122.3	140.4	-74.1	66.2
BLYP	0.7	25.3	-14.3	85.4	97.0	-46.5	50.5
M06	0.0	36.4	-22.3	65.2	79.4	-50.1	29.3

Complete results for ^{95}Mo hyperfine coupling constants

Table S6. Individual components and detailed contributions (MHz) of the hyperfine coupling constants for the Mo-based complexes obtained using the BLYP functional.

	A_{11}	A_{22}	A_{33}	A^{FC}	A^{PC}	A_{11}^{SD}	A_{22}^{SD}	A_{33}^{SD}	$A_{11}^{SOC,an}$	$A_{22}^{SOC,an}$	$A_{33}^{SOC,an}$
**1**	81.8	71.6	10.0	50.5	4.0	25.3	15.6	-40.9	2.1	1.6	-3.6
**2**	78.6	76.9	10.0	51.2	4.0	21.7	20.0	-41.7	1.8	1.8	-3.7
**3**	101.8	31.7	31.7	51.0	4.1	45.4	-22.7	-22.7	1.5	-0.8	-0.8
**5**	108.9	39.7	35.6	56.6	4.9	45.9	-21.3	-24.6	1.8	-0.5	-1.2
**6**	108.5	37.6	34.1	55.9	4.2	46.3	-21.7	-24.6	2.3	-0.9	-1.4
**7**	110.9	41.3	37.6	53.4	9.9	41.3	-20.9	-20.4	6.4	-1.1	-5.3
**8**	115.8	40.5	37.5	59.5	5.2	49.9	-23.7	-26.3	1.4	-0.5	-0.9
**9**	110.7	39.8	34.5	56.6	5.2	48.1	-21.8	-26.3	1.1	-0.1	-1.0
**10**	143.7	52.2	45.9	68.5	12.1	49.2	-24.7	-24.5	14.1	-3.8	-10.3
**11**	135.1	51.3	46.1	69.9	7.7	54.3	-25.0	-29.3	3.4	-1.2	-2.2
**14**	136.2	47.8	43.4	69.8	6.0	57.4	-27.1	-30.3	3.2	-1.0	-2.2
**15**	122.9	45.9	39.0	64.0	5.3	49.9	-22.1	-27.7	3.9	-1.3	-2.6
**16**	125.3	48.7	41.2	65.5	6.3	49.1	-21.4	-27.7	4.6	-1.6	-3.0
**17**	144.1	51.3	44.1	70.3	9.7	58.6	-27.7	-30.9	5.9	-1.0	-4.9
**18**	132.2	33.5	29.0	56.5	8.4	61.4	-29.8	-31.5	6.1	-1.7	-4.5
**19**	133.6	46.0	44.1	68.4	6.2	56.5	-27.3	-29.1	2.8	-1.3	-1.5
**20**	156.5	59.3	59.3	82.6	9.1	61.9	-31.0	-31.0	3.1	-1.6	-1.6
**21**	153.8	61.2	60.8	83.5	8.5	57.9	-28.6	-29.2	4.2	-2.2	-2.0
**22**	155.2	64.5	60.8	83.1	10.5	58.4	-28.2	-30.2	3.4	-0.9	-2.5

Table S7. Individual components and detailed contributions (MHz) of the hyperfine coupling constants for the Mo-based complexes obtained using the BP86 functional.

	A_{11}	A_{22}	A_{33}	A^{FC}	A^{PC}	A_{11}^{SD}	A_{22}^{SD}	A_{33}^{SD}	$A_{11}^{SOC,an}$	$A_{22}^{SOC,an}$	$A_{33}^{SOC,an}$
**1**	78.9	69.1	10.0	48.8	3.9	24.4	15.0	-39.3	1.9	1.5	-3.4
**2**	76.1	74.4	10.0	49.6	3.9	20.9	19.3	-40.1	1.7	1.7	-3.5
**3**	98.1	29.1	29.1	48.0	4.1	44.7	-22.4	-22.3	1.4	-0.7	-0.7
**5**	105.8	38.0	33.9	54.4	4.8	45.0	-20.8	-24.2	1.7	-0.5	-1.2
**6**	105.2	35.2	32.0	53.3	4.2	45.7	-21.5	-24.2	2.2	-0.9	-1.4
**7**	107.3	39.6	36.3	51.5	9.6	40.2	-20.4	-19.8	6.2	-1.1	-5.1
**8**	111.9	38.1	35.1	56.6	5.2	48.9	-23.1	-25.8	1.4	-0.5	-0.9
**9**	106.7	37.6	32.3	53.8	5.1	46.9	-21.2	-25.7	1.1	-0.1	-1.0
**10**	138.8	49.8	44.0	65.6	11.9	47.7	-24.1	-23.6	13.8	-3.7	-10.0
**11**	131.5	49.7	44.4	67.7	7.6	53.1	-24.4	-28.8	3.3	-1.2	-2.1
**14**	131.7	45.7	41.5	67.0	5.9	55.8	-26.4	-29.4	3.1	-1.0	-2.2
**15**	119.5	43.6	37.3	61.5	5.3	49.0	-22.0	-27.1	3.8	-1.3	-2.5
**16**	121.8	47.1	40.4	63.6	6.2	47.7	-21.1	-26.6	4.5	-1.7	-2.8
**17**	139.8	50.9	44.0	68.8	9.4	56.1	-26.6	-29.5	5.7	-0.9	-4.8
**18**	127.6	32.1	28.3	54.5	8.2	59.2	-29.1	-30.1	5.9	-1.6	-4.3
**19**	128.5	43.5	41.4	65.0	6.1	54.9	-26.5	-28.4	2.6	-1.2	-1.4

**20**	152.9	57.9	57.9	80.7	8.9	60.5	-30.3	-30.3	3.0	-1.5	-1.5
**21**	151.2	61.1	60.6	82.6	8.4	56.3	-27.9	-28.5	4.1	-2.1	-2.0
**22**	153.1	64.5	61.0	82.7	10.2	57.0	-27.6	-29.4	3.4	-0.9	-2.5

Table S8. Individual components and detailed contributions (MHz) of the hyperfine coupling constants for the Mo-based complexes obtained using the MN15-L functional.

	A_{11}	A_{22}	A_{33}	A^{FC}	A^{PC}	A_{11}^{SD}	A_{22}^{SD}	A_{33}^{SD}	$A_{11}^{SOC,an}$	$A_{22}^{SOC,an}$	$A_{33}^{SOC,an}$
**1**	60.0	50.4	-5.2	28.4	6.5	22.9	13.8	-36.7	2.1	1.6	-3.7
**2**	58.4	56.7	-6.3	29.7	6.4	20.3	18.7	-39.0	1.9	1.8	-3.7
**3**	78.0	1.5	1.5	21.2	5.6	49.1	-24.5	-24.5	2.1	-1.0	-1.1
**5**	85.1	11.2	6.6	27.7	6.5	48.4	-22.1	-26.3	2.6	-1.1	-1.5
**6**	80.3	2.6	-1.0	20.8	6.3	50.1	-23.4	-26.7	3.1	-1.4	-1.7
**7**	88.2	15.8	14.7	30.2	9.3	43.0	-19.9	-23.2	5.7	-3.9	-1.8
**8**	86.1	8.1	4.5	25.9	6.9	51.0	-23.9	-27.2	2.3	-1.0	-1.3
**9**	81.6	7.6	3.4	24.1	6.7	49.0	-22.8	-26.2	1.9	-0.6	-1.3
**10**	112.7	19.5	19.3	38.4	12.0	49.4	-27.1	-22.4	12.9	-4.0	-8.9
**11**	106.9	23.1	17.0	40.1	8.7	54.0	-24.3	-29.7	4.1	-1.6	-2.4
**14**	99.5	13.5	9.7	32.9	7.8	54.8	-26.1	-28.6	4.0	-1.3	-2.6
**15**	89.1	8.0	3.8	26.1	7.4	51.1	-23.8	-27.3	4.5	-1.9	-2.5
**16**	86.4	14.2	10.8	28.3	8.6	44.7	-20.9	-23.8	4.7	-2.1	-2.6
**17**	107.7	29.6	25.1	43.1	10.8	47.8	-23.2	-24.6	5.9	-1.5	-4.4
**18**	104.9	12.9	12.4	33.7	9.5	55.2	-28.5	-26.7	6.4	-2.1	-4.4
**19**	108.9	21.5	19.5	42.1	7.7	55.5	-26.8	-28.8	3.6	-1.8	-1.8
**20**	142.2	44.4	44.4	67.1	9.7	61.1	-30.6	-30.6	4.3	-2.1	-2.1
**21**	137.3	45.9	45.7	66.4	9.7	56.0	-27.9	-28.1	5.2	-2.6	-2.6
**22**	143.4	51.6	49.2	70.3	10.9	57.4	-28.0	-29.4	4.8	-1.9	-2.9

Table S9. Individual components and detailed contributions (MHz) of the hyperfine coupling constants for the Mo-based complexes obtained using the r^2SCAN functional.

	A_{11}	A_{22}	A_{33}	A^{FC}	A^{PC}	A_{11}^{SD}	A_{22}^{SD}	A_{33}^{SD}	$A_{11}^{SOC,an}$	$A_{22}^{SOC,an}$	$A_{33}^{SOC,an}$
**1**	102.2	92.4	35.7	73.0	3.7	23.7	14.4	-38.1	1.7	1.3	-3.1
**2**	98.7	97.0	34.0	72.8	3.7	20.6	19.0	-39.6	1.5	1.5	-3.0
**3**	117.9	46.6	46.6	66.7	3.7	46.3	-23.1	-23.1	1.4	-0.7	-0.7
**5**	126.3	56.5	53.3	74.3	4.3	46.0	-21.7	-24.3	1.7	-0.6	-1.1
**6**	128.6	55.6	52.6	74.9	4.0	47.6	-22.4	-25.1	2.2	-0.9	-1.3
**7**	120.8	53.3	51.3	67.6	7.5	41.0	-20.8	-20.2	4.8	-1.1	-3.7
**8**	131.3	57.4	54.5	76.4	4.6	49.0	-23.1	-25.8	1.4	-0.6	-0.9
**9**	124.5	55.1	50.1	72.1	4.5	46.9	-21.4	-25.6	1.1	-0.2	-1.0
**10**	158.0	71.2	66.7	88.9	9.7	48.3	-24.4	-23.9	11.2	-3.1	-8.1
**11**	153.1	71.8	66.7	90.7	6.5	52.9	-24.2	-28.7	3.2	-1.2	-1.9
**14**	159.4	73.9	72.3	96.4	5.3	54.6	-27.1	-27.6	3.1	-1.0	-2.1

**15**	144.0	66.2	61.2	85.5	4.9	50.1	-22.9	-27.2	3.6	-1.4	-2.1
**16**	148.0	74.2	69.2	91.4	5.6	47.0	-21.3	-25.7	4.0	-1.7	-2.3
**17**	170.2	84.2	81.5	103.7	8.2	53.2	-26.7	-26.5	5.2	-1.1	-4.0
**18**	158.9	65.2	64.0	88.7	7.3	57.5	-27.1	-30.4	5.5	-3.8	-1.7
**19**	151.8	68.7	66.1	90.1	5.4	53.9	-25.7	-28.2	2.6	-1.2	-1.3
**20**	178.4	84.5	84.5	108.3	7.5	59.8	-29.9	-29.9	3.0	-1.5	-1.5
**21**	178.0	89.4	88.0	111.0	7.4	55.8	-27.2	-28.6	3.9	-1.9	-2.0
**22**	179.5	90.1	88.3	110.6	8.7	56.8	-27.9	-28.9	3.6	-1.4	-2.2

Table S10. Individual components and detailed contributions (MHz) of the hyperfine coupling constants for the Mo-based complexes obtained using the M06-L functional.

	A_{11}	A_{22}	A_{33}	A^{FC}	A^{PC}	A_{11}^{SD}	A_{22}^{SD}	A_{33}^{SD}	$A_{11}^{SOC,an}$	$A_{22}^{SOC,an}$	$A_{33}^{SOC,an}$
**1**	117.1	107.0	49.7	87.5	3.7	24.1	14.3	-38.4	1.8	1.4	-3.3
**2**	112.7	110.9	47.3	86.6	3.7	20.8	19.0	-39.8	1.7	1.6	-3.3
**3**	128.2	55.4	55.4	75.7	3.9	47.2	-23.6	-23.6	1.5	-0.8	-0.8
**5**	129.9	58.6	55.7	76.7	4.6	46.8	-22.2	-24.6	1.9	-0.7	-1.2
**6**	134.9	60.5	57.7	80.3	4.1	48.4	-23.0	-25.4	2.3	-1.0	-1.3
**7**	116.7	47.4	45.6	61.4	8.4	41.4	-21.1	-20.3	5.5	-1.5	-4.0
**8**	137.7	62.3	59.4	81.6	4.8	49.8	-23.6	-26.1	1.6	-0.6	-0.9
**9**	130.9	60.0	55.2	77.2	4.7	47.8	-21.9	-25.9	1.3	-0.3	-1.0
**10**	145.7	57.4	53.3	75.1	10.4	48.6	-24.8	-23.8	11.7	-3.4	-8.3
**11**	147.8	66.1	60.9	84.6	6.9	53.0	-24.2	-28.7	3.4	-1.3	-2.0
**14**	158.4	72.8	70.5	95.1	5.4	54.9	-26.9	-28.0	3.2	-1.0	-2.1
**15**	145.3	66.6	61.8	86.1	5.1	50.6	-23.2	-27.3	3.7	-1.5	-2.2
**16**	155.8	81.6	76.4	98.8	5.7	47.3	-21.4	-25.9	4.0	-1.7	-2.4
**17**	161.8	76.5	73.0	95.2	8.6	52.8	-26.3	-26.6	5.3	-1.1	-4.2
**18**	142.9	48.8	48.4	72.3	7.7	57.3	-27.3	-30.0	5.7	-4.0	-1.7
**19**	158.1	73.4	70.6	95.0	5.6	54.7	-26.0	-28.7	2.9	-1.4	-1.5
**20**	171.5	77.0	77.0	100.3	8.1	59.7	-29.8	-29.8	3.5	-1.8	-1.8
**21**	168.4	78.7	77.9	100.4	7.9	55.8	-27.5	-28.3	4.3	-2.1	-2.2
**22**	163.0	73.5	71.6	93.3	9.4	56.6	-27.8	-28.7	3.9	-1.5	-2.4

Table S11. Individual components and detailed contributions (MHz) of the hyperfine coupling constants for the Mo-based complexes obtained using the TPSS functional.

	A_{11}	A_{22}	A_{33}	A^{FC}	A^{PC}	A_{11}^{SD}	A_{22}^{SD}	A_{33}^{SD}	$A_{11}^{SOC,an}$	$A_{22}^{SOC,an}$	$A_{33}^{SOC,an}$
**1**	93.4	83.5	24.5	63.3	3.8	24.5	15.0	-39.5	1.8	1.4	-3.3
**2**	89.9	88.2	23.9	63.5	3.8	21.0	19.3	-40.3	1.6	1.6	-3.3
**3**	113.1	42.7	42.7	62.2	3.9	45.7	-22.8	-22.8	1.4	-0.7	-0.7
**5**	121.0	52.3	48.6	69.4	4.6	45.5	-21.2	-24.3	1.7	-0.5	-1.1
**6**	121.8	50.6	47.4	69.2	4.0	46.5	-21.9	-24.7	2.1	-0.8	-1.3
**7**	118.9	51.7	48.4	64.4	8.6	40.7	-20.4	-20.3	5.3	-1.0	-4.3

**8**	127.7	53.8	50.7	72.5	4.9	49.1	-23.2	-25.9	1.4	-0.5	-0.8
**9**	121.8	52.1	47.0	68.8	4.9	47.2	-21.4	-25.8	1.1	-0.1	-0.9
**10**	154.6	67.0	60.9	83.4	10.8	48.3	-23.9	-24.3	12.3	-3.3	-9.0
**11**	147.9	66.2	60.9	84.7	7.0	53.3	-24.4	-28.9	3.2	-1.2	-2.0
**14**	151.1	65.3	61.7	87.1	5.6	55.6	-26.6	-29.0	3.0	-0.9	-2.1
**15**	137.0	60.5	54.5	79.0	5.0	49.5	-22.3	-27.3	3.6	-1.3	-2.3
**16**	139.1	64.5	58.0	81.4	5.8	47.9	-21.2	-26.7	4.1	-1.5	-2.6
**17**	159.1	70.2	64.5	89.3	8.7	56.0	-26.9	-29.1	5.3	-0.9	-4.4
**18**	147.5	52.0	49.4	75.3	7.7	59.2	-29.6	-29.6	5.6	-1.5	-4.0
**19**	146.0	60.9	58.7	82.7	5.8	55.2	-26.5	-28.6	2.5	-1.2	-1.3
**20**	170.7	75.6	75.6	99.1	8.2	60.8	-30.4	-30.4	2.9	-1.4	-1.4
**21**	169.1	79.3	78.4	101.1	7.8	56.5	-27.8	-28.7	3.9	-1.9	-1.9
**22**	170.8	81.6	78.6	100.9	9.4	57.4	-27.9	-29.5	3.3	-1.0	-2.3

Table S12. Individual components and detailed contributions (MHz) of the hyperfine coupling constants for the Mo-based complexes obtained using the B3PW91 functional with 20% HFX.

	A_{11}	A_{22}	A_{33}	A^{FC}	A^{PC}	A_{11}^{SD}	A_{22}^{SD}	A_{33}^{SD}	$A_{11}^{SOC,an}$	$A_{22}^{SOC,an}$	$A_{33}^{SOC,an}$
**1**	101.2	90.3	23.0	66.2	5.3	27.4	17.0	-44.4	2.4	1.9	-4.3
**2**	97.0	95.2	21.1	65.9	5.2	23.9	22.0	-45.9	2.1	2.1	-4.2
**3**	123.7	43.5	43.5	65.3	4.9	51.9	-26.0	-25.9	1.7	-0.9	-0.9
**5**	132.5	54.5	50.6	73.3	5.9	51.3	-24.0	-27.2	2.2	-0.7	-1.5
**6**	133.5	50.9	48.4	72.3	5.3	53.2	-25.6	-27.6	2.8	-1.2	-1.6
**7**	128.9	55.4	52.2	68.4	10.5	43.2	-21.9	-21.3	7.0	-1.6	-5.4
**8**	136.5	54.6	51.2	74.6	6.2	54.0	-25.5	-28.5	1.9	-0.7	-1.2
**9**	130.8	53.2	48.0	71.3	6.0	52.1	-23.9	-28.2	1.5	-0.3	-1.2
**10**	173.8	73.7	64.1	89.6	14.2	52.9	-25.6	-27.3	17.2	-4.6	-12.6
**11**	157.0	67.6	61.8	87.0	8.5	57.7	-26.4	-31.3	4.1	-1.6	-2.5
**14**	157.2	63.6	59.8	86.6	6.9	60.0	-28.8	-31.2	3.8	-1.2	-2.6
**15**	147.1	59.4	55.2	80.8	6.4	55.6	-26.1	-29.5	4.4	-1.8	-2.6
**16**	149.0	65.2	61.6	84.3	0.0	52.4	-24.8	-27.6	12.5	5.6	4.8
**17**	167.3	75.0	67.4	92.6	10.6	58.2	-27.3	-30.8	6.1	-1.0	-5.1
**18**	157.1	55.2	52.1	79.0	9.1	62.5	-31.2	-31.4	6.7	-1.9	-4.8
**19**	152.3	60.0	58.0	83.2	6.9	59.1	-28.6	-30.5	3.3	-1.6	-1.7
**20**	176.8	75.0	75.0	99.3	9.6	64.3	-32.2	-32.2	3.8	-1.9	-1.9
**21**	177.9	80.7	79.6	103.3	9.4	60.2	-29.5	-30.7	5.1	-2.6	-2.5
**22**	180.1	82.9	80.2	103.2	0.0	61.5	-30.0	-31.5	15.7	9.6	8.4

Table S13. Individual components and detailed contributions (MHz) of the hyperfine coupling constants for the Mo-based complexes obtained using the B3PW91 functional with 30% HFX.

	A_{11}	A_{22}	A_{33}	A^{FC}	A^{PC}	A_{11}^{SD}	A_{22}^{SD}	A_{33}^{SD}	$A_{11}^{SOC,an}$	$A_{22}^{SOC,an}$	$A_{33}^{SOC,an}$
**1**	112.7	101.5	31.3	75.8	6.1	28.4	17.7	-46.1	2.5	2.1	-4.6

**2**	107.7	105.8	28.1	74.6	5.9	25.0	23.1	-48.0	2.3	2.3	-4.6
**3**	137.0	52.0	52.0	75.0	5.3	54.9	-27.5	-27.5	1.9	-0.9	-1.0
**5**	146.5	64.2	60.1	83.9	6.3	54.0	-25.4	-28.6	2.4	-0.8	-1.6
**6**	147.3	60.2	57.8	82.6	5.8	56.0	-27.0	-29.0	3.1	-1.3	-1.8
**7**	139.6	64.0	60.8	77.4	10.8	44.1	-22.4	-21.7	7.5	-1.8	-5.6
**8**	149.5	64.0	60.4	84.7	6.6	56.2	-26.5	-29.7	2.2	-0.9	-1.4
**9**	143.6	62.4	57.2	81.2	6.5	54.3	-24.9	-29.3	1.8	-0.4	-1.3
**10**	190.5	86.5	75.0	102.2	15.2	54.6	-25.9	-28.7	18.7	-5.1	-13.7
**11**	170.4	77.9	71.8	97.8	8.9	59.4	-27.2	-32.3	4.5	-1.7	-2.7
**14**	170.1	73.9	70.4	97.4	7.4	61.3	-29.6	-31.7	4.2	-1.3	-2.9
**15**	160.7	68.7	65.4	91.3	6.9	57.9	-27.6	-30.3	4.7	-2.0	-2.7
**16**	163.0	76.7	75.1	96.6	8.3	53.4	-26.2	-27.2	4.9	-2.2	-2.7
**17**	182.1	90.1	81.5	106.7	11.2	58.2	-26.8	-31.4	6.1	-1.0	-5.1
**18**	172.1	67.9	65.3	92.3	9.5	63.5	-31.8	-31.7	7.0	-2.1	-4.9
**19**	164.8	69.6	67.6	93.4	7.3	60.7	-29.4	-31.4	3.6	-1.8	-1.8
**20**	189.8	85.1	85.1	110.1	9.9	65.8	-32.9	-32.9	4.2	-2.1	-2.1
**21**	192.3	92.3	91.0	115.3	9.9	61.6	-30.1	-31.5	5.7	-2.9	-2.8
**22**	194.4	93.8	91.5	115.0	11.6	63.0	-30.8	-32.2	5.0	-2.0	-3.0

Table S14. Individual components and detailed contributions (MHz) of the hyperfine coupling constants for the Mo-based complexes obtained using the B3PW91 functional with 40% HFX.

	A_{11}	A_{22}	A_{33}	A^{FC}	A^{PC}	A_{11}^{SD}	A_{22}^{SD}	A_{33}^{SD}	$A_{11}^{SOC,an}$	$A_{22}^{SOC,an}$	$A_{33}^{SOC,an}$
**1**	123.7	112.3	39.7	84.9	7.0	29.2	18.2	-47.5	2.6	2.2	-4.8
**2**	117.9	115.9	35.0	82.9	6.7	25.9	24.0	-49.9	2.4	2.4	-4.8
**3**	150.1	60.7	60.7	84.7	5.7	57.7	-28.8	-28.8	2.1	-1.1	-1.1
**5**	160.3	74.0	69.8	94.5	6.8	56.4	-26.5	-29.8	2.8	-0.9	-1.8
**6**	160.4	69.6	67.1	92.7	6.3	58.2	-28.1	-30.0	3.4	-1.4	-2.0
**7**	149.9	72.6	69.4	86.2	11.0	44.8	-22.7	-22.1	8.0	-2.1	-5.9
**8**	162.4	73.7	69.7	94.9	7.1	58.1	-27.3	-30.8	2.6	-1.0	-1.6
**9**	156.4	71.8	66.4	91.3	6.9	56.3	-25.9	-30.4	2.1	-0.6	-1.5
**10**	206.8	99.2	85.8	114.5	16.1	56.1	-26.0	-30.1	20.3	-5.5	-14.8
**11**	183.7	88.3	81.8	108.6	9.3	61.0	-27.8	-33.3	4.9	-1.9	-3.0
**14**	182.6	84.3	81.0	108.2	7.7	62.2	-30.3	-32.0	4.6	-1.5	-3.1
**15**	173.5	78.2	75.7	101.8	7.4	59.6	-28.8	-30.8	5.0	-2.3	-2.7
**16**	177.3	90.4	89.5	109.9	9.1	53.7	-26.3	-27.4	4.7	-2.5	-2.2
**17**	197.3	107.2	96.6	121.9	11.8	57.8	-25.7	-32.1	6.0	-1.0	-5.0
**18**	186.6	80.6	78.2	105.3	9.8	64.4	-32.4	-32.0	7.3	-2.3	-5.0
**19**	177.2	79.3	77.2	103.6	7.6	62.3	-30.1	-32.2	3.9	-2.0	-1.9
**20**	202.8	95.3	95.3	121.0	10.2	67.2	-33.6	-33.6	4.7	-2.3	-2.3
**21**	206.6	104.2	102.7	127.5	10.3	62.7	-30.5	-32.2	6.2	-3.2	-3.1
**22**	208.5	104.9	102.9	126.9	11.9	64.4	-31.5	-32.9	5.6	-2.5	-3.1

Table S15. Individual components and detailed contributions (MHz) of the hyperfine coupling constants for the Mo-based complexes obtained using the B3PW91 functional with 50% HFX.

	A_{11}	A_{22}	A_{33}	A^{FC}	A^{PC}	A_{11}^{SD}	A_{22}^{SD}	A_{33}^{SD}	$A_{11}^{SOC,an}$	$A_{22}^{SOC,an}$	$A_{33}^{SOC,an}$
**1**	134.2	122.6	48.1	93.7	7.9	30.0	18.7	-48.7	2.6	2.3	-5.0
**2**	127.4	125.4	41.6	90.6	7.5	26.8	24.8	-51.6	2.5	2.5	-5.0
**3**	162.7	69.4	69.4	94.3	6.1	60.1	-30.1	-30.0	2.4	-1.2	-1.2
**5**	173.6	83.8	79.3	104.9	7.3	58.5	-27.5	-31.0	3.1	-1.1	-2.0
**6**	172.9	79.1	76.4	102.7	6.7	59.9	-29.0	-31.0	3.7	-1.5	-2.2
**7**	159.9	81.0	77.7	94.9	11.3	45.4	-23.0	-22.4	8.5	-2.3	-6.2
**8**	175.2	83.4	79.1	105.0	7.5	59.8	-28.1	-31.8	3.0	-1.2	-1.8
**9**	169.1	81.3	75.8	101.3	7.4	58.1	-26.7	-31.4	2.5	-0.8	-1.7
**10**	222.9	111.9	96.6	126.8	16.9	57.4	-26.1	-31.3	21.8	-5.9	-16.0
**11**	196.7	98.9	91.8	119.4	9.7	62.4	-28.2	-34.2	5.4	-2.1	-3.2
**14**	195.0	95.0	91.9	119.1	8.1	63.0	-30.8	-32.2	4.9	-1.7	-3.3
**15**	186.0	87.9	86.1	112.1	7.8	61.0	-29.7	-31.3	5.2	-2.5	-2.7
**16**	192.3	108.6	104.0	124.9	10.0	53.1	-24.3	-28.8	4.4	-2.2	-2.2
**17**	213.8	127.4	113.4	139.0	12.5	56.8	-23.4	-33.4	5.7	-0.8	-4.8
**18**	200.7	93.1	90.9	118.1	10.1	65.1	-32.9	-32.2	7.6	-2.4	-5.1
**19**	189.6	88.9	86.8	113.8	8.0	63.7	-30.8	-32.9	4.2	-2.1	-2.1
**20**	215.9	105.6	105.6	131.9	10.4	68.6	-34.3	-34.3	5.1	-2.6	-2.6
**21**	221.1	116.4	114.7	140.0	10.7	63.7	-31.0	-32.7	6.9	-3.5	-3.4
**22**	222.5	116.2	114.3	138.8	12.2	65.6	-32.0	-33.6	6.2	-2.9	-3.2

Table S16. Individual components and detailed contributions (MHz) of the hyperfine coupling constants for the Mo-based complexes obtained using the PBE0 functional.

	A_{11}	A_{22}	A_{33}	A^{FC}	A^{PC}	A_{11}^{SD}	A_{22}^{SD}	A_{33}^{SD}	$A_{11}^{SOC,an}$	$A_{22}^{SOC,an}$	$A_{33}^{SOC,an}$
**1**	109.7	98.8	31.1	74.3	5.6	27.5	17.1	-44.6	2.4	1.9	-4.3
**2**	105.2	103.3	28.5	73.5	5.4	24.1	22.2	-46.4	2.1	2.1	-4.3
**3**	133.3	51.3	51.3	73.6	5.0	53.0	-26.5	-26.5	1.8	-0.9	-0.9
**5**	142.5	62.9	59.0	82.2	6.0	52.2	-24.5	-27.7	2.3	-0.8	-1.5
**6**	143.4	59.2	56.8	81.1	5.4	54.2	-26.1	-28.1	2.9	-1.2	-1.7
**7**	136.6	62.6	59.5	75.8	10.4	43.4	-22.0	-21.4	7.1	-1.7	-5.4
**8**	146.1	63.1	59.6	83.3	6.2	54.7	-25.8	-28.9	2.0	-0.8	-1.2
**9**	140.0	61.3	56.1	79.7	6.1	52.7	-24.2	-28.5	1.6	-0.4	-1.2
**10**	184.5	83.5	73.4	99.4	14.4	53.2	-25.6	-27.6	17.6	-4.8	-12.9
**11**	167.0	76.8	70.9	96.4	8.5	58.1	-26.5	-31.5	4.2	-1.6	-2.6
**14**	167.5	73.6	70.0	96.7	7.0	60.0	-29.0	-31.1	4.0	-1.3	-2.7
**15**	157.2	68.1	64.5	90.0	6.5	56.3	-26.7	-29.6	4.5	-1.9	-2.6
**16**	159.5	75.3	72.9	94.7	7.8	52.3	-25.2	-27.1	4.8	-2.1	-2.7
**17**	178.6	87.7	79.9	104.8	10.6	57.4	-26.8	-30.6	6.0	-1.0	-5.0
**18**	168.5	66.5	64.0	90.6	9.1	62.3	-31.3	-31.0	6.7	-2.0	-4.7
**19**	162.1	69.3	67.3	92.6	6.9	59.3	-28.7	-30.7	3.4	-1.7	-1.7

**20**	187.3	84.9	84.9	109.5	9.5	64.5	-32.3	-32.3	3.9	-2.0	-2.0
**21**	188.9	91.3	90.0	113.9	9.4	60.4	-29.5	-30.9	5.3	-2.7	-2.6
**22**	191.0	92.9	90.5	113.6	11.1	61.7	-30.2	-31.5	4.7	-1.8	-2.9

Table S17. Individual components and detailed contributions (MHz) of the hyperfine coupling constants for the Mo-based complexes obtained using the TPSSh functional.

	A_{11}	A_{22}	A_{33}	A^{FC}	A^{PC}	A_{11}^{SD}	A_{22}^{SD}	A_{33}^{SD}	$A_{11}^{SOC,an}$	$A_{22}^{SOC,an}$	$A_{33}^{SOC,an}$
**1**	105.2	94.9	32.0	72.9	4.4	25.9	16.0	-41.9	2.0	1.6	-3.6
**2**	101.1	99.4	30.4	72.6	4.4	22.4	20.7	-43.1	1.8	1.8	-3.6
**3**	126.2	50.5	50.6	71.5	4.3	49.1	-24.6	-24.5	1.5	-0.7	-0.8
**5**	134.7	61.1	57.5	79.4	5.0	48.5	-22.8	-25.8	1.9	-0.6	-1.2
**6**	136.5	59.2	56.5	79.5	4.5	50.2	-23.9	-26.3	2.4	-1.0	-1.4
**7**	129.6	59.8	56.5	73.1	8.9	42.1	-21.1	-21.0	5.7	-1.2	-4.5
**8**	140.2	62.4	59.1	81.9	5.3	51.5	-24.3	-27.2	1.6	-0.6	-1.0
**9**	133.9	60.3	55.3	77.9	5.3	49.6	-22.7	-27.0	1.2	-0.2	-1.0
**10**	171.4	79.0	71.2	95.5	11.7	50.8	-24.7	-26.1	13.7	-3.6	-10.0
**11**	160.9	75.6	70.1	94.8	7.4	55.4	-25.4	-30.0	3.5	-1.3	-2.2
**14**	164.2	74.8	71.4	97.4	6.0	57.6	-27.7	-29.9	3.3	-1.0	-2.3
**15**	151.4	69.2	64.2	89.4	5.5	52.8	-24.3	-28.5	3.9	-1.5	-2.4
**16**	153.4	74.3	69.3	92.5	6.5	50.2	-23.1	-27.2	4.4	-1.7	-2.6
**17**	173.2	82.9	76.9	101.8	9.2	56.9	-27.2	-29.7	5.5	-1.0	-4.5
**18**	162.6	64.2	61.9	88.2	8.0	60.6	-30.5	-30.2	5.9	-1.7	-4.2
**19**	158.0	69.6	67.4	92.2	6.1	57.1	-27.5	-29.6	2.8	-1.3	-1.4
**20**	183.0	84.8	84.8	109.0	8.5	62.5	-31.2	-31.2	3.2	-1.6	-1.6
**21**	182.8	89.7	88.6	112.1	8.2	58.3	-28.6	-29.7	4.3	-2.2	-2.1
**22**	184.8	91.6	88.9	112.0	9.8	59.5	-29.0	-30.5	3.8	-1.3	-2.4

Table S18. Individual components and detailed contributions (MHz) of the hyperfine coupling constants for the Mo-based complexes obtained using the B3LYP functional.

	A_{11}	A_{22}	A_{33}	A^{FC}	A^{PC}	A_{11}^{SD}	A_{22}^{SD}	A_{33}^{SD}	$A_{11}^{SOC,an}$	$A_{22}^{SOC,an}$	$A_{33}^{SOC,an}$
**1**	104.4	93.1	23.3	68.3	5.4	28.4	17.6	-45.9	2.5	2.0	-4.5
**2**	99.8	97.9	21.4	67.8	5.2	24.6	22.7	-47.3	2.2	2.2	-4.5
**3**	127.1	46.0	46.0	68.2	4.9	52.4	-26.2	-26.2	1.8	-0.9	-0.9
**5**	135.4	56.3	52.4	75.5	5.9	52.0	-24.4	-27.6	2.2	-0.7	-1.5
**6**	137.2	53.4	50.9	75.3	5.3	54.0	-26.0	-28.0	2.8	-1.2	-1.7
**7**	131.8	57.1	53.3	70.1	10.7	43.9	-22.1	-21.8	7.2	-1.6	-5.6
**8**	140.0	56.8	53.5	77.3	6.2	54.8	-26.0	-28.9	1.9	-0.7	-1.2
**9**	134.3	55.4	50.2	73.9	6.1	53.0	-24.3	-28.6	1.5	-0.3	-1.2
**10**	178.0	76.0	65.9	92.2	14.4	54.1	-26.1	-28.0	17.4	-4.7	-12.8
**11**	160.3	69.3	63.6	89.2	8.6	58.6	-27.0	-31.7	4.1	-1.6	-2.5
**14**	161.4	65.8	61.8	89.4	7.0	61.4	-29.4	-32.0	3.9	-1.3	-2.7

**15**	150.8	61.8	57.1	83.5	6.4	56.6	-26.4	-30.1	4.5	-1.8	-2.8
**16**	152.8	66.8	62.5	86.4	7.7	53.9	-25.3	-28.7	5.0	-2.0	-3.0
**17**	171.1	75.5	67.9	94.1	10.8	60.2	-28.4	-31.9	6.3	-1.1	-5.2
**18**	161.2	56.7	53.1	81.1	9.3	64.2	-31.8	-32.4	6.9	-2.0	-4.8
**19**	156.8	62.6	60.6	86.4	7.0	60.3	-29.2	-31.1	3.4	-1.6	-1.7
**20**	180.1	76.6	76.6	101.4	9.7	65.3	-32.7	-32.7	3.9	-1.9	-1.9
**21**	180.5	81.3	80.2	104.5	9.5	61.5	-30.1	-31.4	5.2	-2.7	-2.6
**22**	182.3	83.4	80.5	104.1	11.4	62.6	-30.5	-32.1	4.5	-1.6	-2.9

Table S19. Individual components and detailed contributions (MHz) of the hyperfine coupling constants for the Mo-based complexes obtained using the M05 functional.

	A_{11}	A_{22}	A_{33}	A^{FC}	A^{PC}	A_{11}^{SD}	A_{22}^{SD}	A_{33}^{SD}	$A_{11}^{SOC,an}$	$A_{22}^{SOC,an}$	$A_{33}^{SOC,an}$
**1**	144.2	133.3	61.9	105.7	7.4	27.6	17.3	-44.9	3.5	2.9	-6.5
**2**	139.5	137.7	59.7	104.9	7.4	24.0	22.2	-46.2	3.3	3.3	-6.6
**3**	151.0	68.7	68.7	88.9	7.2	52.2	-26.1	-26.1	2.9	-1.4	-1.5
**5**	148.6	67.8	64.0	84.6	8.8	51.6	-24.5	-27.1	3.7	-1.3	-2.4
**6**	149.4	63.5	61.3	83.6	7.8	53.8	-26.1	-27.7	4.4	-1.9	-2.5
**7**	144.8	61.1	56.1	69.1	18.2	43.2	-22.9	-20.3	14.4	-3.4	-11.0
**8**	154.7	70.0	67.2	88.3	9.0	54.1	-25.8	-28.3	3.4	-1.5	-1.9
**9**	149.4	69.7	64.7	85.7	8.9	52.0	-23.9	-28.1	3.0	-1.1	-1.9
**10**	176.3	60.2	47.9	71.6	23.2	52.7	-26.2	-26.5	29.0	-8.5	-20.4
**11**	158.4	66.1	60.2	82.2	12.7	57.2	-26.1	-31.0	6.5	-2.7	-3.8
**14**	159.7	63.4	59.0	84.1	10.0	60.6	-29.0	-31.6	5.3	-1.7	-3.6
**15**	150.9	59.2	55.5	79.0	9.5	56.2	-26.5	-29.7	6.4	-2.9	-3.5
**16**	164.6	77.7	74.6	94.3	11.3	52.5	-24.9	-27.6	6.6	-3.1	-3.5
**17**	156.4	62.7	53.2	74.6	16.2	57.5	-27.2	-30.3	8.4	-1.0	-7.3
**18**	135.0	30.1	26.2	50.3	13.5	62.2	-31.2	-30.9	9.3	-2.6	-6.7
**19**	166.6	71.6	69.9	93.0	9.8	58.9	-28.5	-30.5	5.2	-2.7	-2.5
**20**	177.2	73.4	73.4	93.7	14.3	63.2	-31.6	-31.6	6.2	-3.1	-3.1
**21**	168.8	67.5	66.7	87.0	14.1	59.8	-29.2	-30.6	8.1	-4.4	-3.7
**22**	165.4	66.8	63.6	81.7	16.9	60.4	-29.8	-30.6	6.6	-2.1	-4.5

Table S20. Individual components and detailed contributions (MHz) of the hyperfine coupling constants for the Mo-based complexes obtained using the M06-2X functional.

	A_{11}	A_{22}	A_{33}	A^{FC}	A^{PC}	A_{11}^{SD}	A_{22}^{SD}	A_{33}^{SD}	$A_{11}^{SOC,an}$	$A_{22}^{SOC,an}$	$A_{33}^{SOC,an}$
**1**	-65.6	28.9	16.9	-19.1	12.5	-52.5	32.1	20.5	-6.6	3.5	3.1
**2**	-66.6	27.6	25.5	-16.4	11.9	-55.5	28.8	26.7	-6.8	3.4	3.4
**3**	72.0	-23.7	-23.7	-1.4	9.6	60.8	-30.4	-30.4	3.2	-1.6	-1.6
**5**	91.6	-7.9	-2.3	16.1	11.1	60.4	-32.3	-28.0	4.3	-2.8	-1.5
**6**	88.4	-13.1	-9.3	11.8	10.2	61.6	-32.2	-29.4	5.0	-3.0	-2.0
**7**	106.1	12.4	11.5	25.0	18.4	47.9	-25.0	-22.9	15.0	-6.0	-9.0

**8**	91.8	-9.5	-4.9	14.4	11.4	61.9	-32.8	-29.1	4.3	-2.6	-1.7
**9**	86.3	-11.9	-6.2	11.5	11.2	60.1	-32.4	-27.7	3.6	-2.4	-1.3
**10**	183.3	42.6	27.5	55.0	29.4	59.3	-29.6	-29.7	39.7	-12.4	-27.3
**11**	124.3	20.0	12.8	37.9	14.5	64.5	-29.4	-35.2	7.6	-3.1	-4.5
**14**	118.2	13.5	8.8	35.1	11.8	65.2	-31.2	-34.0	6.4	-2.2	-4.1
**15**	109.2	5.2	4.3	28.1	11.5	62.9	-31.1	-31.8	6.9	-3.4	-3.5
**16**	96.8	5.0	2.3	19.7	15.1	56.3	-27.0	-29.3	5.9	-2.8	-3.2
**17**	132.5	37.4	23.2	46.0	18.5	60.8	-26.5	-34.3	7.6	-0.6	-7.0
**18**	136.8	23.3	18.9	45.4	14.3	67.4	-33.4	-34.0	10.0	-3.1	-6.8
**19**	105.6	-0.4	-1.3	22.7	11.9	65.3	-32.1	-33.2	5.9	-3.0	-2.9
**20**	142.3	26.6	26.6	49.6	15.6	70.1	-35.0	-35.0	7.3	-3.6	-3.6
**21**	156.1	43.0	42.6	65.0	15.5	66.0	-32.5	-33.5	9.8	-5.1	-4.6
**22**	158.1	45.5	42.8	64.1	18.0	67.8	-33.3	-34.5	8.4	-3.5	-4.9

Table S21. Individual components and detailed contributions (MHz) of the hyperfine coupling constants for the Mo-based complexes obtained using the MN15 functional.

	A_{11}	A_{22}	A_{33}	A^{FC}	A^{PC}	A_{11}^{SD}	A_{22}^{SD}	A_{33}^{SD}	$A_{11}^{SOC,an}$	$A_{22}^{SOC,an}$	$A_{33}^{SOC,an}$
**1**	60.0	50.4	-5.2	28.4	6.5	22.9	13.8	-36.7	2.1	1.6	-3.7
**2**	58.4	56.7	-6.3	29.7	6.4	20.3	18.7	-39.0	1.9	1.8	-3.7
**3**	78.0	1.5	1.5	21.2	5.6	49.1	-24.5	-24.5	2.1	-1.0	-1.1
**5**	85.1	11.2	6.6	27.7	6.5	48.4	-22.1	-26.3	2.6	-1.1	-1.5
**6**	80.3	2.6	-1.0	20.8	6.3	50.1	-23.4	-26.7	3.1	-1.4	-1.7
**7**	88.2	15.8	14.7	30.2	9.3	43.0	-19.9	-23.2	5.7	-3.9	-1.8
**8**	86.1	8.1	4.5	25.9	6.9	51.0	-23.9	-27.2	2.3	-1.0	-1.3
**9**	81.6	7.6	3.4	24.1	6.7	49.0	-22.8	-26.2	1.9	-0.6	-1.3
**10**	112.7	19.5	19.3	38.4	12.0	49.4	-27.1	-22.4	12.9	-4.0	-8.9
**11**	106.9	23.1	17.0	40.1	8.7	54.0	-24.3	-29.7	4.1	-1.6	-2.4
**14**	99.5	13.5	9.7	32.9	7.8	54.8	-26.1	-28.6	4.0	-1.3	-2.6
**15**	89.1	8.0	3.8	26.1	7.4	51.1	-23.8	-27.3	4.5	-1.9	-2.5
**16**	86.4	14.2	10.8	28.3	8.6	44.7	-20.9	-23.8	4.7	-2.1	-2.6
**17**	107.7	29.6	25.1	43.1	10.8	47.8	-23.2	-24.6	5.9	-1.5	-4.4
**18**	104.9	12.9	12.4	33.7	9.5	55.2	-28.5	-26.7	6.4	-2.1	-4.4
**19**	108.9	21.5	19.5	42.1	7.7	55.5	-26.8	-28.8	3.6	-1.8	-1.8
**20**	142.2	44.4	44.4	67.1	9.7	61.1	-30.6	-30.6	4.3	-2.1	-2.1
**21**	137.3	45.9	45.7	66.4	9.7	56.0	-27.9	-28.1	5.2	-2.6	-2.6
**22**	143.4	51.6	49.2	70.3	10.9	57.4	-28.0	-29.4	4.8	-1.9	-2.9

Table S22. Individual components and detailed contributions (MHz) of the hyperfine coupling constants for the Mo-based complexes obtained using the M06 functional.

	A_{11}	A_{22}	A_{33}	A^{FC}	A^{PC}	A_{11}^{SD}	A_{22}^{SD}	A_{33}^{SD}	$A_{11}^{SOC,an}$	$A_{22}^{SOC,an}$	$A_{33}^{SOC,an}$
**1**	68.9	57.7	-14.6	29.1	8.3	28.3	17.6	-45.9	3.3	2.8	-6.1
**2**	65.1	63.2	-16.0	29.4	8.1	24.7	22.7	-47.4	3.1	3.1	-6.2
**3**	76.0	-6.9	-6.8	13.2	7.6	52.9	-26.4	-26.5	2.5	-1.3	-1.3
**5**	78.4	-7.2	-2.7	13.7	9.2	52.4	-28.0	-24.5	3.3	-2.2	-1.1
**6**	78.5	-9.8	-7.9	12.0	8.3	54.5	-28.0	-26.4	4.0	-2.1	-1.9
**7**	81.6	-2.8	-0.4	9.2	17.0	43.4	-22.4	-21.1	12.2	-6.6	-5.6
**8**	81.5	-6.6	-3.4	14.4	9.5	54.9	-28.8	-26.0	3.0	-1.7	-1.3
**9**	78.9	-6.6	-1.7	14.2	9.4	53.0	-28.5	-24.5	2.5	-1.7	-0.9
**10**	109.1	-14.6	-2.5	9.0	21.7	53.4	-27.3	-26.0	25.2	-18.0	-7.2
**11**	84.4	-14.2	-8.1	7.8	12.9	58.0	-31.6	-26.5	5.8	-3.4	-2.4
**14**	72.9	-28.4	-22.9	-3.2	10.5	60.9	-32.3	-28.6	5.0	-3.4	-1.6
**15**	76.5	-19.0	-14.6	4.4	10.0	56.6	-30.1	-26.5	5.9	-3.4	-2.5
**16**	82.8	-7.5	-4.1	12.2	11.6	53.1	-27.9	-25.2	6.1	-3.4	-2.7
**17**	68.1	-34.2	-24.7	-12.6	15.7	57.6	-30.9	-26.8	7.6	-6.4	-1.1
**18**	-54.6	53.6	-50.0	-30.4	13.6	-31.6	62.4	-30.7	-6.1	8.5	-2.4
**19**	89.0	-8.3	-7.8	14.0	10.4	60.2	-30.4	-29.9	4.7	-2.3	-2.4
**20**	97.9	-6.9	-6.9	13.7	14.3	64.5	-32.3	-32.3	5.6	-2.8	-2.8
**21**	94.0	-7.1	-6.6	12.7	14.1	60.3	-30.6	-29.7	7.2	-3.4	-3.8
**22**	93.3	-9.1	-5.7	9.5	16.7	61.3	-31.3	-30.0	6.0	-4.0	-2.0

Table S23. Individual components and detailed contributions (MHz) of the hyperfine coupling constants for the Mo-based complexes obtained using the ωB97 functional.

	A_{11}	A_{22}	A_{33}	A^{FC}	A^{PC}	A_{11}^{SD}	A_{22}^{SD}	A_{33}^{SD}	$A_{11}^{SOC,an}$	$A_{22}^{SOC,an}$	$A_{33}^{SOC,an}$
**1**	140.9	128.8	46.4	99.2	6.2	33.1	21.0	-54.1	2.5	2.4	-5.0
**2**	133.8	131.7	41.1	96.4	5.8	29.3	27.1	-56.3	2.4	2.5	-4.9
**3**	174.1	80.1	80.1	106.6	4.8	61.3	-30.7	-30.6	1.6	-0.8	-0.8
**5**	181.3	90.3	86.2	113.2	6.0	60.1	-28.5	-31.6	2.1	-0.5	-1.5
**6**	180.7	85.7	82.6	110.9	5.4	61.7	-29.7	-32.0	2.8	-1.0	-1.8
**7**	156.6	84.4	79.5	97.0	9.7	43.0	-21.2	-21.8	6.9	-1.3	-5.6
**8**	184.0	91.1	87.1	114.5	6.2	61.3	-29.0	-32.3	2.1	-0.7	-1.4
**9**	178.2	89.4	84.1	111.2	6.0	59.6	-27.5	-32.0	1.6	-0.4	-1.2
**10**	218.8	112.4	98.8	128.1	15.2	56.4	-26.1	-30.3	19.2	-4.9	-14.4
**11**	201.2	103.5	97.4	125.7	8.3	63.0	-28.8	-34.2	4.4	-1.8	-2.6
**14**	200.7	99.8	95.7	125.4	6.7	64.9	-31.2	-33.8	3.9	-1.2	-2.7
**15**	191.2	91.4	90.8	118.0	6.4	62.8	-31.2	-31.6	4.1	-2.0	-2.1
**16**	200.7	114.3	108.2	133.0	8.0	56.2	-24.9	-31.3	3.6	-1.9	-1.7
**17**	215.4	127.1	111.1	140.6	10.6	59.4	-23.5	-35.9	4.9	-0.7	-4.2
**18**	196.8	90.3	86.2	115.7	8.7	66.1	-32.4	-33.7	6.5	-1.9	-4.6
**19**	196.4	95.7	94.2	122.2	6.5	64.5	-31.5	-33.0	3.4	-1.7	-1.7

**20**	217.0	108.7	108.7	135.9	8.9	68.3	-34.2	-34.2	4.1	-2.1	-2.1
**21**	217.8	113.6	112.2	138.7	9.1	64.6	-31.4	-33.2	5.5	-2.9	-2.6
**22**	217.2	113.0	110.6	136.2	10.7	65.7	-32.1	-33.6	4.8	-1.9	-2.8

Table 24. Individual components and detailed contributions (MHz) of the hyperfine coupling constants for the Mo-based complexes obtained using the CAM-B3LYP functional.

	A_{11}	A_{22}	A_{33}	A^{FC}	A^{PC}	A_{11}^{SD}	A_{22}^{SD}	A_{33}^{SD}	$A_{11}^{SOC,an}$	$A_{22}^{SOC,an}$	$A_{33}^{SOC,an}$
**1**	112.5	100.7	23.0	72.5	6.3	31.2	19.7	-50.9	2.7	2.3	-5.0
**2**	106.6	104.6	19.3	70.8	6.1	27.4	25.4	-52.8	2.4	2.5	-4.9
**3**	139.2	49.6	49.6	74.1	5.4	58.2	-29.1	-29.1	1.8	-0.9	-0.9
**5**	148.1	61.4	57.2	82.5	6.5	57.1	-26.9	-30.2	2.3	-0.7	-1.6
**6**	149.3	57.4	55.1	81.4	5.9	59.2	-28.7	-30.5	3.0	-1.2	-1.8
**7**	136.2	61.6	57.1	74.3	10.7	44.1	-21.9	-22.3	7.3	-1.6	-5.7
**8**	150.8	61.4	57.6	83.2	6.7	58.9	-27.8	-31.1	2.1	-0.8	-1.3
**9**	145.3	60.2	54.8	80.2	6.6	57.1	-26.3	-30.8	1.6	-0.4	-1.3
**10**	192.2	86.0	72.9	101.5	15.6	56.3	-26.2	-30.1	19.0	-4.9	-14.1
**11**	169.7	73.7	67.7	94.7	9.1	61.8	-28.3	-33.5	4.4	-1.8	-2.7
**14**	169.1	69.1	64.8	93.6	7.5	64.1	-30.7	-33.4	4.2	-1.3	-2.8
**15**	160.9	64.1	61.7	88.6	7.0	61.0	-29.6	-31.4	4.5	-2.0	-2.6
**16**	162.3	70.6	70.4	92.7	8.5	56.8	-28.0	-28.7	4.6	-2.6	-2.0
**17**	178.2	83.2	72.5	99.9	11.4	61.2	-27.3	-33.9	5.9	-1.0	-4.9
**18**	168.3	61.2	57.0	85.9	9.6	66.1	-32.4	-33.6	6.9	-2.0	-4.9
**19**	164.4	65.4	63.7	90.5	7.3	63.3	-30.8	-32.5	3.5	-1.7	-1.8
**20**	186.1	78.5	78.5	104.5	9.9	67.8	-33.9	-33.9	4.1	-2.1	-2.1
**21**	187.8	84.7	83.3	108.7	9.9	63.9	-31.1	-32.7	5.5	-2.9	-2.7
**22**	189.6	86.1	83.5	108.1	11.7	65.2	-31.8	-33.4	4.9	-1.9	-2.9

Table S25. Individual components and detailed contributions (MHz) of the hyperfine coupling constants for the Mo-based complexes obtained using the LC-PBE functional.

	A_{11}	A_{22}	A_{33}	A^{FC}	A^{PC}	A_{11}^{SD}	A_{22}^{SD}	A_{33}^{SD}	$A_{11}^{SOC,an}$	$A_{22}^{SOC,an}$	$A_{33}^{SOC,an}$
**1**	103.3	91.6	10.2	61.1	7.3	32.5	20.8	-53.3	2.6	2.5	-5.0
**2**	96.9	94.9	5.1	58.7	6.9	28.9	26.8	-55.7	2.5	2.5	-5.0
**3**	133.5	39.1	39.1	64.7	5.8	61.5	-30.7	-30.7	1.7	-0.8	-0.9
**5**	143.7	53.5	48.4	74.9	6.9	59.7	-27.9	-31.9	2.3	-0.6	-1.7
**6**	141.6	47.8	44.0	71.4	6.4	61.0	-29.0	-31.9	3.0	-1.1	-1.9
**7**	128.6	58.6	53.1	69.7	10.3	41.6	-20.3	-21.3	7.1	-1.3	-5.8
**8**	144.9	52.7	48.1	74.7	7.2	60.9	-28.4	-32.4	2.3	-0.8	-1.5
**9**	140.6	52.5	46.6	72.9	6.9	59.2	-27.1	-32.1	1.7	-0.4	-1.3
**10**	191.5	85.9	70.6	99.5	16.4	55.5	-25.3	-30.3	20.1	-4.9	-15.1
**11**	163.0	66.2	59.6	87.0	9.2	62.4	-28.3	-34.1	4.5	-1.8	-2.7
**14**	157.4	58.3	54.0	82.2	7.7	63.6	-30.5	-33.1	4.1	-1.3	-2.9

**15**	151.0	52.3	52.2	77.8	7.3	61.8	-30.9	-30.9	4.2	-2.1	-2.1
**16**	154.6	74.6	64.4	88.4	9.4	53.6	-21.7	-31.8	3.3	-1.6	-1.8
**17**	170.3	89.9	68.6	97.9	11.7	56.4	-19.4	-37.0	4.4	-0.4	-4.0
**18**	158.1	54.1	49.4	77.7	9.5	64.6	-31.4	-33.2	6.5	-1.8	-4.7
**19**	154.4	54.5	53.1	79.8	7.5	63.9	-31.2	-32.7	3.4	-1.7	-1.7
**20**	175.4	67.7	67.7	93.8	9.8	67.9	-34.0	-34.0	4.1	-2.0	-2.0
**21**	179.5	77.1	75.6	100.7	10.0	63.4	-30.8	-32.6	5.5	-2.9	-2.6
**22**	181.3	78.0	75.6	100.0	11.6	65.1	-31.8	-33.3	4.8	-2.0	-2.8

Table S26. Individual components and detailed contributions (MHz) of the hyperfine coupling constants for the Mo-based complexes obtained using the LC-BLYP functional.

	A_{11}	A_{22}	A_{33}	A^{FC}	A^{PC}	A_{11}^{SD}	A_{22}^{SD}	A_{33}^{SD}	$A_{11}^{SOC,an}$	$A_{22}^{SOC,an}$	$A_{33}^{SOC,an}$
**1**	101.8	89.9	8.8	61.0	5.9	32.5	20.8	-53.3	2.6	2.3	-4.9
**2**	95.7	93.7	5.5	59.3	5.7	28.5	26.4	-54.9	2.4	2.4	-4.8
**3**	129.2	38.7	38.8	63.7	5.2	59.0	-29.5	-29.5	1.5	-0.7	-0.7
**5**	137.5	50.5	46.1	71.8	6.3	57.8	-27.2	-30.7	1.8	-0.4	-1.4
**6**	138.4	46.3	43.5	70.4	5.7	60.0	-28.9	-31.1	2.6	-1.0	-1.6
**7**	123.8	52.5	47.1	64.1	10.4	42.8	-20.9	-22.0	6.6	-1.2	-5.5
**8**	140.1	50.4	46.3	72.4	6.6	59.6	-28.1	-31.6	1.8	-0.6	-1.2
**9**	135.2	49.8	44.1	70.0	6.4	57.7	-26.5	-31.3	1.3	-0.2	-1.1
**10**	179.3	75.2	61.8	90.0	15.4	55.6	-25.7	-29.9	18.4	-4.5	-13.9
**11**	156.6	60.7	54.8	81.9	8.9	62.1	-28.5	-33.6	4.1	-1.6	-2.4
**14**	155.4	55.2	50.2	79.7	7.3	64.9	-30.7	-34.2	3.8	-1.1	-2.6
**15**	148.2	50.6	49.2	75.9	6.8	61.7	-30.4	-31.3	4.1	-1.8	-2.3
**16**	148.3	56.0	54.7	78.3	8.1	58.0	-28.1	-30.0	4.1	-2.4	-1.7
**17**	159.8	64.8	52.8	81.5	11.0	62.1	-27.0	-35.1	5.5	-0.8	-4.7
**18**	149.8	43.8	38.2	68.0	9.4	66.3	-31.8	-34.5	6.5	-1.8	-4.7
**19**	151.0	52.0	50.5	77.5	7.1	63.6	-31.1	-32.5	3.1	-1.5	-1.6
**20**	169.8	62.9	62.9	88.9	9.6	67.9	-33.9	-33.9	3.6	-1.8	-1.8
**21**	170.8	68.3	67.1	92.4	9.7	64.0	-31.2	-32.8	4.9	-2.6	-2.3
**22**	172.4	70.0	66.9	91.6	11.5	65.3	-31.7	-33.5	4.2	-1.5	-2.8

Table S27. Individual components and detailed contributions (MHz) of the hyperfine coupling constants for the Mo-based complexes obtained using the ωB2PLYP functional.

	A_{11}	A_{22}	A_{33}	A^{FC}	A^{PC}	A_{11}^{SD}	A_{22}^{SD}	A_{33}^{SD}	$A_{11}^{SOC,an}$	$A_{22}^{SOC,an}$	$A_{33}^{SOC,an}$
**1**	122.9	110.7	31.7	83.8	4.8	31.0	19.5	-50.5	3.5	2.8	-6.3
**2**	118.2	116.1	30.8	83.5	4.9	27.0	24.9	-51.9	2.9	2.9	-5.8
**3**	155.5	64.6	64.7	89.7	5.3	58.4	-29.2	-29.2	2.3	-1.1	-1.2
**5**	163.9	74.5	70.9	96.8	6.3	58.2	-27.7	-30.5	2.8	-0.9	-1.8
**6**	164.7	70.7	68.2	95.5	5.7	60.3	-29.2	-31.0	3.4	-1.4	-2.0
**7**	161.6	77.1	74.9	92.9	11.6	49.4	-25.2	-24.2	7.8	-2.4	-5.4

**8**	166.1	74.2	71.2	97.3	6.6	59.9	-28.6	-31.2	2.6	-1.1	-1.5
**9**	160.0	72.7	67.8	93.8	6.4	57.9	-26.9	-31.0	2.1	-0.6	-1.5
**10**	212.4	98.9	89.7	116.6	17.1	56.8	-28.1	-28.8	22.0	-6.8	-15.3
**11**	187.2	87.4	82.7	110.0	9.2	63.0	-29.6	-33.4	5.2	-2.1	-3.1
**14**	183.3	79.7	76.8	106.2	7.1	65.7	-32.1	-33.6	4.5	-1.5	-3.0
**15**	175.9	76.9	73.7	102.1	6.8	62.3	-29.8	-32.5	4.9	-2.2	-2.7
**16**	152.9	67.5	24.6	76.1	5.7	68.2	-12.2	-56.0	3.2	-2.0	-1.2
**17**	168.9	64.5	40.0	80.9	10.3	72.9	-20.1	-52.8	5.1	-6.5	1.5
**18**	184.2	72.8	72.2	100.3	9.5	67.1	-34.7	-32.5	7.6	-2.4	-5.1
**19**	181.4	81.3	78.9	106.7	7.3	63.5	-30.5	-33.0	4.2	-2.1	-2.1
**20**	204.9	94.7	94.7	121.5	10.0	68.4	-34.2	-34.2	5.2	-2.6	-2.6
**21**	203.5	96.5	94.1	121.5	9.9	65.9	-31.6	-34.3	6.5	-3.4	-3.1
**22**	206.8	99.7	95.8	122.3	11.8	67.1	-32.6	-34.5	5.7	-1.9	-3.9

Table S28. Individual components and detailed contributions (MHz) of the hyperfine coupling constants for the Mo-based complexes obtained using the $Pr^2SCAN50$ functional.

	A_{11}	A_{22}	A_{33}	A^{FC}	A^{PC}	A_{11}^{SD}	A_{22}^{SD}	A_{33}^{SD}	$A_{11}^{SOC,an}$	$A_{22}^{SOC,an}$	$A_{33}^{SOC,an}$
**1**	114.1	101.9	28.4	79.5	2.0	29.1	17.8	-46.9	3.6	2.7	-6.3
**2**	114.5	112.5	36.3	85.3	2.5	24.0	22.1	-46.1	2.8	2.7	-5.5
**3**	144.1	64.9	64.9	87.2	4.1	51.0	-25.5	-25.5	1.9	-1.0	-1.0
**5**	149.8	69.9	67.2	90.7	4.9	52.1	-25.1	-27.0	2.2	-0.8	-1.5
**6**	152.8	69.1	66.0	91.6	4.4	54.1	-25.7	-28.4	2.9	-1.2	-1.7
**7**	141.9	70.3	65.9	83.3	9.4	43.1	-21.1	-22.0	6.2	-1.4	-4.9
**8**	153.3	70.7	67.8	92.1	5.2	54.3	-25.9	-28.4	1.9	-0.8	-1.2
**9**	146.5	68.7	63.4	87.8	5.1	52.2	-23.9	-28.3	1.6	-0.4	-1.2
**10**	199.9	98.6	95.0	118.3	12.8	52.7	-27.4	-25.3	16.2	-5.2	-11.0
**11**	177.1	86.1	81.5	107.4	7.5	58.2	-27.3	-30.9	4.2	-1.6	-2.6
**14**	173.1	76.0	75.1	102.4	5.7	61.4	-30.8	-30.5	3.8	-1.3	-2.5
**15**	165.4	76.2	71.0	98.8	5.4	57.0	-26.1	-30.8	4.4	-1.9	-2.5
**16**	145.7	54.3	27.8	70.9	5.1	64.4	-18.3	-46.1	5.6	-3.4	-2.2
**17**	166.9	63.3	42.9	82.0	9.1	69.8	-20.7	-49.2	6.2	-7.1	0.9
**18**	179.2	76.5	72.6	101.4	8.1	63.2	-28.4	-34.8	6.7	-4.6	-2.1
**19**	174.8	85.0	81.8	107.9	5.9	57.6	-27.2	-30.4	3.5	-1.8	-1.8
**20**	198.4	97.3	97.3	122.6	8.4	63.3	-31.7	-31.7	4.2	-2.1	-2.1
**21**	190.8	90.6	87.7	114.8	8.2	62.7	-29.8	-32.9	5.2	-2.7	-2.5
**22**	197.2	97.4	93.2	119.3	9.9	63.4	-30.6	-32.8	4.6	-1.3	-3.3

Table S29. Individual components and detailed contributions (MHz) of the hyperfine coupling constants for the Mo-based complexes obtained using the ωB88PP86 functional.

	A_{11}	A_{22}	A_{33}	A^{FC}	A^{PC}	A_{11}^{SD}	A_{22}^{SD}	A_{33}^{SD}	$A_{11}^{SOC,an}$	$A_{22}^{SOC,an}$	$A_{33}^{SOC,an}$
**1**	116.3	104.3	28.9	81.5	1.8	28.7	17.9	-46.6	4.5	3.2	-7.7
**2**	115.7	112.8	34.9	85.2	2.7	24.7	22.0	-46.7	3.3	3.1	-6.3
**3**	147.8	64.3	64.2	87.6	4.6	53.6	-26.8	-26.8	2.3	-1.1	-1.1
**5**	154.9	70.4	67.4	92.2	5.5	55.0	-26.4	-28.6	2.5	-0.8	-1.7
**6**	155.1	66.7	63.0	90.2	4.8	57.2	-27.2	-30.0	3.2	-1.2	-2.0
**7**	162.3	76.2	75.0	92.8	11.7	50.4	-25.9	-24.6	7.6	-2.5	-5.0
**8**	156.7	69.9	66.8	92.1	5.8	57.0	-27.2	-29.8	2.1	-0.8	-1.3
**9**	150.4	68.5	63.1	88.5	5.6	54.9	-25.2	-29.7	1.7	-0.4	-1.3
**10**	204.0	91.2	87.5	110.6	17.0	54.6	-28.9	-25.7	22.0	-7.5	-14.5
**11**	179.8	84.9	78.1	105.8	8.5	60.9	-28.0	-32.9	4.9	-1.5	-3.3
**14**	170.6	68.7	66.5	96.2	5.8	65.3	-32.2	-33.1	3.5	-1.1	-2.4
**15**	165.8	71.6	65.5	95.2	5.9	60.3	-27.5	-32.8	4.7	-2.0	-2.8
**16**	137.3	59.4	7.0	63.2	4.9	68.8	-5.7	-63.1	0.9	-2.8	2.0
**17**	156.3	55.7	28.9	70.6	9.9	73.0	-17.3	-55.7	3.3	-7.3	4.0
**18**	174.4	66.8	63.3	92.8	8.8	65.7	-29.7	-36.0	7.5	-5.1	-2.4
**19**	174.4	80.7	77.6	104.3	6.6	59.6	-28.3	-31.4	4.1	-2.0	-2.1
**20**	198.8	93.6	93.6	119.2	9.5	65.3	-32.6	-32.6	5.0	-2.5	-2.5
**21**	189.6	85.3	81.6	109.9	9.0	65.4	-30.7	-34.7	5.6	-3.0	-2.7
**22**	196.9	95.1	85.8	114.8	11.2	66.3	-30.3	-35.9	4.9	-0.6	-4.3

Table S30. Individual components and detailed contributions (MHz) of the hyperfine coupling constants for the Mo-based complexes obtained using the B2GP-PLYP functional.

	A_{11}	A_{22}	A_{33}	A^{FC}	A^{PC}	A_{11}^{SD}	A_{22}^{SD}	A_{33}^{SD}	$A_{11}^{SOC,an}$	$A_{22}^{SOC,an}$	$A_{33}^{SOC,an}$
**1**	113.9	101.6	26.1	79.3	1.4	28.8	17.8	-46.6	4.6	3.3	-7.9
**2**	113.6	111.6	33.9	84.0	2.4	24.0	22.1	-46.1	3.3	3.2	-6.5
**3**	147.3	64.7	64.7	87.8	4.4	52.9	-26.5	-26.4	2.3	-1.2	-1.2
**5**	153.9	70.8	67.9	92.2	5.4	53.9	-25.9	-28.1	2.6	-0.9	-1.7
**6**	156.5	68.5	65.0	92.0	4.7	56.7	-27.0	-29.7	3.3	-1.3	-2.0
**7**	161.0	75.4	73.9	91.6	11.8	49.7	-25.5	-24.2	8.0	-2.6	-5.4
**8**	156.7	70.8	68.0	92.9	5.7	56.2	-26.9	-29.3	2.2	-0.8	-1.3
**9**	150.0	69.0	63.9	88.8	5.6	54.1	-24.9	-29.2	1.8	-0.4	-1.4
**10**	203.5	91.2	87.9	111.1	16.5	54.9	-29.1	-25.7	21.3	-7.3	-14.0
**11**	180.9	85.8	80.0	107.2	8.5	60.6	-28.2	-32.4	4.9	-1.7	-3.2
**14**	175.4	73.4	71.4	100.8	6.0	64.9	-32.1	-32.8	4.0	-1.3	-2.6
**15**	168.7	74.9	68.6	98.3	5.9	59.8	-27.2	-32.5	5.0	-2.0	-3.0
**16**	125.1	43.7	-30.1	43.0	3.4	75.9	0.5	-76.4	3.2	-3.0	-0.2
**17**	142.0	39.7	-13.7	47.0	9.1	82.2	-7.8	-74.4	4.0	-8.5	4.5
**18**	179.6	70.5	67.9	97.1	8.9	66.1	-30.4	-35.7	7.7	-5.2	-2.5
**19**	176.8	82.2	79.2	106.2	6.6	60.0	-28.5	-31.6	4.2	-2.1	-2.1

**20**	201.7	95.7	95.7	121.5	9.6	65.7	-32.9	-32.9	5.1	-2.6	-2.6
**21**	194.5	89.7	86.5	114.6	9.1	65.2	-30.9	-34.3	6.0	-3.1	-2.9
**22**	200.6	97.7	90.2	118.4	11.2	66.1	-30.9	-35.1	5.2	-1.0	-4.2

Table S31. Individual components and detailed contributions (MHz) of the hyperfine coupling constants for the Mo-based complexes obtained using the B2PLYP functional.

	A_{11}	A_{22}	A_{33}	A^{FC}	A^{PC}	A_{11}^{SD}	A_{22}^{SD}	A_{33}^{SD}	$A_{11}^{SOC,an}$	$A_{22}^{SOC,an}$	$A_{33}^{SOC,an}$
**1**	105.0	93.1	21.2	71.3	1.9	28.3	17.4	-45.6	3.7	2.7	-6.4
**2**	104.0	102.1	27.0	75.2	2.6	23.5	21.7	-45.2	2.9	2.8	-5.6
**3**	137.3	57.6	57.6	79.9	4.3	51.1	-25.6	-25.5	2.1	-1.1	-1.1
**5**	143.9	64.6	61.5	84.9	5.2	51.6	-24.6	-27.0	2.4	-0.9	-1.5
**6**	148.0	63.1	60.0	85.8	4.6	54.7	-26.1	-28.6	3.1	-1.2	-1.9
**7**	144.5	66.2	63.6	80.3	11.1	45.5	-23.1	-22.4	7.7	-2.2	-5.5
**8**	148.7	65.5	62.8	86.8	5.6	54.4	-26.1	-28.4	2.1	-0.8	-1.2
**9**	141.9	63.7	58.6	82.7	5.5	52.3	-24.1	-28.2	1.7	-0.4	-1.3
**10**	191.6	84.5	80.8	103.8	15.2	53.6	-28.2	-25.4	19.3	-6.4	-12.9
**11**	172.6	79.7	74.8	100.8	8.3	59.1	-27.7	-31.4	4.6	-1.7	-2.9
**14**	171.3	72.3	69.9	98.3	6.2	62.9	-30.9	-32.0	4.1	-1.4	-2.7
**15**	161.4	70.9	65.0	93.4	5.8	57.7	-26.4	-31.3	4.8	-1.9	-2.9
**16**	134.5	40.5	6.7	56.7	3.9	66.7	-17.3	-49.4	7.4	-2.8	-4.6
**17**	152.1	43.9	16.0	62.2	8.5	73.4	-19.8	-53.6	8.3	-7.0	-1.3
**18**	172.3	64.4	63.7	91.3	8.9	65.0	-30.8	-34.2	7.4	-5.0	-2.3
**19**	169.3	75.8	72.9	99.6	6.5	59.6	-28.4	-31.2	3.9	-1.9	-2.0
**20**	194.5	89.8	89.8	115.4	9.5	65.3	-32.6	-32.6	4.8	-2.4	-2.4
**21**	190.5	88.3	86.1	112.7	9.0	63.2	-30.4	-32.8	5.9	-3.0	-2.9
**22**	194.5	93.2	88.5	114.4	11.1	64.2	-31.0	-33.2	5.1	-1.3	-3.8

Table S32. Calculated HFCs (in MHz) combining the A^{FC} and A^{SD} terms from DLPNO-CCSD and the A^{SO} term from PBE0-DH for 17 Mo(V) complexes of the benchmark set.

	A_{11}	A_{22}	A_{33}	A_{iso}
**1**	135.4	122.3	42.2	100.0
**2**	148.1	78.5	50.0	92.2
**3**	175.5	81.5	81.2	112.7
**5**	180.6	88.2	84.0	117.6
**6**	184.4	88.3	85.8	119.5
**7**	159.9	80.7	76.8	105.8
**8**	185.2	90.6	87.0	120.9
**9**	178.9	88.0	82.7	116.5
**10**	175.4	121.7	43.4	113.5
**11**	205.7	103.0	97.2	135.3
**14**	204.0	98.9	97.2	133.3
**15**	198.3	97.7	94.1	130.1
**17**	209.5	101.1	104.1	138.3
**18**	207.6	96.1	90.5	131.4
**19**	205.7	102.4	99.1	135.7
**20**	233.0	120.6	120.2	158.0
**22**	233.7	122.4	120.2	158.8

Complete DFT results for *g*-tensors

Table S33. Individual components of the *g*-tensors and *g*-shifts (in parts per thousand) for the Mo-based complexes obtained using the BLYP functional.

	Mo spin pop.	g_{11}	g_{22}	g_{33}	Δg_{11}	Δg_{22}	Δg_{33}
**1**	0.81	2.031	2.030	1.999	28	27	-3
**2**	0.79	2.019	2.019	2.000	17	16	-3
**3**	0.72	2.028	1.993	1.992	25	-10	-10
**4**	0.75	2.025	1.994	1.986	23	-8	-16
**5**	0.75	2.028	1.986	1.966	26	-16	-36
**6**	0.76	2.027	1.988	1.962	25	-14	-41
**7**	0.61	2.007	1.958	1.902	29	-45	-100
**8**	0.81	2.032	1.976	1.974	28	-26	-28
**9**	0.77	2.035	1.978	1.968	33	-24	-35
**10**	0.74	2.006	1.961	1.877	4	-41	-125
**11**	0.83	2.005	1.970	1.958	2	-32	-45
**12**	0.83	2.004	1.952	1.832	2	-50	-170
**13**	0.90	1.971	1.964	1.946	-31	-38	-56
**14**	0.97	1.991	1.979	1.968	-11	-23	-34
**15**	0.85	2.013	1.983	1.945	11	-19	-57
**16**	0.94	2.009	1.984	1.926	7	-18	-76
**17**	1.06	1.980	1.977	1.917	-22	-26	-85
**18**	0.99	1.984	1.980	1.940	-18	-22	-62
**19**	0.90	2.019	1.979	1.976	17	-23	-26
**20**	0.89	2.005	1.962	1.962	2	-41	-41
**21**	0.88	2.000	1.969	1.961	-3	-33	-41
**22**	0.84	1.989	1.955	1.941	-13	-47	-62

Table S34. Individual components of the *g*-tensors and *g*-shifts (in parts per thousand) for the Mo-based complexes obtained using the BP86 functional.

	Mo spin pop.	g_{11}	g_{22}	g_{33}	Δg_{11}	Δg_{22}	Δg_{33}
**1**	0.86	2.031	2.030	1.999	29	28	-4
**2**	0.83	2.020	2.019	1.999	17	17	-3
**3**	0.76	2.028	1.993	1.992	26	-10	-10
**4**	0.79	2.026	1.994	1.987	23	-8	-15
**5**	0.79	2.028	1.986	1.967	26	-16	-35
**6**	0.81	2.027	1.988	1.963	24	-14	-39
**7**	0.63	2.031	1.959	1.906	29	-43	-96
**8**	0.85	2.030	1.977	1.975	28	-26	-27
**9**	0.81	2.035	1.979	1.968	33	-23	-34
**10**	0.77	2.007	1.962	1.881	4	-40	-122
**11**	0.87	2.005	1.971	1.959	3	-31	-43

**12**	0.87	2.004	1.954	1.836	2	-49	-166
**13**	0.93	1.972	1.966	1.948	-30	-37	-54
**14**	1.03	1.992	1.980	1.970	-10	-23	-33
**15**	0.91	2.013	1.984	1.947	10	-19	-55
**16**	1.02	2.008	1.985	1.929	6	-18	-73
**17**	1.13	1.980	1.978	1.920	-22	-25	-82
**18**	1.04	1.985	1.981	1.943	-17	-22	-60
**19**	0.93	2.020	1.980	1.977	18	-23	-26
**20**	0.92	2.005	1.962	1.962	3	-40	-40
**21**	0.92	2.000	1.969	1.962	-2	-33	-40
**22**	0.87	1.990	1.956	1.942	-12	-47	-60

Table S35. Individual components of the *g*-tensors and *g*-shifts (in parts per thousand) for the Mo-based complexes obtained using the MN15-L functional.

	**Mo spin pop.**	g_{11}	g_{22}	g_{33}	Δg_{11}	Δg_{22}	Δg_{33}
**1**	1.54	2.013	2.011	1.991	11	9	-11
**2**	1.46	2.005	2.005	1.990	3	2	-13
**3**	1.17	2.021	1.987	1.987	19	-15	-16
**4**	1.25	2.016	1.989	1.983	13	-14	-20
**5**	1.23	2.017	1.981	1.966	15	-21	-36
**6**	1.35	2.012	1.983	1.963	10	-20	-39
**7**	0.89	2.025	1.967	1.919	23	-36	-83
**8**	1.27	2.019	1.974	1.973	17	-29	-30
**9**	1.22	2.024	1.977	1.967	21	-25	-35
**10**	1.10	2.003	1.967	1.895	0	-36	-108
**11**	1.23	1.998	1.971	1.961	-4	-31	-41
**12**	1.21	2.000	1.960	1.863	-3	-42	-140
**13**	1.22	1.972	1.966	1.952	-30	-36	-50
**14**	1.61	1.986	1.977	1.968	-16	-25	-34
**15**	1.55	2.001	1.979	1.949	-1	-23	-53
**16**	1.91	1.991	1.977	1.934	-11	-26	-68
**17**	1.87	1.972	1.970	1.928	-30	-32	-74
**18**	1.55	1.980	1.978	1.946	-22	-24	-56
**19**	1.28	2.007	1.977	1.973	5	-26	-29
**20**	1.20	1.994	1.964	1.964	-8	-39	-39
**21**	1.32	1.989	1.968	1.962	-14	-35	-40
**22**	1.19	1.982	1.958	1.949	-21	-44	-53

Table S36. Individual components of the *g*-tensors and *g*-shifts (in parts per thousand) for the Mo-based complexes obtained using the r²SCAN functional.

	Mo spin pop.	g_{11}	g_{22}	g_{33}	Δg_{11}	Δg_{22}	Δg_{33}
**1**	0.98	2.021	2.020	1.995	19	18	-7
**2**	0.95	2.012	2.012	1.995	10	9	-7
**3**	0.84	2.023	1.991	1.991	21	-11	-11
**4**	0.90	2.020	1.993	1.987	18	-10	-16
**5**	0.86	2.022	1.986	1.970	19	-17	-32
**6**	0.92	2.019	1.987	1.967	17	-15	-36
**7**	0.66	2.027	1.969	1.927	25	-34	-75
**8**	0.92	2.023	1.978	1.977	21	-24	-25
**9**	0.87	2.028	1.981	1.972	26	-22	-31
**10**	0.79	2.005	1.969	1.904	3	-34	-99
**11**	0.93	2.002	1.975	1.965	-1	-28	-37
**12**	0.90	2.003	1.962	1.870	1	-40	-133
**13**	0.99	1.975	1.969	1.955	-28	-33	-47
**14**	1.10	1.989	1.981	1.971	-13	-21	-31
**15**	1.01	2.007	1.983	1.953	5	-19	-49
**16**	1.15	2.001	1.983	1.939	-1	-19	-63
**17**	1.23	1.978	1.978	1.932	-24	-25	-70
**18**	1.10	1.984	1.981	1.949	-18	-21	-53
**19**	0.98	2.013	1.981	1.978	11	-22	-24
**20**	0.97	2.001	1.968	1.968	-1	-34	-34
**21**	0.98	1.996	1.972	1.966	-7	-30	-36
**22**	0.93	1.988	1.962	1.953	-15	-40	-49

Table S37. Individual components of the *g*-tensors and *g*-shifts (in parts per thousand) for the Mo-based complexes obtained using the M06-L functional.

	Mo spin pop.	g_{11}	g_{22}	g_{33}	Δg_{11}	Δg_{22}	Δg_{33}
**1**	1.03	2.018	2.016	1.994	16	14	-8
**2**	0.98	2.009	2.008	1.994	7	6	-8
**3**	0.85	2.021	1.989	1.989	19	-13	-13
**4**	0.92	2.019	1.991	1.985	17	-11	-17
**5**	0.86	2.020	1.983	1.968	18	-19	-35
**6**	0.93	2.017	1.985	1.964	15	-17	-38
**7**	0.65	2.027	1.966	1.920	25	-36	-83
**8**	0.92	2.022	1.976	1.975	19	-26	-28
**9**	0.87	2.026	1.979	1.969	24	-24	-33
**10**	0.77	2.005	1.967	1.898	3	-35	-104
**11**	0.90	2.000	1.973	1.962	-2	-30	-40

**12**	0.89	2.003	1.960	1.865	0	-42	-137
**13**	0.96	1.973	1.967	1.952	-29	-35	-50
**14**	1.09	1.988	1.979	1.969	-15	-23	-33
**15**	0.99	2.006	1.981	1.951	4	-21	-52
**16**	1.15	1.999	1.980	1.935	-3	-22	-67
**17**	1.23	1.975	1.975	1.927	-27	-27	-75
**18**	1.10	1.982	1.980	1.946	-20	-22	-56
**19**	0.98	2.010	1.979	1.976	8	-24	-27
**20**	0.96	1.996	1.965	1.965	-7	-37	-37
**21**	0.95	1.992	1.970	1.964	-10	-32	-38
**22**	0.91	1.984	1.960	1.950	-18	-43	-52

Table S38. Individual components of the *g*-tensors and *g*-shifts (in parts per thousand) for the Mo-based complexes obtained using the TPSS functional.

	**Mo spin pop.**	g_{11}	g_{22}	g_{33}	Δg_{11}	Δg_{22}	Δg_{33}
**1**	0.85	2.029	2.027	1.998	26	25	-4
**2**	0.83	2.018	2.017	1.998	16	15	-4
**3**	0.76	2.026	1.992	1.992	24	-10	-10
**4**	0.80	2.024	1.994	1.987	22	-8	-15
**5**	0.78	2.026	1.986	1.969	24	-16	-34
**6**	0.81	2.024	1.988	1.965	22	-14	-38
**7**	0.62	2.030	1.964	1.918	28	-39	-85
**8**	0.84	2.028	1.978	1.976	25	-24	-26
**9**	0.80	2.033	1.980	1.970	30	-22	-32
**10**	0.75	2.006	1.966	1.893	4	-37	-109
**11**	0.85	2.004	1.973	1.962	2	-29	-40
**12**	0.86	2.004	1.958	1.856	2	-44	-146
**13**	0.92	1.974	1.968	1.952	-28	-34	-50
**14**	1.02	1.991	1.981	1.971	-11	-21	-31
**15**	0.90	2.011	1.984	1.950	9	-18	-52
**16**	1.00	2.007	1.985	1.934	5	-18	-69
**17**	1.12	1.981	1.979	1.927	-21	-23	-75
**18**	1.04	1.985	1.982	1.946	-17	-21	-56
**19**	0.93	2.018	1.980	1.978	16	-22	-25
**20**	0.91	2.005	1.966	1.966	2	-37	-37
**21**	0.90	2.000	1.971	1.965	-3	-31	-37
**22**	0.86	1.990	1.959	1.948	-13	-43	-54

Table S39. Individual components of the g-tensors and g-shifts (in parts per thousand) for the Mo-based complexes obtained using the BLYP functional.

	Mo spin pop.	g_{11}	g_{22}	g_{33}	Δg_{11}	Δg_{22}	Δg_{33}
**1**	0.81	2.031	2.030	1.999	28	27	-3
**2**	0.79	2.019	2.019	2.000	17	16	-3
**3**	0.72	2.028	1.993	1.992	25	-10	-10
**4**	0.75	2.025	1.994	1.986	23	-8	-16
**5**	0.75	2.028	1.986	1.966	26	-16	-36
**6**	0.76	2.027	1.988	1.962	25	-14	-41
**7**	0.61	2.007	1.958	1.902	29	-45	-100
**8**	0.81	2.032	1.976	1.974	28	-26	-28
**9**	0.77	2.035	1.978	1.968	33	-24	-35
**10**	0.74	2.006	1.961	1.877	4	-41	-125
**11**	0.83	2.005	1.970	1.958	2	-32	-45
**12**	0.83	2.004	1.952	1.832	2	-50	-170
**13**	0.90	1.971	1.964	1.946	-31	-38	-56
**14**	0.97	1.991	1.979	1.968	-11	-23	-34
**15**	0.85	2.013	1.983	1.945	11	-19	-57
**16**	0.94	2.009	1.984	1.926	7	-18	-76
**17**	1.06	1.980	1.977	1.917	-22	-26	-85
**18**	0.99	1.984	1.980	1.940	-18	-22	-62
**19**	0.90	2.019	1.979	1.976	17	-23	-26
**20**	0.89	2.005	1.962	1.962	2	-41	-41
**21**	0.88	2.000	1.969	1.961	-3	-33	-41
**22**	0.84	1.989	1.955	1.941	-13	-47	-62

Table S40. Individual components of the g-tensors and g-shifts (in parts per thousand) for the Mo-based complexes obtained using the B3PW91 functional with 20% HFX.

	Mo spin pop.	g_{11}	g_{22}	g_{33}	Δg_{11}	Δg_{22}	Δg_{33}
**1**	0.99	2.020	2.018	1.993	18	16	-9
**2**	0.96	2.011	2.010	1.994	8	8	-9
**3**	0.86	2.026	1.986	1.986	24	-16	-16
**4**	0.93	2.023	1.989	1.981	21	-13	-21
**5**	0.87	2.026	1.980	1.958	23	-23	-44
**6**	0.94	2.023	1.982	1.957	20	-20	-46
**7**	0.64	2.036	1.954	1.895	33	-48	-107
**8**	0.92	2.026	1.971	1.968	24	-31	-35
**9**	0.88	2.032	1.973	1.961	30	-29	-41
**10**	0.77	2.008	1.952	1.846	5	-50	-156
**11**	0.92	1.999	1.966	1.951	-4	-37	-51
**12**	0.89	2.005	1.943	1.800	2	-59	-202
**13**	0.98	1.967	1.958	1.938	-35	-44	-65

**14**	1.10	1.985	1.974	1.962	-18	-28	-41
**15**	1.01	2.009	1.977	1.940	7	-25	-62
**16**	1.15	2.002	1.975	1.920	0	-28	-82
**17**	1.22	1.972	1.969	1.908	-30	-33	-94
**18**	1.09	1.979	1.975	1.933	-24	-27	-69
**19**	0.99	2.012	1.974	1.971	10	-28	-31
**20**	0.97	1.997	1.958	1.958	-6	-44	-44
**21**	0.97	1.991	1.964	1.954	-11	-39	-48
**22**	0.93	1.981	1.950	1.937	-21	-52	-66

Table S41. Individual components of the g-tensors and g-shifts (in parts per thousand) for the Mo-based complexes obtained using the B3PW91 functional with 30% HFX.

	**Mo spin pop.**	g_{11}	g_{22}	g_{33}	Δg_{11}	Δg_{22}	Δg_{33}
**1**	1.06	2.013	2.011	1.989	10	9	-13
**2**	1.02	2.004	2.003	1.990	2	1	-13
**3**	0.91	2.024	1.983	1.983	22	-19	-20
**4**	0.99	2.020	1.986	1.978	18	-16	-24
**5**	0.92	2.023	1.976	1.954	21	-26	-48
**6**	1.00	2.019	1.979	1.953	17	-24	-49
**7**	0.64	2.038	1.952	1.891	36	-50	-111
**8**	0.96	2.023	1.968	1.964	20	-34	-39
**9**	0.92	2.029	1.970	1.958	27	-32	-45
**10**	0.77	2.009	1.947	1.830	6	-55	-173
**11**	0.96	1.995	1.963	1.948	-7	-39	-55
**12**	0.90	2.005	1.938	1.783	3	-64	-219
**13**	1.00	1.965	1.955	1.933	-38	-47	-69
**14**	1.14	1.980	1.972	1.958	-22	-31	-44
**15**	1.06	2.006	1.973	1.937	4	-29	-65
**16**	1.23	1.998	1.969	1.915	-4	-34	-87
**17**	1.28	1.967	1.963	1.902	-35	-39	-100
**18**	1.11	1.975	1.973	1.929	-27	-30	-74
**19**	1.02	2.008	1.972	1.968	5	-31	-34
**20**	1.00	1.992	1.956	1.956	-11	-46	-46
**21**	1.00	1.986	1.961	1.950	-16	-41	-52
**22**	0.97	1.976	1.947	1.935	-26	-55	-68

Table S42. Individual components of the g-tensors and g-shifts (in parts per thousand) for the Mo-based complexes obtained using the B3PW91 functional with 40% HFX.

	**Mo spin pop.**	g_{11}	g_{22}	g_{33}	Δg_{11}	Δg_{22}	Δg_{33}
**1**	1.13	2.005	2.003	1.984	3	0	-18
**2**	1.08	1.997	1.996	1.985	-5	-6	-17

**3**	0.96	2.021	1.979	1.979	19	-23	-23
**4**	1.04	2.016	1.983	1.975	14	-19	-27
**5**	0.97	2.019	1.973	1.950	17	-30	-53
**6**	1.05	2.015	1.975	1.950	12	-27	-52
**7**	0.64	2.040	1.950	1.886	38	-52	-116
**8**	1.00	2.019	1.965	1.960	16	-37	-43
**9**	0.96	2.026	1.967	1.954	23	-35	-49
**10**	0.76	2.009	1.943	1.813	7	-59	-189
**11**	0.99	1.991	1.961	1.944	-12	-42	-59
**12**	0.89	2.006	1.934	1.767	3	-68	-236
**13**	1.02	1.962	1.952	1.928	-40	-50	-74
**14**	1.18	1.976	1.969	1.954	-26	-33	-48
**15**	1.11	2.003	1.970	1.934	0	-32	-68
**16**	1.34	1.993	1.961	1.910	-10	-41	-92
**17**	1.37	1.962	1.956	1.896	-41	-46	-106
**18**	1.14	1.972	1.970	1.925	-30	-33	-78
**19**	1.05	2.003	1.969	1.965	1	-33	-38
**20**	1.03	1.986	1.954	1.954	-16	-48	-48
**21**	1.04	1.980	1.958	1.946	-22	-44	-56
**22**	1.00	1.971	1.945	1.932	-31	-58	-70

Table S43. Individual components of the g-tensors and g-shifts (in parts per thousand) for the Mo-based complexes obtained using the B3PW91 functional with 50% HFX.

	**Mo spin pop.**	g_{11}	g_{22}	g_{33}	Δg_{11}	Δg_{22}	Δg_{33}
**1**	1.19	1.997	1.994	1.977	-5	-9	-25
**2**	1.13	1.990	1.989	1.979	-13	-14	-23
**3**	1.00	2.017	1.975	1.975	15	-27	-27
**4**	1.09	2.011	1.980	1.972	9	-22	-30
**5**	1.02	2.015	1.969	1.945	13	-34	-57
**6**	1.09	2.010	1.972	1.947	8	-31	-55
**7**	0.63	2.043	1.949	1.881	41	-53	-121
**8**	1.05	2.014	1.962	1.956	12	-40	-47
**9**	1.00	2.021	1.964	1.950	19	-38	-52
**10**	0.75	2.011	1.939	1.797	8	-64	-206
**11**	1.02	1.986	1.958	1.940	-16	-44	-63
**12**	0.89	2.006	1.930	1.750	4	-72	-252
**13**	1.04	1.960	1.949	1.923	-43	-54	-79
**14**	1.22	1.972	1.966	1.950	-31	-36	-52
**15**	1.15	1.998	1.967	1.931	-4	-36	-71
**16**	1.47	1.987	1.952	1.904	-16	-50	-98
**17**	1.49	1.956	1.947	1.889	-47	-55	-113
**18**	1.16	1.969	1.967	1.921	-34	-36	-82
**19**	1.08	1.998	1.966	1.961	-4	-36	-41

**20**	1.06	1.981	1.952	1.952	-22	-51	-51
**21**	1.07	1.975	1.955	1.942	-28	-47	-60
**22**	1.04	1.967	1.942	1.930	-36	-60	-72

Table S44. Individual components of the *g*-tensors and *g*-shifts (in parts per thousand) for the Mo-based complexes obtained using the PBE0 functional.

	Mo spin pop.	g_{11}	g_{22}	g_{33}	Δg_{11}	Δg_{22}	Δg_{33}
**1**	1.04	2.016	2.015	1.991	14	12	-11
**2**	1.00	2.007	2.007	1.991	5	4	-11
**3**	0.89	2.025	1.985	1.984	23	-18	-18
**4**	0.97	2.021	1.988	1.980	19	-15	-22
**5**	0.91	2.024	1.978	1.956	21	-24	-46
**6**	0.98	2.020	1.980	1.956	18	-22	-47
**7**	0.65	2.036	1.954	1.895	34	-49	-108
**8**	0.95	2.024	1.970	1.966	22	-33	-36
**9**	0.91	2.030	1.972	1.960	28	-30	-43
**10**	0.78	2.008	1.951	1.841	6	-52	-162
**11**	0.95	1.997	1.965	1.950	-6	-37	-52
**12**	0.90	2.005	1.942	1.795	2	-61	-207
**13**	0.90	1.966	1.957	1.936	-36	-45	-66
**14**	1.13	1.983	1.973	1.960	-20	-29	-42
**15**	1.05	2.007	1.975	1.940	5	-27	-63
**16**	1.21	2.000	1.972	1.919	-3	-30	-84
**17**	1.27	1.970	1.967	1.906	-33	-36	-96
**18**	1.11	1.977	1.974	1.932	-25	-28	-71
**19**	1.01	2.010	1.973	1.970	7	-29	-33
**20**	0.99	1.994	1.958	1.958	-8	-45	-45
**21**	1.00	1.988	1.963	1.953	-14	-40	-50
**22**	0.96	1.979	1.949	1.937	-24	-53	-66

Table S45. Individual components of the *g*-tensors and *g*-shifts (in parts per thousand) for the Mo-based complexes obtained using the TPSSh functional.

	Mo spin pop.	g_{11}	g_{22}	g_{33}	Δg_{11}	Δg_{22}	Δg_{33}
**1**	0.93	2.024	2.022	1.995	21	20	-7
**2**	0.90	2.014	2.013	1.996	12	11	-7
**3**	0.81	2.025	1.989	1.989	23	-13	-13
**4**	0.88	2.023	1.991	1.984	21	-11	-18
**5**	0.82	2.025	1.984	1.965	22	-19	-38
**6**	0.88	2.023	1.985	1.962	20	-17	-40
**7**	0.63	2.032	1.961	1.913	30	-41	-89
**8**	0.88	2.026	1.975	1.973	24	-27	-29

**9**	0.84	2.031	1.977	1.967	29	-25	-36
**10**	0.76	2.007	1.961	1.879	5	-41	-123
**11**	0.89	2.002	1.971	1.959	-1	-32	-44
**12**	0.88	2.004	1.954	1.842	2	-49	-161
**13**	0.95	1.972	1.965	1.948	-31	-38	-55
**14**	1.06	1.988	1.978	1.967	-15	-24	-35
**15**	0.96	2.010	1.981	1.947	8	-21	-55
**16**	1.08	2.004	1.980	1.930	2	-22	-73
**17**	1.17	1.977	1.975	1.922	-25	-27	-81
**18**	1.07	1.982	1.979	1.942	-20	-23	-60
**19**	0.96	2.014	1.978	1.975	12	-24	-27
**20**	0.94	2.001	1.964	1.964	-2	-39	-39
**21**	0.93	1.995	1.969	1.961	-7	-34	-41
**22**	0.90	1.986	1.957	1.946	-17	-46	-57

Table S46. Individual components of the *g*-tensors and *g*-shifts (in parts per thousand) for the Mo-based complexes obtained using the B3LYP functional.

	**Mo spin pop.**	g_{11}	g_{22}	g_{33}	Δg_{11}	Δg_{22}	Δg_{33}
**1**	0.95	2.019	2.018	1.994	17	16	-9
**2**	0.92	2.010	2.009	1.994	8	7	-8
**3**	0.82	2.026	1.987	1.986	24	-16	-16
**4**	0.89	2.023	1.989	1.981	21	-13	-22
**5**	0.84	2.026	1.980	1.957	24	-23	-45
**6**	0.89	2.023	1.982	1.955	21	-20	-47
**7**	0.63	2.036	1.953	1.893	34	-49	-110
**8**	0.89	2.026	1.971	1.967	24	-32	-35
**9**	0.85	2.033	1.973	1.960	30	-30	-42
**10**	0.75	2.008	1.951	1.844	5	-51	-159
**11**	0.89	1.999	1.965	1.951	-4	-37	-52
**12**	0.86	2.005	1.942	1.798	2	-60	-205
**13**	0.95	1.966	1.957	1.936	-36	-45	-66
**14**	1.05	1.984	1.974	1.961	-18	-28	-42
**15**	0.96	2.010	1.976	1.938	8	-26	-64
**16**	1.08	2.004	1.974	1.917	1	-28	-85
**17**	1.15	1.972	1.968	1.906	-31	-34	-97
**18**	1.04	1.978	1.975	1.931	-24	-28	-71
**19**	0.96	2.011	1.974	1.970	9	-28	-32
**20**	0.95	1.996	1.958	1.958	-6	-45	-45
**21**	0.94	1.991	1.963	1.953	-12	-39	-49
**22**	0.91	1.980	1.949	1.936	-22	-53	-67

Table S47. Individual components of the *g*-tensors and *g*-shifts (in parts per thousand) for the Mo-based complexes obtained using the M05 functional.

	Mo spin pop.	g_{11}	g_{22}	g_{33}	Δg_{11}	Δg_{22}	Δg_{33}
**1**	0.99	2.013	2.010	1.989	11	8	-13
**2**	0.96	2.002	2.000	1.990	-1	-2	-13
**3**	0.87	2.023	1.977	1.976	21	-25	-26
**4**	0.94	2.022	1.983	1.974	19	-19	-28
**5**	0.88	2.022	1.968	1.940	20	-35	-62
**6**	0.97	2.020	1.973	1.942	17	-29	-61
**7**	0.64	2.037	1.924	1.819	35	-79	-183
**8**	0.92	2.022	1.958	1.953	20	-44	-49
**9**	0.87	2.029	1.961	1.945	27	-41	-57
**10**	0.79	2.008	1.928	1.745	5	-75	-257
**11**	0.92	1.989	1.951	1.927	-13	-51	-75
**12**	0.95	2.003	1.912	1.670	1	-91	-332
**13**	1.00	1.954	1.941	1.909	-48	-61	-93
**14**	1.18	1.975	1.963	1.947	-28	-40	-55
**15**	1.09	2.005	1.966	1.920	3	-36	-82
**16**	1.23	1.995	1.959	1.890	-7	-44	-113
**17**	1.30	1.959	1.952	1.859	-44	-51	-143
**18**	1.16	1.968	1.965	1.904	-34	-38	-98
**19**	0.99	2.004	1.965	1.959	1	-37	-44
**20**	0.97	1.978	1.941	1.941	-24	-62	-62
**21**	0.99	1.975	1.950	1.931	-27	-52	-71
**22**	0.95	1.963	1.929	1.905	-39	-73	-97

Table S48. Individual components of the *g*-tensors and *g*-shifts (in parts per thousand) for the Mo-based complexes obtained using the M06-2X functional.

	Mo spin pop.	g_{11}	g_{22}	g_{33}	Δg_{11}	Δg_{22}	Δg_{33}
**1**	1.24	1.993	1.988	1.972	-9	-14	-30
**2**	1.18	1.984	1.983	1.975	-18	-20	-27
**3**	1.02	2.020	1.969	1.968	18	-33	-34
**4**	1.09	2.014	1.975	1.967	11	-27	-36
**5**	1.01	2.017	1.961	1.930	15	-42	-72
**6**	1.07	2.012	1.965	1.934	10	-37	-68
**7**	0.67	2.055	1.925	1.809	53	-77	-193
**8**	1.03	2.015	1.952	1.943	13	-50	-59
**9**	0.99	2.024	1.955	1.936	22	-47	-67
**10**	0.77	2.012	1.911	1.640	10	-91	-362
**11**	0.99	1.981	1.946	1.918	-21	-57	-85
**12**	0.86	2.007	1.892	1.545	5	-110	-457
**13**	0.99	1.949	1.933	1.893	-54	-70	-109

**14**	1.14	1.967	1.958	1.940	-35	-44	-62
**15**	1.08	1.999	1.959	1.914	-3	-44	-88
**16**	1.34	1.985	1.940	1.874	-17	-62	-129
**17**	1.33	1.949	1.936	1.846	-53	-67	-157
**18**	1.09	1.963	1.959	1.897	-39	-43	-106
**19**	1.07	1.996	1.958	1.951	-6	-44	-52
**20**	1.05	1.968	1.937	1.937	-34	-66	-66
**21**	1.00	1.965	1.944	1.922	-37	-58	-80
**22**	0.99	1.954	1.924	1.902	-49	-79	-101

Table S49. Individual components of the *g*-tensors and *g*-shifts (in parts per thousand) for the Mo-based complexes obtained using the MN15 functional.

	**Mo spin pop.**	g_{11}	g_{22}	g_{33}	Δg_{11}	Δg_{22}	Δg_{33}
**1**	1.19	2.004	2.001	1.984	2	-2	-18
**2**	1.14	1.991	1.989	1.985	-11	-13	-17
**3**	1.01	2.028	1.969	1.968	26	-33	-34
**4**	1.09	2.022	1.977	1.966	20	-25	-37
**5**	1.02	2.028	1.959	1.919	25	-43	-83
**6**	1.11	2.022	1.966	1.927	20	-36	-75
**7**	0.66	2.056	1.891	1.704	53	-111	-299
**8**	1.05	2.024	1.947	1.937	22	-55	-65
**9**	1.01	2.034	1.950	1.926	32	-52	-76
**10**	0.85	2.014	1.883	1.505	11	-119	-497
**11**	1.02	1.985	1.937	1.902	-18	-65	-100
**12**	0.97	2.008	1.859	1.393	6	-143	-609
**13**	1.04	1.944	1.924	1.877	-59	-78	-125
**14**	1.28	1.971	1.956	1.936	-32	-46	-66
**15**	1.21	2.007	1.958	1.903	5	-45	-99
**16**	1.43	1.994	1.946	1.862	-8	-56	-140
**17**	1.44	1.952	1.942	1.834	-51	-61	-168
**18**	1.23	1.964	1.959	1.886	-39	-44	-116
**19**	1.09	2.000	1.954	1.947	-2	-48	-56
**20**	1.05	1.970	1.924	1.924	-33	-78	-78
**21**	1.08	1.969	1.937	1.911	-34	-66	-92
**22**	1.01	1.952	1.907	1.873	-50	-96	-129

Table S50. Individual components of the *g*-tensors and *g*-shifts (in parts per thousand) for the Mo-based complexes obtained using the M06 functional.

	**Mo spin pop.**	g_{11}	g_{22}	g_{33}	Δg_{11}	Δg_{22}	Δg_{33}
**1**	0.99	2.013	2.011	1.990	11	9	-12
**2**	0.96	2.003	2.002	1.990	0	-1	-12

**3**	0.92	2.026	1.980	1.980	24	-22	-23
**4**	0.95	2.023	1.985	1.976	20	-18	-27
**5**	0.91	2.025	1.971	1.945	23	-31	-58
**6**	0.99	2.022	1.975	1.945	20	-27	-58
**7**	0.67	2.039	1.932	1.839	37	-70	-164
**8**	0.92	2.025	1.961	1.957	23	-41	-46
**9**	0.88	2.032	1.964	1.948	30	-38	-54
**10**	0.83	2.008	1.934	1.776	5	-68	-227
**11**	0.93	1.992	1.955	1.934	-10	-48	-68
**12**	1.02	2.004	1.922	1.718	1	-80	-284
**13**	1.02	1.958	1.946	1.917	-44	-56	-86
**14**	1.40	1.978	1.966	1.952	-24	-36	-50
**15**	1.23	2.007	1.969	1.926	5	-33	-76
**16**	1.40	1.997	1.963	1.898	-5	-39	-104
**17**	1.54	1.962	1.957	1.879	-40	-45	-123
**18**	1.39	1.971	1.968	1.915	-31	-34	-87
**19**	1.00	2.007	1.967	1.962	4	-35	-40
**20**	0.99	1.985	1.946	1.946	-17	-56	-56
**21**	1.02	1.982	1.953	1.938	-21	-49	-64
**22**	0.95	1.970	1.935	1.914	-33	-68	-88

Table S51. Individual components of the *g*-tensors and *g*-shifts (in parts per thousand) for the Mo-based complexes obtained using the ωB97 functional.

	**Mo spin pop.**	g_{11}	g_{22}	g_{33}	Δg_{11}	Δg_{22}	Δg_{33}
**1**	1.15	2.001	1.997	1.984	-1	-6	-18
**2**	1.10	1.993	1.992	1.986	-9	-10	-16
**3**	0.97	2.019	1.978	1.978	17	-24	-24
**4**	1.03	2.015	1.982	1.976	13	-20	-26
**5**	0.97	2.018	1.972	1.949	16	-30	-53
**6**	1.03	2.014	1.975	1.952	11	-27	-50
**7**	0.56	2.050	1.951	1.891	47	-51	-112
**8**	0.99	2.017	1.966	1.959	14	-37	-44
**9**	0.95	2.024	1.968	1.954	21	-35	-48
**10**	0.74	2.009	1.942	1.813	7	-60	-189
**11**	0.96	1.990	1.961	1.943	-12	-41	-59
**12**	0.85	2.006	1.934	1.776	3	-69	-226
**13**	0.95	1.964	1.953	1.930	-39	-49	-73
**14**	1.09	1.977	1.969	1.956	-25	-33	-46
**15**	1.05	2.004	1.969	1.938	1	-33	-64
**16**	1.36	1.997	1.957	1.914	-6	-46	-88
**17**	1.32	1.965	1.953	1.897	-37	-49	-105
**18**	1.03	1.975	1.970	1.927	-28	-32	-76
**19**	1.01	2.002	1.970	1.966	-1	-32	-36

**20**	1.00	1.984	1.956	1.956	-18	-46	-46
**21**	0.99	1.980	1.960	1.947	-22	-42	-55
**22**	0.95	1.970	1.947	1.934	-32	-55	-68

Table S52. Individual components of the *g*-tensors and *g*-shifts (in parts per thousand) for the Mo-based complexes obtained using the CAM-B3LYP functional.

	**Mo spin pop.**	g_{11}	g_{22}	g_{33}	Δg_{11}	Δg_{22}	Δg_{33}
**1**	1.03	2.010	2.008	1.989	8	6	-13
**2**	0.99	2.002	2.001	1.990	0	-1	-12
**3**	0.90	2.024	1.982	1.982	22	-20	-21
**4**	0.97	2.020	1.985	1.978	18	-17	-25
**5**	0.90	2.024	1.976	1.952	22	-27	-50
**6**	0.97	2.020	1.978	1.953	17	-24	-50
**7**	0.60	2.044	1.951	1.890	41	-51	-113
**8**	0.93	2.023	1.967	1.962	21	-35	-40
**9**	0.89	2.030	1.970	1.956	28	-33	-46
**10**	0.74	2.009	1.945	1.823	7	-58	-179
**11**	0.93	1.994	1.962	1.946	-8	-40	-56
**12**	0.86	2.006	1.936	1.780	3	-66	-222
**13**	0.96	1.964	1.954	1.931	-38	-48	-71
**14**	1.09	1.980	1.971	1.957	-23	-31	-45
**15**	1.02	2.008	1.972	1.937	5	-30	-65
**16**	1.17	2.001	1.967	1.915	-2	-36	-88
**17**	1.21	1.968	1.961	1.900	-34	-41	-102
**18**	1.05	1.975	1.972	1.927	-27	-31	-75
**19**	0.99	2.007	1.971	1.968	4	-31	-35
**20**	0.98	1.991	1.956	1.956	-12	-46	-46
**21**	0.96	1.986	1.961	1.949	-17	-42	-53
**22**	0.94	1.975	1.947	1.934	-27	-55	-69

Table S53. Individual components of the *g*-tensors and *g*-shifts (in parts per thousand) for the Mo-based complexes obtained using the LC-PBE functional.

	**Mo spin pop.**	g_{11}	g_{22}	g_{33}	Δg_{11}	Δg_{22}	Δg_{33}
**1**	1.14	2.000	1.995	1.982	-2	-7	-20
**2**	1.10	1.993	1.991	1.984	-10	-11	-18
**3**	1.01	2.020	1.977	1.977	18	-25	-26
**4**	1.08	2.015	1.982	1.975	13	-21	-27
**5**	1.01	2.018	1.971	1.949	16	-31	-54
**6**	1.07	2.013	1.975	1.952	11	-28	-50
**7**	0.55	2.056	1.950	1.888	53	-53	-114
**8**	1.02	2.017	1.965	1.958	14	-37	-45

**9**	0.98	2.024	1.967	1.953	22	-35	-49
**10**	0.75	2.010	1.938	1.804	8	-64	-198
**11**	0.99	1.990	1.961	1.942	-13	-42	-60
**12**	0.88	2.006	1.930	1.765	4	-73	-237
**13**	1.00	1.964	1.953	1.928	-39	-50	-74
**14**	1.16	1.976	1.969	1.956	-26	-33	-46
**15**	1.11	2.002	1.969	1.939	0	-33	-63
**16**	1.50	1.994	1.954	1.913	-8	-49	-89
**17**	1.49	1.962	1.950	1.895	-40	-53	-107
**18**	1.10	1.974	1.970	1.928	-28	-32	-75
**19**	1.05	2.002	1.969	1.966	0	-33	-37
**20**	1.03	1.986	1.955	1.955	-17	-47	-47
**21**	1.02	1.981	1.959	1.946	-21	-43	-57
**22**	0.99	1.971	1.946	1.933	-31	-56	-69

Table S54. Individual components of the *g*-tensors and *g*-shifts (in parts per thousand) for the Mo-based complexes obtained using the LC-BLYP functional.

	**Mo spin pop.**	g_{11}	g_{22}	g_{33}	Δg_{11}	Δg_{22}	Δg_{33}
**1**	0.99	2.014	2.011	1.992	12	9	-10
**2**	0.96	2.006	2.005	1.993	4	3	-9
**3**	0.88	2.027	1.983	1.983	24	-19	-19
**4**	0.95	2.023	1.986	1.980	21	-16	-23
**5**	0.88	2.027	1.977	1.953	24	-25	-49
**6**	0.94	2.023	1.980	1.955	21	-23	-47
**7**	0.57	2.047	1.951	1.892	45	-51	-110
**8**	0.91	2.026	1.969	1.963	23	-33	-39
**9**	0.87	2.032	1.971	1.958	30	-31	-44
**10**	0.73	2.009	1.945	1.827	7	-58	-176
**11**	0.90	1.997	1.963	1.947	-6	-39	-55
**12**	0.84	2.006	1.936	1.786	3	-66	-216
**13**	0.93	1.966	1.956	1.933	-36	-46	-69
**14**	1.05	1.983	1.972	1.960	-20	-30	-42
**15**	0.98	2.011	1.973	1.941	9	-29	-61
**16**	1.12	2.006	1.968	1.920	4	-34	-82
**17**	1.16	1.973	1.963	1.905	-29	-39	-98
**18**	1.01	1.978	1.973	1.931	-24	-29	-71
**19**	0.96	2.010	1.973	1.970	7	-29	-32
**20**	0.95	1.994	1.958	1.958	-8	-44	-44
**21**	0.93	1.989	1.962	1.950	-13	-40	-52
**22**	0.91	1.978	1.949	1.935	-24	-54	-67

Table S55. Individual components of the g-tensors and g-shifts (in parts per thousand) for the Mo-based complexes obtained using the PBE0-DH functional.

	Mo spin pop.	g_{11}	g_{22}	g_{33}	Δg_{11}	Δg_{22}	Δg_{33}
**1**	0.96	2.018	2.017	2.001	15	15	-1
**2**	0.95	2.009	2.008	1.999	6	6	-3
**3**	0.90	2.022	1.983	1.983	20	-19	-19
**4**	0.98	2.018	1.987	1.979	16	-16	-23
**5**	0.89	2.021	1.977	1.954	19	-25	-48
**6**	0.96	2.017	1.980	1.955	15	-23	-48
**7**	0.68	2.031	1.952	1.890	29	-51	-112
**8**	0.93	2.020	1.969	1.965	18	-34	-37
**9**	0.89	2.027	1.971	1.959	25	-31	-44
**10**	0.80	2.006	1.949	1.822	4	-53	-180
**11**	0.93	1.992	1.964	1.947	-10	-39	-55
**12**	0.91	2.003	1.940	1.776	1	-62	-226
**13**	0.98	1.965	1.954	1.931	-38	-48	-71
**14**	1.08	1.979	1.973	1.959	-23	-29	-43
**15**	1.02	2.005	1.975	1.938	3	-28	-64
**16**	0.93	2.008	1.980	1.917	6	-22	-85
**17**	1.02	1.976	1.973	1.903	-26	-29	-100
**18**	1.06	1.975	1.974	1.928	-27	-29	-74
**19**	1.01	2.004	1.972	1.968	2	-30	-34
**20**	1.00	1.986	1.957	1.957	-17	-46	-46
**21**	0.97	1.983	1.963	1.950	-20	-40	-52
**22**	0.94	1.972	1.950	1.934	-30	-53	-68

Table S56. Individual components of the g-tensors and g-shifts (in parts per thousand) for the Mo-based complexes obtained using the ωB2PLYP functional.

	Mo spin pop.	g_{11}	g_{22}	g_{33}	Δg_{11}	Δg_{22}	Δg_{33}
**1**	0.92	2.018	2.018	2.010	16	15	8
**2**	0.92	2.009	2.008	2.004	6	6	2
**3**	0.88	2.022	1.983	1.983	20	-19	-20
**4**	0.95	2.018	1.987	1.979	16	-16	-23
**5**	0.86	2.023	1.977	1.952	20	-25	-50
**6**	0.92	2.018	1.980	1.954	16	-22	-48
**7**	0.72	2.023	1.946	1.884	21	-56	-118
**8**	0.88	2.020	1.969	1.964	18	-34	-39
**9**	0.84	2.028	1.971	1.958	26	-31	-44
**10**	0.79	2.004	1.946	1.799	2	-57	-203
**11**	0.88	1.991	1.963	1.945	-11	-39	-58
**12**	0.88	2.003	1.936	1.754	0	-66	-248
**13**	0.94	1.964	1.953	1.927	-38	-49	-76

**14**	0.97	1.980	1.975	1.958	-22	-27	-44
**15**	0.94	2.007	1.975	1.938	5	-28	-65
**16**	0.68	2.038	1.978	1.912	35	-25	-91
**17**	0.80	1.996	1.971	1.894	-7	-32	-108
**18**	0.98	1.976	1.974	1.925	-26	-28	-78
**19**	0.97	2.002	1.973	1.968	0	-30	-34
**20**	0.96	1.982	1.957	1.957	-21	-45	-45
**21**	0.89	1.983	1.964	1.947	-19	-39	-55
**22**	0.88	1.969	1.952	1.931	-33	-51	-71

Table S57. Individual components of the *g*-tensors and *g*-shifts (in parts per thousand) for the Mo-based complexes obtained using the Pr^{2}SCAN50 functional.

	**Mo spin pop.**	g_{11}	g_{22}	g_{33}	Δg_{11}	Δg_{22}	Δg_{33}
**1**	0.66	2.033	2.030	2.025	30	28	22
**2**	0.74	2.020	2.020	2.018	18	18	16
**3**	0.80	2.023	1.989	1.989	21	-13	-14
**4**	0.86	2.020	1.992	1.985	18	-11	-18
**5**	0.75	2.024	1.985	1.963	21	-17	-39
**6**	0.80	2.020	1.987	1.962	17	-15	-40
**7**	0.68	2.027	1.955	1.904	24	-48	-99
**8**	0.78	2.023	1.976	1.973	20	-26	-29
**9**	0.75	2.029	1.979	1.967	27	-24	-35
**10**	0.85	2.003	1.960	1.854	1	-42	-148
**11**	0.79	1.997	1.972	1.956	-5	-31	-46
**12**	0.87	2.008	1.951	1.529	6	-51	-473
**13**	0.89	1.972	1.962	1.941	-30	-40	-61
**14**	0.83	1.987	1.983	1.966	-16	-19	-36
**15**	0.83	2.010	1.983	1.946	8	-20	-56
**16**	0.33	2.025	2.002	1.920	23	0	-83
**17**	0.51	1.994	1.992	1.904	-8	-11	-98
**18**	0.89	1.982	1.982	1.936	-20	-21	-67
**19**	0.89	2.006	1.979	1.975	4	-24	-27
**20**	0.88	1.989	1.964	1.964	-13	-38	-38
**21**	0.78	1.990	1.972	1.959	-12	-31	-44
**22**	0.78	1.978	1.961	1.943	-25	-41	-60

Table S58. Individual components of the *g*-tensors and *g*-shifts (in parts per thousand) for the Mo-based complexes obtained using the ωB88PP86 functional.

	Mo spin pop.	g_{11}	g_{22}	g_{33}	Δg_{11}	Δg_{22}	Δg_{33}
**1**	0.74	2.047	2.038	2.036	44	36	33
**2**	0.81	2.030	2.025	2.023	27	23	21
**3**	0.83	2.027	1.990	1.989	24	-13	-13
**4**	0.90	2.023	1.993	1.985	20	-9	-17
**5**	0.74	2.028	1.986	1.959	26	-17	-44
**6**	0.80	2.023	1.990	1.959	21	-13	-43
**7**	0.80	2.008	1.946	1.889	6	-57	-114
**8**	0.77	2.026	1.975	1.972	24	-27	-30
**9**	0.73	2.034	1.978	1.965	31	-24	-37
**10**	0.89	1.999	1.952	1.802	-3	-50	-200
**11**	0.77	1.997	1.970	1.949	-5	-32	-53
**12**	0.93	2.001	1.943	1.757	-2	-60	-245
**13**	0.90	1.970	1.958	1.931	-33	-45	-71
**14**	0.76	1.991	1.986	1.963	-11	-16	-39
**15**	0.80	2.015	1.983	1.941	12	-19	-62
**16**	0.67	2.052	1.988	1.902	49	-14	-100
**17**	0.87	2.008	1.978	1.882	6	-25	-120
**18**	0.88	1.984	1.982	1.927	-18	-20	-75
**19**	0.92	2.006	1.979	1.974	4	-24	-28
**20**	0.90	1.985	1.963	1.963	-17	-39	-39
**21**	0.75	1.994	1.972	1.951	-8	-31	-51
**22**	0.74	1.975	1.963	1.930	-27	-39	-72

Table S59. Individual components of the *g*-tensors and *g*-shifts (in parts per thousand) for the Mo-based complexes obtained using the B2GP-PLYP functional.

	Mo spin pop.	g_{11}	g_{22}	g_{33}	Δg_{11}	Δg_{22}	Δg_{33}
**1**	0.70	2.050	2.041	2.037	47	39	35
**2**	0.77	2.031	2.027	2.026	28	24	24
**3**	0.80	2.027	1.990	1.990	25	-12	-12
**4**	0.88	2.023	1.993	1.984	21	-9	-18
**5**	0.74	2.029	1.986	1.959	27	-17	-43
**6**	0.81	2.024	1.989	1.958	22	-14	-44
**7**	0.78	2.007	1.946	1.885	5	-56	-118
**8**	0.77	2.026	1.975	1.972	24	-27	-30
**9**	0.73	2.034	1.978	1.965	31	-24	-37
**10**	0.87	1.998	1.953	1.809	-4	-49	-193
**11**	0.79	1.997	1.970	1.950	-5	-33	-52
**12**	0.91	2.000	1.943	1.763	-2	-59	-239
**13**	0.90	1.969	1.957	1.931	-33	-45	-71

**14**	0.82	1.989	1.984	1.963	-14	-18	-39
**15**	0.83	2.014	1.982	1.939	12	-20	-63
**16**	0.15	2.068	2.002	1.906	66	0	-96
**17**	0.39	2.020	1.991	1.888	18	-11	-115
**18**	0.90	1.982	1.980	1.927	-20	-22	-76
**19**	0.92	2.006	1.978	1.974	4	-24	-29
**20**	0.90	1.985	1.962	1.962	-17	-40	-40
**21**	0.78	1.992	1.970	1.952	-10	-32	-50
**22**	0.77	1.974	1.961	1.931	-28	-42	-71

Table S60. Individual components of the *g*-tensors and *g*-shifts (in parts per thousand) for the Mo-based complexes obtained using the B2PLYP functional.

	**Mo spin pop.**	g_{11}	g_{22}	g_{33}	Δg_{11}	Δg_{22}	Δg_{33}
**1**	0.72	2.041	2.038	2.030	39	36	28
**2**	0.76	2.027	2.027	2.022	24	24	19
**3**	0.77	2.028	1.991	1.991	25	-11	-12
**4**	0.85	2.025	1.993	1.984	23	-9	-18
**5**	0.75	2.029	1.985	1.961	27	-17	-42
**6**	0.82	2.026	1.987	1.959	23	-15	-43
**7**	0.70	2.018	1.951	1.889	15	-52	-114
**8**	0.79	2.027	1.975	1.972	25	-28	-30
**9**	0.76	2.034	1.977	1.965	32	-25	-37
**10**	0.84	2.001	1.955	1.829	-1	-47	-173
**11**	0.82	1.998	1.968	1.952	-4	-34	-51
**12**	0.90	2.001	1.945	1.782	-2	-57	-220
**13**	0.91	1.968	1.958	1.935	-34	-45	-68
**14**	0.92	1.986	1.980	1.965	-16	-22	-38
**15**	0.86	2.014	1.981	1.941	12	-21	-62
**16**	0.27	2.047	2.011	1.921	45	9	-81
**17**	0.41	2.011	2.001	1.906	8	-2	-96
**18**	0.95	1.981	1.979	1.930	-22	-23	-72
**19**	0.92	2.009	1.978	1.973	6	-25	-29
**20**	0.91	1.989	1.961	1.961	-13	-41	-41
**21**	0.84	1.990	1.969	1.955	-12	-34	-47
**22**	0.83	1.976	1.956	1.935	-26	-46	-67

Table S61. Expectation values of $\langle S^2 \rangle$ for the Mo-based complexes calculated using different functionals.

	BP86	BLYP	TPSS	M06-L	MN15-L	r^{2}SCAN	TPSSh	B3LYP	PBE0
**1**	0.7577	0.7557	0.7585	0.7725	0.8159	0.7667	0.7623	0.7623	0.7686
**2**	0.7569	0.7552	0.7577	0.7686	0.8047	0.7645	0.7608	0.7606	0.7656
**3**	0.7539	0.7530	0.7543	0.7575	0.7704	0.7574	0.7560	0.7561	0.7584
**4**	0.7541	0.7530	0.7546	0.7580	0.7723	0.7576	0.7563	0.7563	0.7587
**5**	0.7546	0.7535	0.7550	0.7591	0.7751	0.7589	0.7570	0.7569	0.7597
**6**	0.7542	0.7531	0.7546	0.7588	0.7770	0.7580	0.7563	0.7564	0.7590
**7**	0.7543	0.7536	0.7553	0.7569	0.7650	0.7583	0.7577	0.7580	0.7608
**8**	0.7548	0.7536	0.7551	0.7593	0.7764	0.7589	0.7568	0.7567	0.7595
**9**	0.7544	0.7533	0.7547	0.7586	0.7740	0.7584	0.7564	0.7563	0.7589
**10**	0.7523	0.7518	0.7526	0.7534	0.7603	0.7540	0.7536	0.7537	0.7547
**11**	0.7542	0.7532	0.7544	0.7581	0.7733	0.7576	0.7558	0.7557	0.7581
**12**	0.7523	0.7517	0.7522	0.7534	0.7628	0.7534	0.7525	0.7524	0.7531
**13**	0.7539	0.7529	0.7538	0.7573	0.7703	0.7566	0.7548	0.7546	0.7565
**14**	0.7557	0.7540	0.7554	0.7612	0.7868	0.7594	0.7571	0.7570	0.7606
**15**	0.7543	0.7531	0.7544	0.7594	0.7814	0.7581	0.7560	0.7560	0.7589
**16**	0.7594	0.7564	0.7593	0.7747	0.8400	0.7703	0.7640	0.7648	0.7766
**17**	0.7632	0.7590	0.7625	0.7793	0.8480	0.7752	0.7680	0.7690	0.7834
**18**	0.7553	0.7537	0.7550	0.7602	0.7817	0.7585	0.7562	0.7558	0.7586
**19**	0.7548	0.7535	0.7547	0.7584	0.7756	0.7577	0.7558	0.7554	0.7577
**20**	0.7543	0.7532	0.7542	0.7576	0.7731	0.7570	0.7553	0.7552	0.7575
**21**	0.7547	0.7535	0.7547	0.7594	0.7790	0.7583	0.7561	0.7561	0.7588
**22**	0.7541	0.7531	0.7542	0.7583	0.7739	0.7574	0.7556	0.7556	0.7581

	B3PW91 20% HFX	B3PW91 30% HFX	B3PW91 40% HFX	B3PW91 50% HFX	M05	M06	M06-2X	MN15	ωB97
**1**	0.7649	0.7704	0.7769	0.7844	0.7676	0.7676	0.7916	0.7726	0.7727
**2**	0.7627	0.7668	0.7712	0.7759	0.7653	0.7649	0.7820	0.7682	0.7679
**3**	0.7571	0.7591	0.7612	0.7634	0.7583	0.7582	0.7621	0.7590	0.7588
**4**	0.7574	0.7592	0.7611	0.7630	0.7585	0.7586	0.7629	0.7599	0.7583
**5**	0.7581	0.7605	0.7633	0.7668	0.7612	0.7601	0.7633	0.7602	0.7605
**6**	0.7575	0.7597	0.7622	0.7653	0.7594	0.7595	0.7633	0.7602	0.7595
**7**	0.7588	0.7619	0.7656	0.7700	0.7582	0.7574	0.7624	0.7595	0.7670
**8**	0.7579	0.7603	0.7631	0.7667	0.7613	0.7599	0.7632	0.7603	0.7603
**9**	0.7574	0.7597	0.7624	0.7658	0.7605	0.7592	0.7622	0.7594	0.7600
**10**	0.7539	0.7554	0.7573	0.7598	0.7531	0.7535	0.7559	0.7543	0.7552
**11**	0.7568	0.7587	0.7612	0.7644	0.7591	0.7586	0.7607	0.7584	0.7582
**12**	0.7527	0.7532	0.7538	0.7546	0.7531	0.7540	0.7537	0.7532	0.7528
**13**	0.7555	0.7568	0.7584	0.7604	0.7577	0.7577	0.7578	0.7564	0.7559
**14**	0.7588	0.7615	0.7652	0.7706	0.7621	0.7651	0.7651	0.7629	0.7600
**15**	0.7574	0.7596	0.7623	0.7660	0.7601	0.7613	0.7629	0.7604	0.7591
**16**	0.7694	0.7813	0.8042	0.8511	0.7753	0.7782	0.8133	0.7862	0.8339
**17**	0.7749	0.7891	0.8155	0.8667	0.7809	0.7869	0.8204	0.7958	0.8343
**18**	0.7573	0.7591	0.7615	0.7647	0.7596	0.7626	0.7609	0.7595	0.7569
**19**	0.7567	0.7581	0.7597	0.7616	0.7582	0.7582	0.7609	0.7589	0.7568

**20**	0.7563	0.7579	0.7598	0.7621	0.7578	0.7576	0.7600	0.7580	0.7570
**21**	0.7573	0.7595	0.7625	0.7667	0.7596	0.7598	0.7620	0.7592	0.7587
**22**	0.7567	0.7588	0.7616	0.7653	0.7588	0.7587	0.7610	0.7582	0.7580

	**CAM-B3LYP**	**LC-BLYP**	**LC-PBE**
**1**	0.7661	0.7624	0.7738
**2**	0.7632	0.7604	0.7688
**3**	0.7578	0.7569	0.7606
**4**	0.7575	0.7564	0.7598
**5**	0.7589	0.7576	0.7626
**6**	0.7579	0.7567	0.7609
**7**	0.7622	0.7616	0.7702
**8**	0.7585	0.7572	0.7624
**9**	0.7582	0.7571	0.7622
**10**	0.7551	0.7541	0.7567
**11**	0.7570	0.7557	0.7598
**12**	0.7527	0.7521	0.7533
**13**	0.7551	0.7540	0.7569
**14**	0.7583	0.7562	0.7616
**15**	0.7574	0.7560	0.7604
**16**	0.7774	0.7719	0.8841
**17**	0.7836	0.7764	0.8933
**18**	0.7562	0.7544	0.7581
**19**	0.7560	0.7547	0.7579
**20**	0.7560	0.7547	0.7580
**21**	0.7572	0.7554	0.7577
**22**	0.7567	0.7550	0.7589

	**B2PLYP**	**PBE0-DH**	**B2GP-PLYP**	**ωB2PLYP**	**ωB88PP86**	**Pr^{2}SCAN50**
**1**	0.7865	0.7881	0.7994	0.7848	0.8086	0.8062
**2**	0.7769	0.7784	0.7843	0.7757	0.7904	0.7894
**3**	0.7636	0.7641	0.7672	0.7646	0.7712	0.7685
**4**	0.7629	0.7638	0.7662	0.7633	0.7700	0.7671
**5**	0.7675	0.7679	0.7749	0.7698	0.7832	0.7773
**6**	0.7656	0.7664	0.7720	0.7671	0.7788	0.7721
**7**	0.7717	0.7709	0.7791	0.7776	0.7836	0.7764
**8**	0.7674	0.7678	0.7757	0.7704	0.7850	0.7779
**9**	0.7666	0.7669	0.7743	0.7695	0.7829	0.7767
**10**	0.7609	0.7598	0.7648	0.7630	0.7655	0.7622
**11**	0.7650	0.7653	0.7721	0.7670	0.7794	0.7732
**12**	0.7552	0.7549	0.7565	0.7558	0.7568	0.7560
**13**	0.7608	0.7611	0.7649	0.7615	0.7690	0.7665
**14**	0.7710	0.7720	0.7839	0.7737	0.7973	0.7830
**15**	0.7662	0.7672	0.7743	0.7681	0.7829	0.7741
**16**	0.8762	0.8680	1.0461	1.0305	1.1700	0.9700
**17**	0.8913	0.8819	1.0701	1.0504	1.1762	0.9854
**18**	0.7647	0.7657	0.7717	0.7654	0.7788	0.7722

**19**	0.7616	0.7623	0.7650	0.7620	0.7689	0.7659
**20**	0.7622	0.7628	0.7665	0.7630	0.7708	0.7670
**21**	0.7674	0.7677	0.7770	0.7694	0.7852	0.7762
**22**	0.7661	0.7663	0.7746	0.7679	0.7821	0.7741

References

[1] A. Cervilla, E. Llopis, D. Marco, F. Pérez, X-ray Structure of $(Bu^{n}_{4}N)[Mo(1,2\text{-}Benzenedithiolate)_3]$. Trigonal-Prismatic versus Octahedral Coordination in Tris(1,2-Benzenedithiolate) Complexes, *Inorg. Chem.* **2001**, *40*, 6525-6528.

[2] J. R. Bradbury, M. F. Mackay, A. G. Wedd, The crystal and molecular structure of tetraphenylarsonium Tetrakis(benzenethiolato)oxomolybdate(V), *Aust. J. Chem.* **1978**, *31*, 2423-2430.

[3] S. Boyde, S. R. Ellis, C. D. Garner, W. Clegg, Structural comparison of oxobis(benzene-1,2-dithiolato)molybdenum-(V) and -(IV) complexes, *J. Chem. Soc., Chem. Commun.* **1986**, 1541-1543.

[4] K. Peariso, B. S. Chohan, C. J. Carrano, M. L. Kirk, Synthesis and EPR Characterization of New Models for the One-Electron Reduced Molybdenum Site of Sulfite Oxidase, *Inorg. Chem.* **2003**, *42*, 6194-6203.

[5] M. L. Mader, M. D. Carducci, J. H. Enemark, Analogues for the Molybdenum Center of Sulfite Oxidase: Oxomolybdenum(V) Complexes with Three Thiolate Sulfur Donor Atoms, *Inorg. Chem.* **2000**, *39*, 525-531.

[6] S. C. Drew, J. P. Hill, I. Lane, G. R. Hanson, R. W. Gable, C. G. Young, Synthesis, Structural Characterization, and Multifrequency Electron Paramagnetic Resonance Studies of Mononuclear Thiomolybdenyl Complexes, *Inorg. Chem.* **2007**, *46*, 2373-2387.

[7] B. S. Lim, M. W. Willer, M. Miao, R. H. Holm, Monodithiolene Molybdenum(V,VI) Complexes: A Structural Analogue of the Oxidized Active Site of the Sulfite Oxidase Enzyme Family, *J. Am. Chem. Soc.* **2001**, *123*, 8343-8349.

[8] C. D. Garner, L. H. Hill, F. E. Mabbs, D. L. McFadden, A. T. McPhail, Crystal and molecular structure, electron spin resonance, and electronic spectrum of tetraphenylarsonium tetrachloro-oxomolybdenum(V), *J. Chem. Soc., Dalton Trans.* **1977**, 853-858.
